# Training healthcare professionals to administer Goal Attainment Scaling as an outcome measure

**DOI:** 10.1186/s41687-024-00704-0

**Published:** 2024-02-26

**Authors:** Benignus Logan, Andrea K. Viecelli, Elaine M. Pascoe, Bonnie Pimm, Laura E. Hickey, David W. Johnson, Ruth E. Hubbard

**Affiliations:** 1 Centre for Health Services Research, University of Queensland, 34 Cornwall St, Woolloongabba, Brisbane, QLD 4102 Australia; 2 Australasian Kidney Trials Network, University of Queensland, Brisbane, Australia; 3 Department of Kidney and Transplant Services, Princess Alexandra Hospital, Ipswich Rd, Woolloongabba, Brisbane, QLD 4102 Australia; 4 Centre for Kidney Disease Research, Translational Research Institute, Brisbane, Australia; 5 Department of Geriatric Medicine, Princess Alexandra Hospital, Brisbane, Australia

**Keywords:** Chronic kidney disease, Frailty, Goal Attainment Scaling, Outcome measure, Training, Randomised controlled trial

## Abstract

**Background:**

Goals generated by Goal Attainment Scaling (GAS) can be used as an outcome measure to promote person-centred research and care. There are no training packages which support its use outside of the rehabilitation discipline. This paper describes the development and evaluation of a training package to support the implementation of GAS as an outcome measure in healthcare research. The training package consisted of classroom teaching, a training manual for self-directed learning, one-on-one simulation and hot reviews. It was developed for the GOAL Trial, a randomised controlled trial assessing a Comprehensive Geriatric Assessment’s effectiveness in enabling frail older people living with chronic kidney disease to attain their goals. Training participants were invited to complete pre- and post-training online evaluation surveys.

**Results:**

Forty-two healthcare professionals attended an initial online classroom teaching, with 27 proceeding to administer GAS to GOAL Trial patients. Response rates for the online pre- and post-training surveys were 95% and 72%, respectively. Prior to training, only 15% of participants reported being able to appropriately scale and troubleshoot GAS goals. Post-training this was 92%. There was 100% participant satisfaction for the training manual, one-on-one simulation, and hot reviews.

**Conclusions:**

This training package helps ensure healthcare professionals administering GAS have adequate knowledge and skills. It has the potential for adoption as a guide to support the implementation of GAS by other researchers seeking to embrace persont-centred principles in their work.

**Supplementary Information:**

The online version contains supplementary material available at 10.1186/s41687-024-00704-0.

## Background

Goal Attainment Scaling (GAS) is a method to set and then score the extent to which a person’s individual goals are achieved [1, 2]. As well as allowing scoring for an individual, it offers the ability to compare the attainment of goals across heterogeneous individuals and groups [3–5]. The goals generated from the GAS process can be utilised by researchers as a patient-reported outcome measure (PROM) to support person-centred care, which is a recognised pillar of quality healthcare and research [1, 6, 7].

**Fig. 1:**
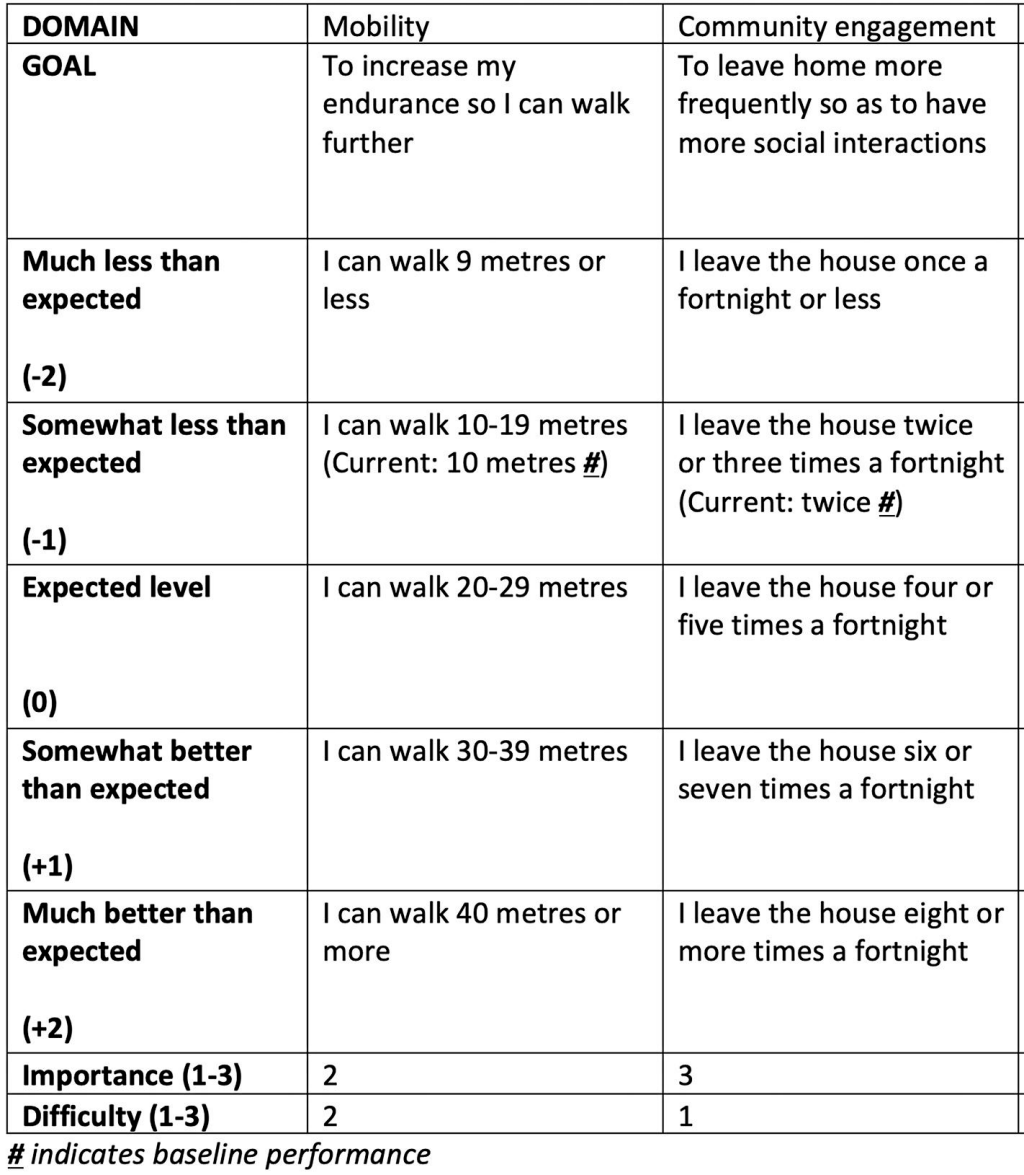
Example of baseline GAS goals

The GAS process includes: identifying an individual’s goal; describing their current abilities; defining possible future outcomes on a five-point scale; and, at follow-up, scoring their actual achievement against the articulated scale [1]. For each goal, achievement is scored as a number value between −2 and +2. If the patient achieves the expected level, this is scored at 0. If they achieve a better outcome this is scored at +1 (somewhat better than expected) or +2 (much better than expected). If they achieve a worse outcome this is scored at −1 (somewhat less than expected) or −2 (much less than expected). A participant’s baseline performance is typically scored as the −1 level. Each goal has a self-assigned weighting from the participant for its importance and difficulty on a scale of 1–3, where three is extremely important/difficult. Figure 1 provides a worked example.

Our prior scoping review [8] demonstrated that whilst GAS has been used widely in a variety of randomised controlled trials (RCT), there have been inadequacies and inconsistencies in its application. Concerns with the implementation of GAS include suboptimal facilitator knowledge [9, 10] and poorly written goals and scales [11, 12]. It has been argued that GAS’ validity and reliability would be better served by third-party review of goals, and ensuring there is adequate facilitator training and articulation of how GAS has been practically implemented [5, 9, 11–15].

It has been suggested that training procedures for GAS should be developed before it is further used as an outcome measure in effect studies [16]. To date, the only published standardised training guidance which exists is from Turner-Stokes [10], Steenbeek [16] and Bovend’Eerdt [17]. Whilst well regarded, they do not provide a comprehensive suite of learning aids that can be easily adapted and deployed by researchers to help potential facilitators understand how to administer GAS. They are also each written for the rehabilitation setting, which hinders the ability for researchers to utilise it in different disciplines given they focus primarily on physical and functional reconditioning and reablement.

People living with chronic kidney disease have priorities which extend beyond mobility and physical functioning. They include life participation, decrease in blood pressure, impact on family, and managing symptoms of anxiety, depression and a lack of appetite [18, 19]. Using GAS as a PROM for people living with chronic kidney disease is important as outcome measures in trials in this population can more frequently focus on biochemical measures, such as anaemia and electrolyte results, as opposed to the less frequently reported but more patient-prioritised outcomes of quality of life [20]. PROMs, like GAS, allow an appreciation to be gained of what matters most to an individual [7, 21].

Steps taken to aid GAS’ implementation need to be feasible. Feasibility is the measure of how successfully an innovation, such as a training package, can be employed in a specific environment [22]. A surrogate marker for whether this is achieved can include the perceptions of individuals involved in the delivery of it [23, 24].

The aim of this research was to: (1) describe the development of a training package for GAS as an outcome measure which has been implemented in a multi-site cluster RCT; and, (2) evaluate the effectiveness of this training package in preparing healthcare professionals to feel confident and competent in administering GAS with research participants who are frail older people.

## Methods

### Study setting

The GOAL Trial is a cluster RCT, whose protocol has been published elsewhere [25]. Participants are frail older people living with moderate to severe chronic kidney disease (eGFR < 59 mL/min/1.73 m^2^) recruited from nephrology outpatient clinics of public hospitals (the clusters) across Australia. The trial has a two-arm design, intervention and control, with a 1:1 allocation of the 16 clusters. Target recruitment is 500 participants. Those in an intervention cluster receive a Comprehensive Geriatric Assessment (CGA) from a geriatrician, who develops a management plan following an assessment of an older person’s medical, social, and functional needs [26, 27]. The control group receives usual care.

The primary outcome is attainment of participant’s self-identified goals assessed (GAS) at 3 months whereby achieving the ‘Expected level (0)’, or better, means the goal was accomplished (see Fig. 1). Secondary outcomes include quality of life (measured by EQ-5D-5L [28]), frailty, hospital admissions, transfer to residential aged care facilities and cost-effectiveness. A process evaluation of the intervention is also being conducted.

During the baseline study visit, GOAL Trial participants complete GAS where they set between one and five goals of their choosing. In preparation for this, they are issued with a patient preparation information sheet (Supplementary File A) to prompt self-reflection. This document highlights the benefits of goal setting and lists possible areas in which they may wish to set goals. A support person is welcome to attend to prompt reflection and provide important collateral history to inform what is realistic and achievable.

GAS is administered by a healthcare professional, typically the research nurse who is the site coordinator. In the initial stages of the trial’s development, it was established that most sites had no familiarity with GAS. To help guarantee GAS would be a feasible outcome measure, a training package needed to be developed to ensure healthcare professionals were competent and confident to administer it. A training package is a structured set of materials and resources to facilitate the learning process.

The trial is funded by a National Health and Medical Research Council Targeted Call for Research into Frailty in Hospital Care (APP1178519). The study sponsor is The University of Queensland acting through the Australasian Kidney Trials Network (AKTN). Ethics approval was obtained from the Metro South Human Research Ethics Committee (Reference: HREC/2020/QMS/62883). Recruitment was open March 2021 to July 2023, with the 12-month follow-up due completion by July 2024.

### Training package development

To inform training package development, a systematic scoping review of GAS was completed to understand how it has been implemented in prior RCTs [8]. This was complemented by conversations with Australian and international researchers who had used GAS as an outcome measure, to understand their real-world learnings and insights. Each part of the training was written with the intent of addressing the previously identified barriers to GAS’ implementation such as suboptimal facilitator knowledge, poorly written goals and scales, and the lack of third-party review [9–12].

Learning theory comprises principles that inform how students acquire and retain information, and active learning frameworks underpin what motivates students to actively participate in learning [29–31]. This was considered in how the various components of the training package were designed. The material developed adhered to Biggs’ constructive alignment [32] which postulates that learning outcomes, activities and assessments are aligned to be complementary to each other. This was particularly achieved in the simulation scenarios which were embedded in teaching. Mayer’s multimedia learning theory advocates that computer-based content should minimise extraneous load, manage intrinsic load and optimise germane load [33]. As a result, the PowerPoint slides and videos created had visuals that were simple and well signposted, narration was with a human voice, and language was casual and avoided professional jargon.

Consumer-facing materials were reviewed by members of the trial’s Consumer Advisory Board. Prior to the training package being used, it was piloted with two research nurses on a smaller single-site trial being led by one of our team (REH) where GAS was an outcome measure. No changes were required from this pilot.

The components of the training package developed are summarised in Table 1. The training was delivered by a Lead trainer (BP) who is a Clinical Nurse, and a PhD student (BL) who is a doctor. The healthcare professionals began training by attending online classroom teaching in small groups of one to five participants. This covered an introduction to the importance and benefits of goal setting, an overview of SMART (Specific, Measurable, Achievable, Relevant, and Time-Bound) goals and the GAS template, instructions on how to set a goal for GAS, recommendations on how to conduct the meeting, and troubleshooting suggestions for common challenges. It concluded with scenario-based practical simulations for active learning (Supplementary Files B and C).

**Table 1:** Training package components

Training component	Associated resources	Personnel	Timing
Formal online classroom teaching	Training slides and trainer notes (Supplementary File B) Guide notes for simulated scenario-based learning (Supplementary File C)	Lead trainer	2 h
Self-directed learning	A practical guide to administering Goal Attainment Scaling for the GOAL Trial Included appendices: – example of populated GAS template – conversation starter guide – example of populated conversation starter guide (Supplementary File D) Recording of slide presentation from GAS classroom teaching (Supplementary File E) Recording of an example simulation of an initial goal setting meeting (Supplementary File E)	PhD student	3 h
One-on-one simulation and feedback session	Simulation scenario for one-on-one feedback session (Supplementary File F)	Lead trainer	30 min
Hot review	Five completed GAS worksheets for each healthcare professional completing the training package	PhD student	15 min each

Participants undertook their own self-directed learning, supported by a comprehensive training manual, “A practical guide to administering Goal Attainment Scaling (GAS) for the GOAL Trial” (Supplementary File D). They also could access recordings of the classroom session and an example goal setting meeting (Supplementary File E). Within two weeks of the classroom teaching, they underwent one-on-one simulation with the Lead trainer (Supplementary File F). This allowed for more high-fidelity GAS facilitator practice, was a mechanism to ensure proficiency could be demonstrated, and enabled consistency between GAS facilitators across multiple sites.

**Fig. 2:**
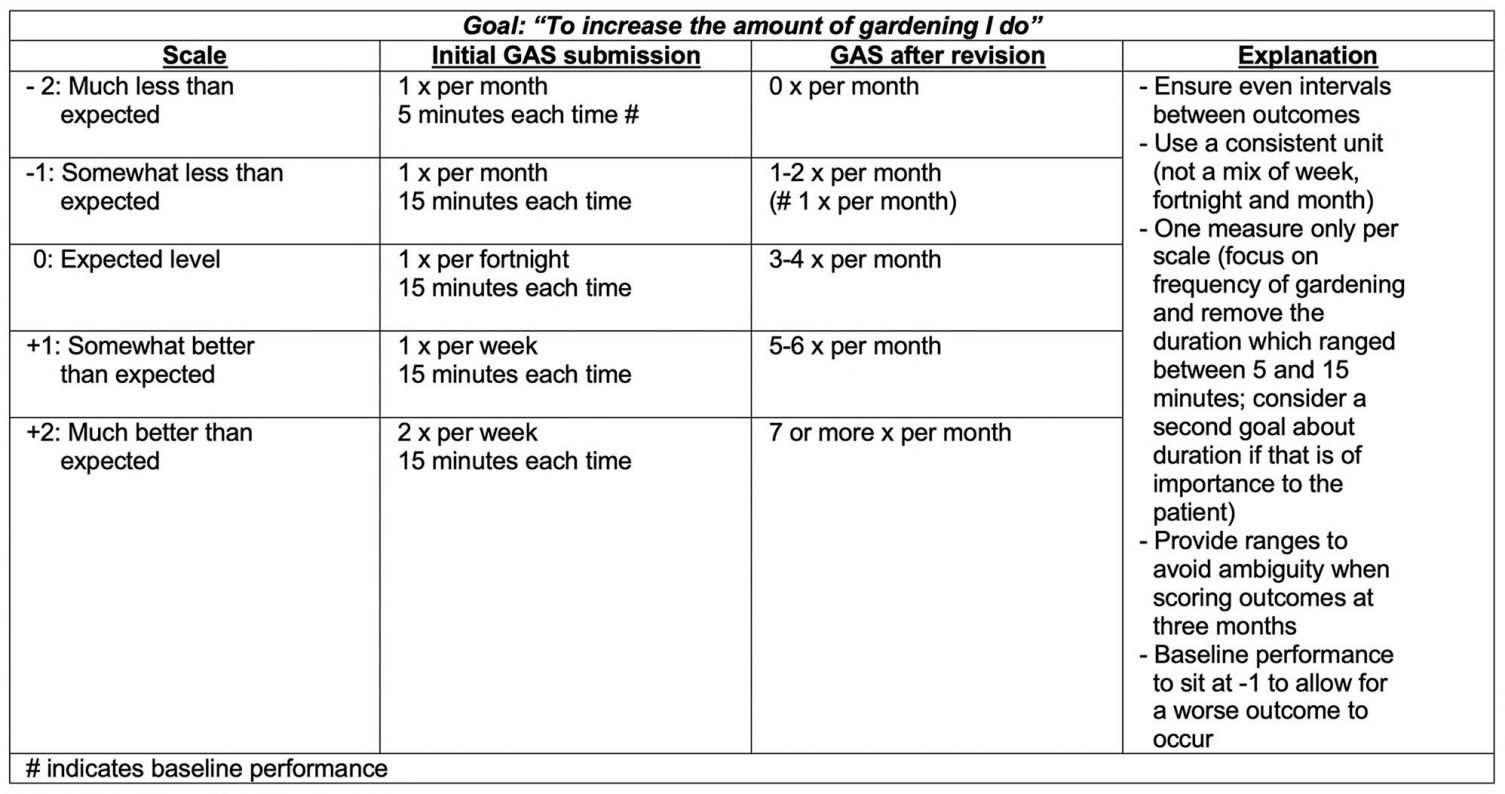
Example of a GAS hot review

The first five GAS conducted were sent for a hot review. The term ‘hot review’ was formulated by us to denote the third-party review instituted where the trial’s PhD student (BL) would review the appropriateness of the measures and outcome scaling within a 24-hour turnaround period. This helped ensure the healthcare professional’s proficiency when they began to facilitate GAS with actual trial participants. Figure 2 provides a worked example.

For GAS and the associated training package to be feasible in this trial, its successful implementation required the satisfaction of those healthcare professionals tasked with administering it. Success in such situations can be threatened by poor readiness for change, feelings of uncertainty and a lack of control, and difficulty in using the tool [22, 34]. The training package’s components were designed mindful of addressing this by using clear and structured communication, and embedding simulations to promote self-efficacy and reduce uncertainty [34].

### Evaluation of training package

To evaluate the training program’s success in preparing healthcare professionals to administer GAS to participants, a sub-study was undertaken concurrently with the primary GOAL Trial. Approval was given by the Metro South Human Research Ethics Committee (HREC/2020/QMS/70496). The primary hypothesis was that comprehensive training would improve healthcare professionals’ self-reported preparedness to administer GAS. This was chosen as a pragmatic surrogate marker for testing how effective the training had been in aiding the improvement of facilitator knowledge.

**Fig. 3:**
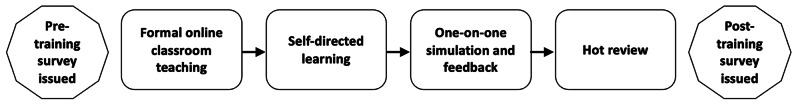
Data collection process

Data were collected via two online surveys on Qualtrics [35] (Fig. 3). The survey (provided in Supplementary File G) included questions asking participants to self-rate their ability to write and scale a GAS goal, structure a goal-setting conversation, and troubleshoot a goal which does not meet GAS requirements. The first survey was issued prior to the formal online classroom teaching. The second survey was sent to participants after five hot reviews, which marked completion of training. One reminder email to complete the post-training survey was sent around a week after it was initially issued.

In designing the survey, consideration was given to the Kirkpatrick model which is a method to appraise workplace training [36, 37]. The survey undertaken addressed the first two levels of this model: ‘Reaction’ and ‘Learning’. Firstly, the reaction level was addressed by asking participants to rate on a five-point scale their satisfaction with each element of the program (classroom teaching, training manual, hot review etc). Satisfaction of the healthcare professionals was essential as GAS was the GOAL Trial’s primary outcome and the facilitators were integral to its successful deployment. It has been recognised that the perception and satisfaction of those individuals involved in delivering a tool such as GAS are key to its feasibility [23, 24]. Secondly, the learning level was partially addressed through assessment of the training participants’ self-reported preparedness. Their perceived preparedness reflected an attitude change.

The consent process was undertaken electronically via a Consent Statement displayed seeking the agreement of the training participants (Supplementary File H). Survey completion was voluntary and without undue pressure. An independent Clinical Research Associate redacted any identifying details before data were shared with investigators.

Responses to questions which required a yes/no answer or a rating on a five-point Likert Scale were analysed via simple descriptive statistics undertaken in Microsoft Excel. Comments from both surveys were to be imported into NVivo [38] for thematic analysis, but did not occur because of limited responses.

## Results

### Training delivery

**Fig. 4:**
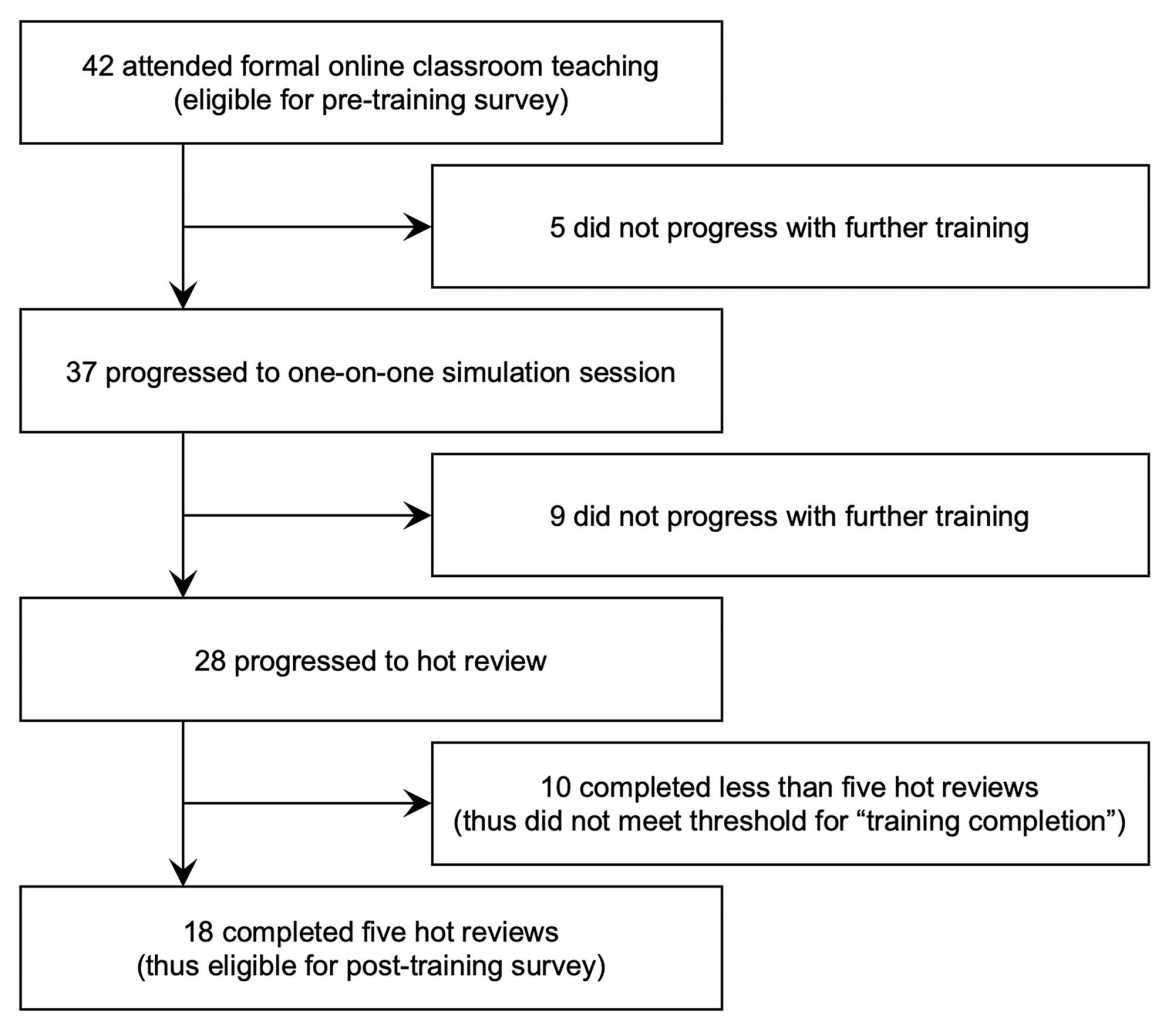
Participant flowchart

Training healthcare professionals to administer GAS was a key requirement for the GOAL Trial’s 16 study sites as part of initiation activities. Online classroom teaching was held between February 2021 and May 2023. The Lead trainer delivered 15 online classroom teaching sessions to a total of 42 participants. A private YouTube link to the classroom teaching recording was viewed 28 times, and the example initial goal setting meeting 34 times. The hot review process reviewed 123 completed GAS. The training package was only considered fully completed when a healthcare professional had sent five GAS worksheets for review. Only 18 of the initial 42 healthcare professionals met this threshold. Figure 4 provides a participant flowchart.

### Evaluation survey

The pre-training evaluation survey was completed by 95% (n = 40) of those who attended the initial online classroom teaching. The post-training survey had a 72% response rate (13 out of an eligible 18). As shown in Table 2, most training participants did not have any prior awareness of GAS (83%, n = 33) nor any first-hand experience of the GAS process (98%, n = 39). There was an awareness of SMART goals for many (68%, n = 27), and the majority (53%, n = 21) had experience in setting their own SMART goals. Half (50%, n = 20) of the respondents indicated they had some prior experience in helping someone set a goal.

**Table 2:** Participant’s pre-existing knowledge

Area of prior knowledge	Frequency, n (%) (total respondents: 40)	
	Yes	No
Had heard of GAS before being engaged in the GOAL Trial	7 (18%)	33 (82%)
Had used GAS before (either as a facilitator or participant)	1 (2%)	39 (98%)
Had heard of SMART goals before	27 (68%)	13 (32%)
Had set their own SMART goals before	21 (52%)	19 (48%)
Had facilitated someone to set goals before	20 (50%)	20 (50%)

Trainee participants recognised the importance of goal setting. Ninety-five percent of respondents (n = 38) either agreed or strongly agreed with the statement that goal setting was important to patient-centred care. There was also 95% (n = 38) agreement that goal setting benefitted a patient’s health and quality of life. Prior to training, participants’ self-reported ability in key aspects of facilitating GAS was low (Table 3). Many reported either disagreeing or feeling neutral about their abilities to structure a conversation with a patient to set their goals (58%, n = 23), defining an outcome measure for a goal (53%, n = 21), or writing a SMART goal (46%, n = 18). The areas of lowest self-reported confidence, indicated by low levels of agreement or strong agreement, was scaling goals to meet GAS template requirements (15%, n = 6), troubleshooting a patient’s desired goal which did not meet GAS requirements (15%, n = 6), and working with a patient who engaged poorly with the process (22%, n = 8).

**Table 3:** Self-reported confidence

I feel able to:	Pre-training survey n (%) (40 total respondents)						Post-training survey n (%) (13 total respondents)
	Strongly Disagree	Disagree	Neutral	Agree	Strongly Agree	Agree + Strongly Agree	Agree + Strongly Agree
Write a SMART goal	5 (13%)	4 (10%)	9 (23%)	17 (44%)	4 (10%)	21* (54%*)	12 (92%)
Define an outcome measure for a goal	2 (5%)	4 (10%)	15 (37.5%)	15 (37.5%)	4 (10%)	19 (48%)	12† (100%†)
Scale a goal to meet the requirements of the GAS template	8 (20%)	10 (25%)	16 (40%)	4 (10%)	2 (5%)	6 (15%)	12 (92%)
Structure a conversation with a patient to set their goals	2 (5%)	3 (7.5%)	18 (45%)	13 (32.5%)	4 (10%)	17 (43%)	13 (100%)
Troubleshoot a patient’s desired goal which does not meet the GAS requirements	7 (17.5%)	11 (27.5%)	16 (40%)	4 (10%)	2 (5%)	6 (15%)	12 (92%)
Work with a patient who has poor engagement with the process	4 (11%)	6 (16%)	19 (51%)	4 (11%)	4 (11%)	8‡ (22%‡)	9 (69%)

*Notes* *39 respondents; †12 respondents; ‡37 respondents

The self-reported confidence of respondents in the post-training evaluation was much higher than prior to training. With the exception of one element, there was 92% or higher agreement regarding their ability to complete key aspects of the GAS process. The area which scored lower (69%, n = 9) was self-reported ability to work with a patient who was poorly engaged. As the surveys were linked and only 13 of the initial 40 pre-training survey respondents completed a post-training survey, it is unclear whether those responding favourably in the post-training survey had responded equally favourably in the pre-training survey, or there was a reported improvement at an individual level.

The training package was well regarded by training participants, with a very high frequency of respondents being either satisfied or very satisfied with each component (Table 4). The free-text responses to the questions (Table 5) highlighted particular satisfaction with hot reviews.

**Table 4:** Participant satisfaction

Training component	Post-training survey Satisfied or very satisfied n (%) (13 total respondents)
Formal online classroom teaching	12 (92%)
Training manual (“A practical guide to administering GAS for the GOAL Trial”)	13 (100%)
Video recording of example goal-setting conversation	11 (85%)
One-on-one simulation and feedback	13 (100%)
Hot review	13 (100%)

**Table 5:** Participant comments

Comments submitted in the post-training evaluation survey
**Related to patient interactions:** – All patients so far have been very keen to participate and love to hear that this is something all about them. – It can be difficult to set SMART goals with some people—highlighting that these goals have to be “realistic” and “measurable” have helped hone in on relevant ideas. – It has been difficult to conduct frailty assessments and set goals with participants in the control arm. The process gives us great insight into how we may help these people live better/subjectively healthier lives, but there is very little we can do to help them achieve these goals during the trial.
**Related to hot reviews and other training supports:** – The hardest part is making the goals fit the smart criteria but the hot review has been very helpful for this. – Definitely need the hot review as it can be tricky to make some goals SMART – The reflective process of the hot review was particularly helpful, as feedback was received in a timely and helpful manner. – At times making a patient’s GOAL SMART was difficult and I appreciated the help of <training staff> with this. – The support offered was very helpful.

## Discussion

This research shows it is possible to use GAS as an outcome measure for an RCT in a population of people living with chronic kidney disease, which has not occurred previously [8]. It also demonstrates a GAS training package was able to be developed and implemented in a multi-centre trial in a discipline outside of rehabilitation. A training package was necessary for the GOAL Trial as most participants had neither prior awareness of GAS nor any direct experience, and many had no prior experience in facilitating goal setting. More broadly, having a training package available for researchers to utilise is important as our scoping review [8] showed that 78% of trials using GAS did not state what training supported its implementation, and 42% of studies were outside of the rehabilitation discipline where training is not directly able to be supported by Turner-Stokes’ well regarded guide [10].

The training package developed for the GOAL Trial, including the hot review process, addressed some of the identified critiques of how GAS is applied [5, 9, 11, 12, 15]. The formal online classroom teaching sought to equip healthcare professionals with knowledge so that they avoid the common flaws identified by Krasny-Pacini and colleagues, which included goals with more than one variable and attainment outcomes that were too subjective or overlapping [12]. The simulation scenarios and hot review process allowed for feedback loops to further embed healthcare professionals’ learning and ensured revision of poorly constructed GAS.

The results of the evaluation survey provided insights into the views of the healthcare professionals who were responsible for facilitating GAS with GOAL Trial participants. This is of value given their perspective on the success of the delivery of a tool, such as GAS training, can be considered as a marker of its feasibility [23, 24]. The improvement noted in self-reported ability to appropriately scale and troubleshoot goals, along with high satisfaction with the training materials, may be interpreted as evidence of successful implementation. However, this is tempered by the relatively low completion rate for the training package and only a 72% response rate for the post-training evaluation survey.

The only post-training ability with under 90% agreement was working with a patient who had poor engagement. This highlights that more support may be required to address this. Given how well received the hot review process was, it may have been beneficial for this to be completed for all GAS and not limited to the first five undertaken by each healthcare professional.

The significance of this training package needs to be considered in the broader context of the important role PROMs, like GAS, play in research. When PROMs are not considered, the bigger picture for an individual can be missed, especially for those with multiple health concerns where care is provided in a fragmented manner across different teams [7, 21]. People living with chronic kidney disease, the target population for the GOAL Trial, typify multi-morbid individuals with complex needs [39].

The use of GAS aligns with the growing consensus regarding the need for PROMs in healthcare settings that interface with frailer, older people so that what matters most to them is measured [40]. Prior trials of interventions in this population favour traditional measures that are routinely captured and well understood, such as hospital admissions and functional status [40]. Recent guidance from the US Food and Drug Administration [41] advocates for measures, such as GAS, to be used to incorporate the patient’s voice in regulatory decision-making on drug development.

Building on the broad principles set out in the work of Reuben and Tinetti [21, 42], there is a growing interest in better implementing GAS. Krasny-Pacini’s group have sought to aid GAS’ implementation in rehabilitation settings by developing an educational review and toolbox [43]. This provides practical insights which supplement the existing guidance from Turner-Stokes [10], Steenbeek [16] and Bovend’Eerdt [17]. Stolee’s recent feasibility study looked at how lay interviewers could be used to help facilitate GAS in primary care given that clinicians are time-poor in those settings and may be unable to play that role [44]. They also developed an inventory of goals they propose can be used as a starting point for others who seek to work with patients to self-identify goals in primary care, with the hope that it makes the process more efficient [44]. This complements prior work to articulate a standardised menu of goals for frailer, older people [45], with the use of pre-specified goals being shown to be feasible for operationalising in a clinical setting [46]. Future work from our group’s GOAL Trial will produce an inventory of goals relevant to frailer, older people with chronic kidney disease.

The training package described in this paper makes a further contribution to these recent endeavours to better implement GAS. It is not constrained by a rehabilitation lens, which has a primary focus on reablement and physical functioning. By being more holistic in its content and outlook, researchers in other disciplines and populations can more easily utilise it as a framework to facilitate person-centred care and outcomes. The comprehensive nature of the training package also provides greater consistency in the content and key messages which are being delivered to those healthcare professionals intending to administer it, regardless of their background and existing knowledge. This is particularly beneficial for projects, like the GOAL Trial, that are geographically dispersed. To practically aid this training package’s use by other researchers, it has been published and is freely accessible on the University of Queensland website (https://www.afn.org.au/for-researchers/gas/) with the specifics of the GOAL Trial removed [47].

There are a number of limitations to this paper. Firstly, alignment of the GAS training’s evaluation component of the research could have more comprehensively considered the principles of implementation science and feasibility beyond assessing the self-reported preparedness of those healthcare professionals who facilitated GAS. The limited scope taken was a pragmatic decision as resources were prioritised for developing and activating the primary GOAL Trial which occurred during the early disruptive stages of the COVID pandemic.

Secondly, only 43% of the healthcare professionals who commenced the training completed the package. This was partly due to turnover of study site staff and fewer staff required to complete baseline GAS due to lower than anticipated participant recruitment.

Thirdly, the training evaluation was restricted to online surveys. This meant the final two levels of the Kirkpatrick model (‘Behaviour’ and ‘Results) were not addressed. That would have required a comprehensive approach to formally assessing knowledge gains or skills acquirement in an objective manner via appraised one-on-one simulations before and after the training or examination of knowledge attainment. This was deemed to not be practicable for this trial given competing resource demands meant priority had to be given to the core activities of the main GOAL Trial rather than the training evaluation sub-study. The limitations of using online surveys were further compounded by a low number of post-survey responses with only 13 of an eligible 18 (72%) completing one. Additionally, a negligible amount of free text comment was provided to open-ended questions meaning qualitative analysis was not possible. The planned linkage of a respondent’s pre- and post-survey failed as participants either did not enter their name, or used a first name which was common to more than one participant. The low completion rate, particularly as only 13 of the original 42 attendees completed both surveys, does have implications for feasibility and the ensuing attrition bias does mean that we can only conclude that those who completed training and the post-training survey indicated a preparedness to administer GAS.

To address some of the limitations, the process evaluation work underway for the GOAL Trial will explore staff’s and consumers’ perspectives on GAS in tandem with their assessment of the intervention. These data are anticipated to be shared in peer-reviewed publications once the primary outcome paper is published in early 2025.

## Conclusion

The GAS training package developed for the GOAL Trial provides an approach which researchers may wish to use in their endeavours to better implement GAS as a PROM in future trials, and facilitate reproducibility. Having been written without a focus on goals related to rehabilitation, it can be utilised in settings where the nature of participant goals may be diverse and not limited to reablement and physical functioning. If adopted more broadly, this training package could support a degree of standardisation in applying GAS as a PROM and thus help increase the reliability and comparability of trial results.

### Electronic supplementary material

Below is the link to the electronic supplementary material.

Supplementary File A: GAS patient preparation information sheet

Supplementary File B: Training PowerPoint slides and trainer notes

Supplementary File C: Worksheets for scenario-based learning

Supplementary File D: A practical guide to administering Goal Attainment Scaling for the GOAL Trial

Supplementary File E: Links to video recordings

Supplementary File F: Worksheet for one-on-one feedback session

Supplementary File G: Survey questions

Supplementary File H: Consent Statement

## Data Availability

The datasets used and/or analysed during the current study available from the corresponding author on reasonable request.

## List of abbreviations

AKTN: Australasian kidney trials network
CGA: Comprehensive geriatric assessment
GAS: Goal Attainment Scaling
PROM: Patient-reported outcome measure
RCT: Randomised controlled trial
SMART: Specific, measurable, achievable, realistic, time-bound
