# Supplementary material for: Training healthcare professionals to administer Goal Attainment Scaling as an outcome measure
Source: J Patient Rep Outcomes. 2024 Feb 26;8:22. doi: 10.1186/s41687-024-00704-0 (PMC10897066; doi:10.1186/s41687-024-00704-0)
Supplement: Supplementary file 1 — Supplementary File A: GAS patient preparation information sheet [file 41687_2024_704_MOESM1_ESM.pdf]

GOAL
ENHANCING PATIENT-CENTRED CARE

Appointment details

Date: Time:

Location:

Research Nurse:

Contact Number:

We want to know about **YOUR** goals

When we next meet, we will be spending time together to learn more about you.

A key part of the GOAL Trial is setting goals with you. Don’t worry – it is not hard.

By setting goals:

- Your care can be focused on what you want - not just what your doctors choose for you,
- You and your healthcare providers can engage in shared decision-making, and
- You can identify and celebrate meaningful changes you make in your life.

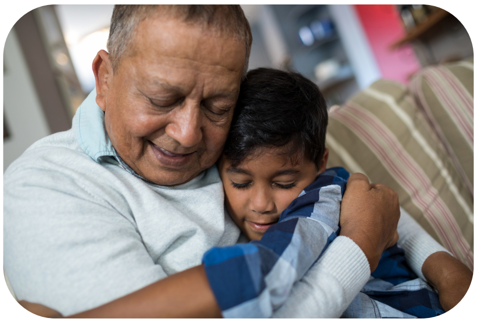

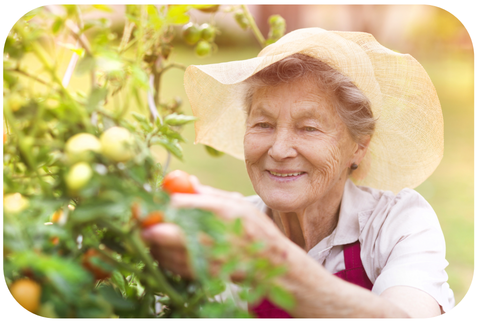

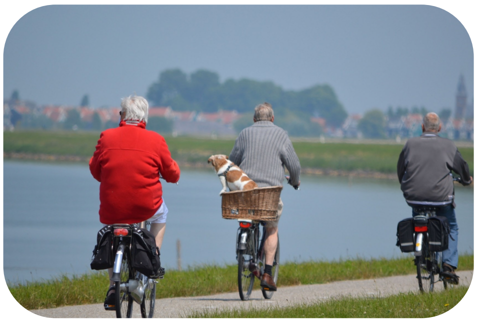

Before we meet, reflect on what parts of your life you may want to make better.
To help you, below are some areas people set goals in.

Physical health
- Medical conditions
- Medications (number, tolerability)
- Symptoms (such as fatigue or pain)
- Nutrition

Psychological health
- Cognition (memory, clarity of thought)
- Mood
- Resilience (your ability to cope)
- Sleep

Function
- Ability to mobilise (walk)
- Ability to care for yourself
- Transport

Planning
- Finances
- Plans for the future

Social engagement
- Personal relationship (spouse, dating)
- Relationships with your family
- Friendships
- Travel
- Employment and work roles
- Study
- Hobbies
- Community engagement (such as church groups or volunteering)

Your notes:

____

____

____

____

____
