# Supplementary material for: Training healthcare professionals to administer Goal Attainment Scaling as an outcome measure
Source: J Patient Rep Outcomes. 2024 Feb 26;8:22. doi: 10.1186/s41687-024-00704-0 (PMC10897066; doi:10.1186/s41687-024-00704-0)
Supplement: Supplementary file 2 — Supplementary File B: Training PowerPoint slides and trainer notes [file 41687_2024_704_MOESM2_ESM.pdf]

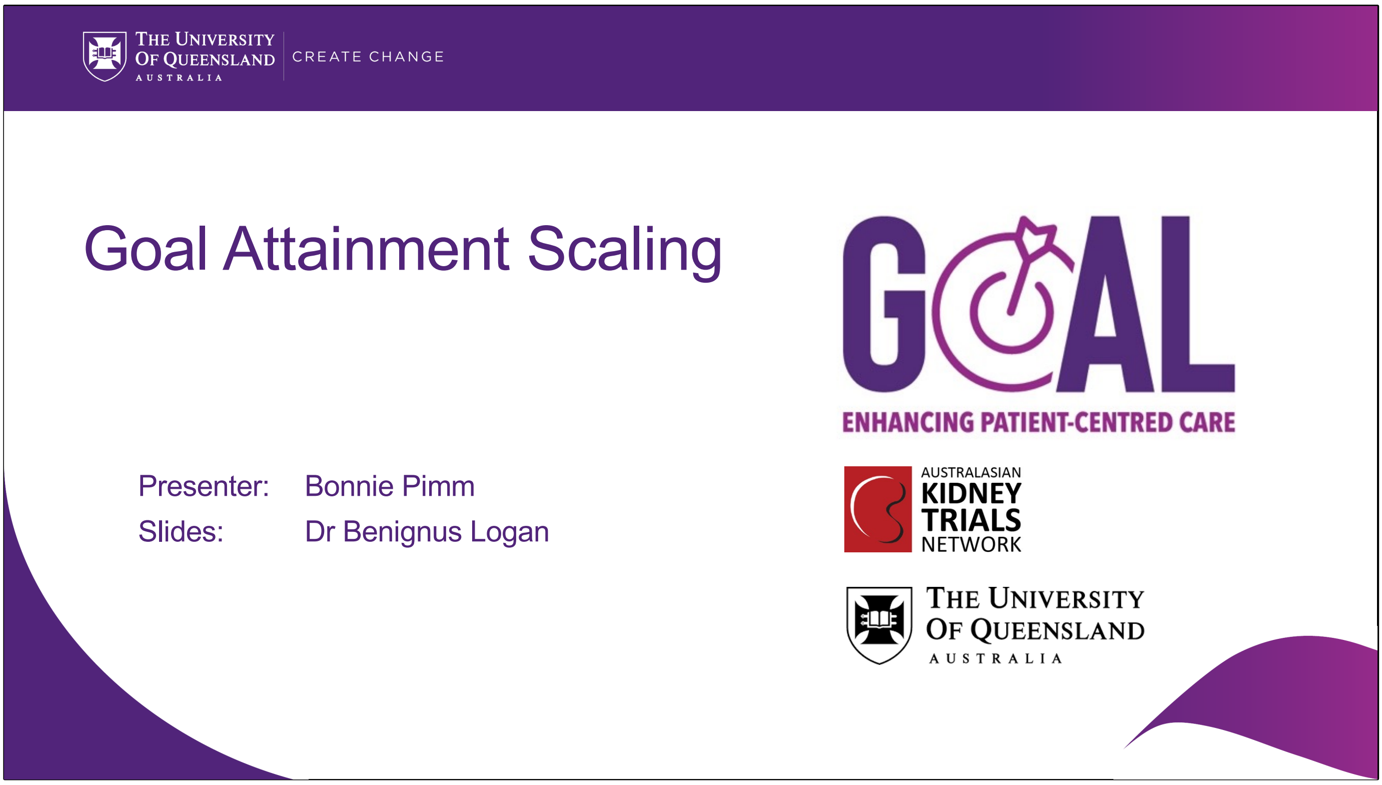

Actions prior to commencing the training:

- Send each participant the GAS Training manual 3-5 days prior and suggest they print a copy should they wish to have a hardcopy to write notes on
- Advise participants they need their own computer for the training (works better not to share given the breakout groups)
- Allocate breakout groups, and update slide to reflect this
- Send out the simulation scenario files (instructions, and the relevant "role briefings" sheet) to each participant individually the morning of the training

____

Welcome!
Introduce self

Before we begin, can I check everyone has:

- A pen and paper, or an electronic notepad
- A copy of the material that was sent out either as a hard copy or an easily accessible soft copy

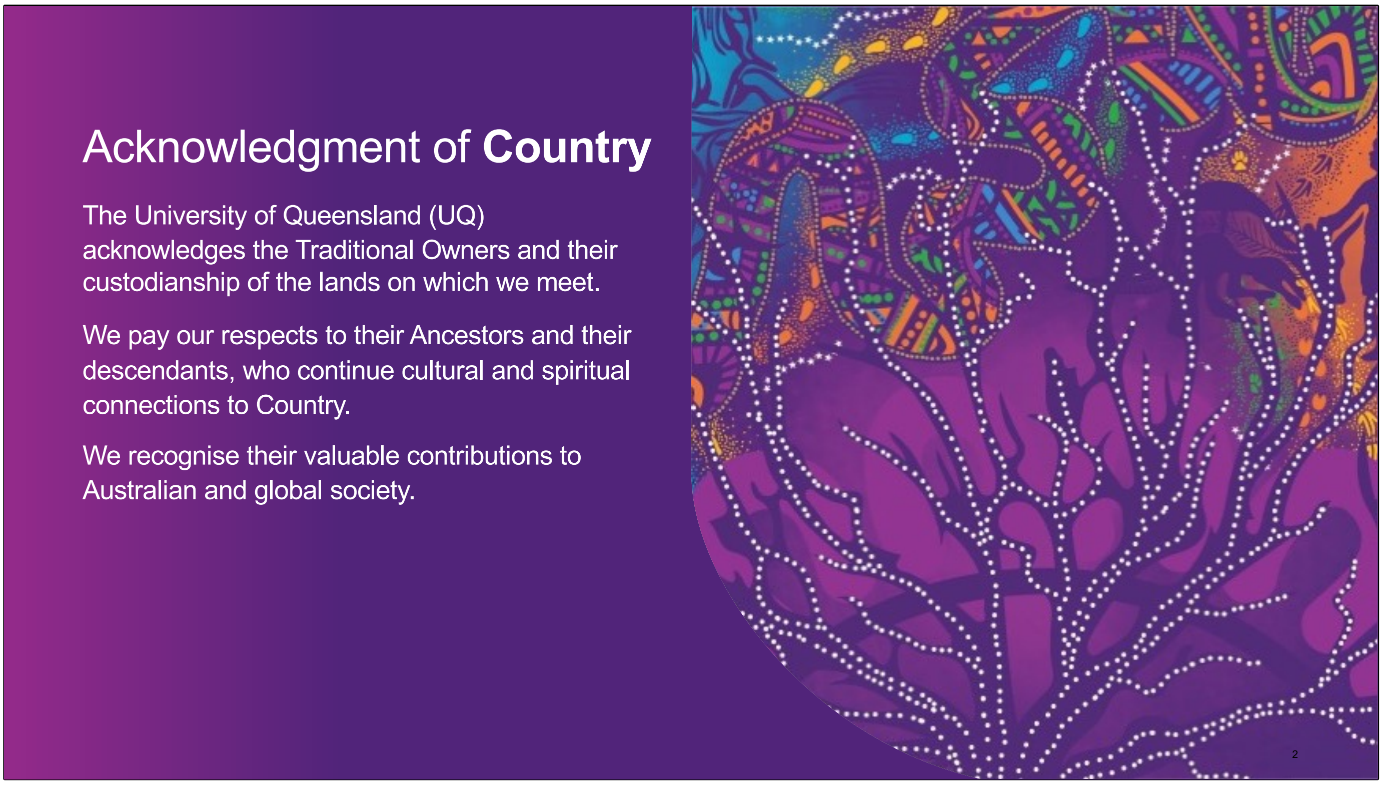

I acknowledge the Traditional Owners and their custodianship of the lands on which we meet today and pay my respect to their Ancestors and their descendants.

(Pause)

In the context of our research, I think it is important to reflect on the fact that A&TSI people are more likely, when compared to non-indigenous Australians, to have kidney failure, and to be hospitalised or die with CKD.

I also feel it is important to acknowledge the contribution which A&TSI make as doctors, nurses and researchers within the care of people with CKD.

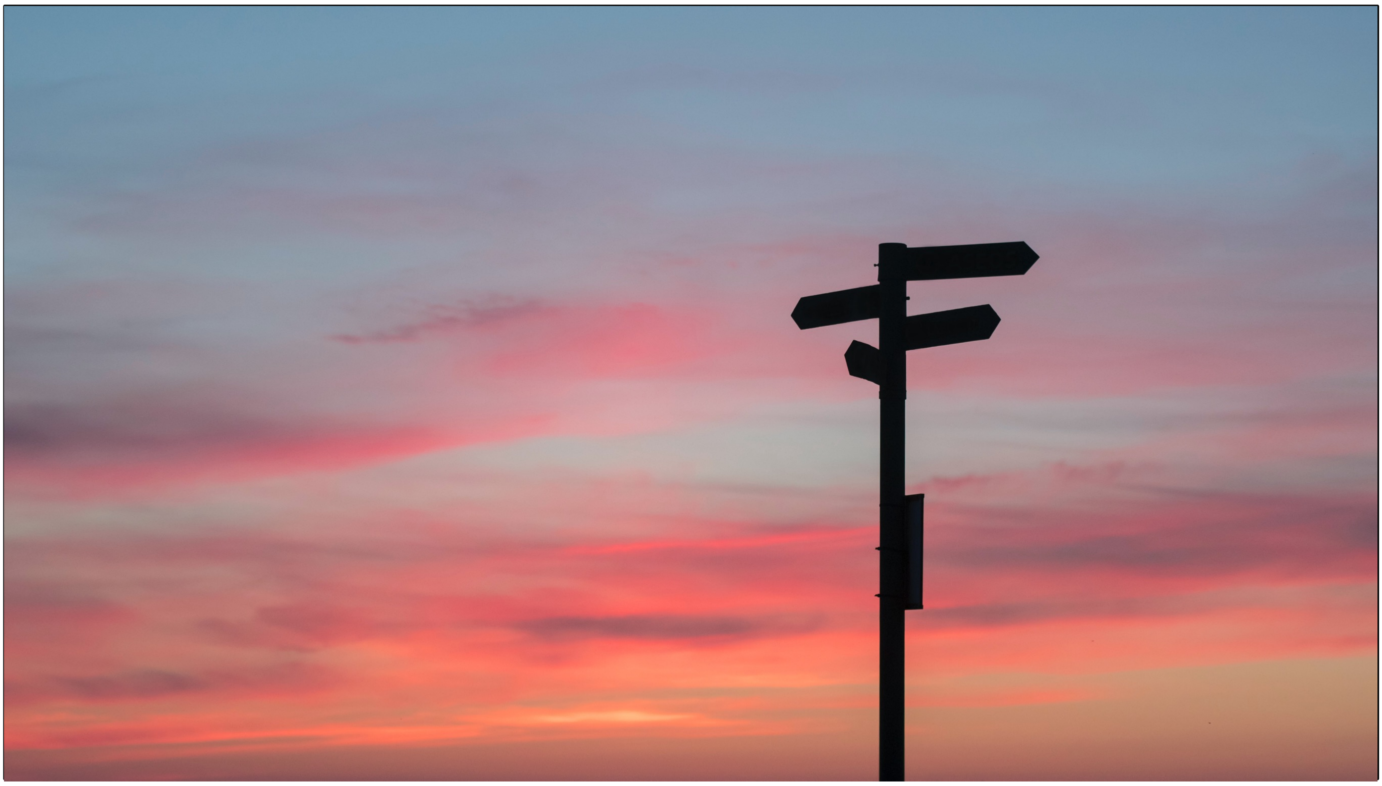

The purpose of this training is to prepare YOU to feel ready to complete the GAS process with patients.

Given today is all about you and your needs, I encourage you to stop me with any questions as they arise. Either I will answer them then or note them to cover later (whichever is more appropriate).

You may wish to have the GAS Training Manual open as we progress through today's training to write notes as required.

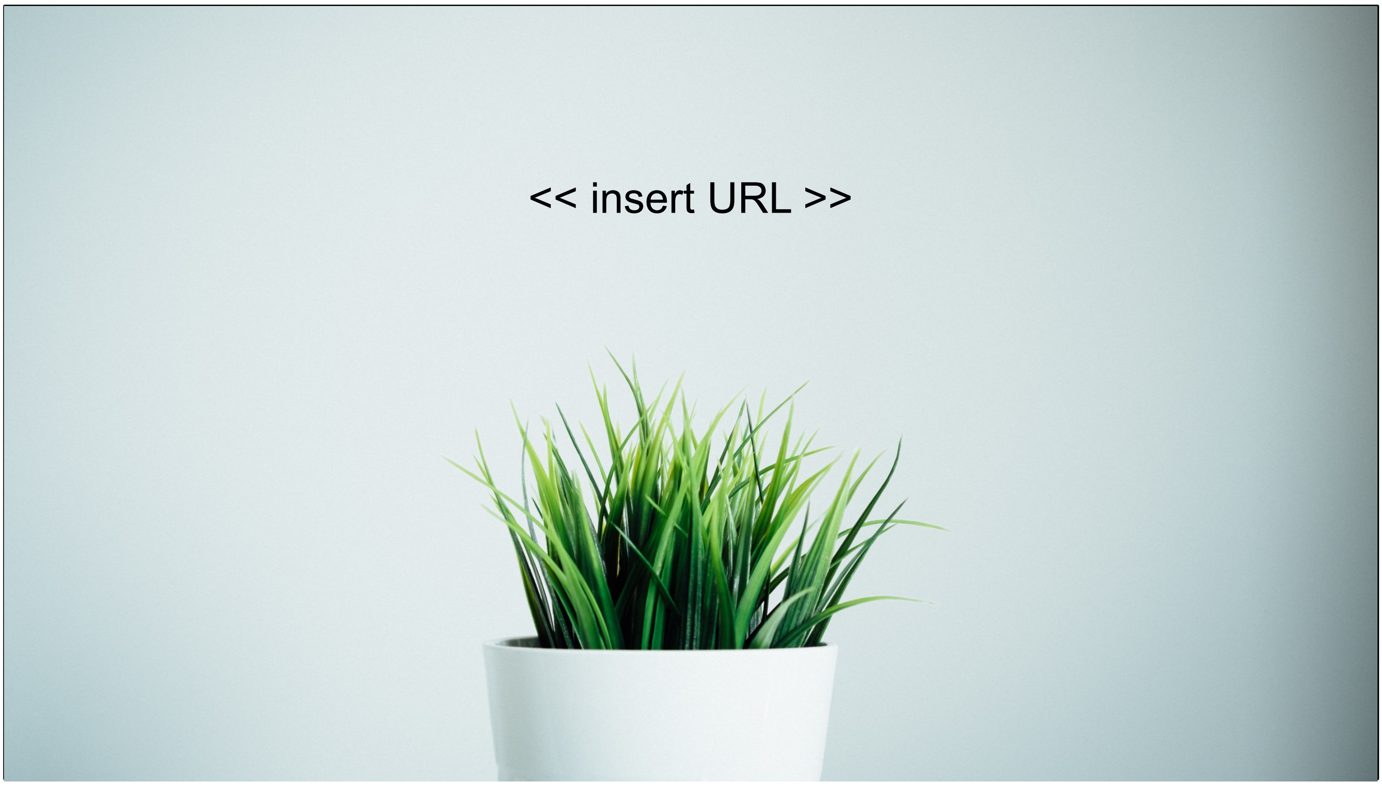

Before we begin, I would like to invite your assistance in our efforts to make our training package for GAS as good as possible.

We wish to evaluate the effectiveness of this training in preparing attendees to administer the GAS to research participants.

There are two short surveys designed to take no more than five minutes. One to be completed now, and one to be completed at a later date when we next meet up.

I will copy and paste the link into the "chat" function, and also have it displayed here.

It is voluntary, and all personal details will be redacted by an independent staffer from AKTN not involved with this study.

I will give you five minutes now to complete the survey if you wish.

____

(Whist allowing five minutes, prepare for the breakout session – and "allocate" the breakout groups for later)

Objectives

- Introduce **Goal Attainment Scaling** (GAS)
- Explain the **importance and benefits of goal setting**
- Discuss **SMART goals**
- Overview the **GAS template**
- Describe how to **set and scale a goal** for GAS
- Suggest **how to conduct meetings** with patients
- Allow an opportunity for **practical simulation**
- Offer tips on **troubleshooting common challenges**
- Describe the **review and scoring process**

5

My objectives for the training are (read from slide; **reveal line by line**)

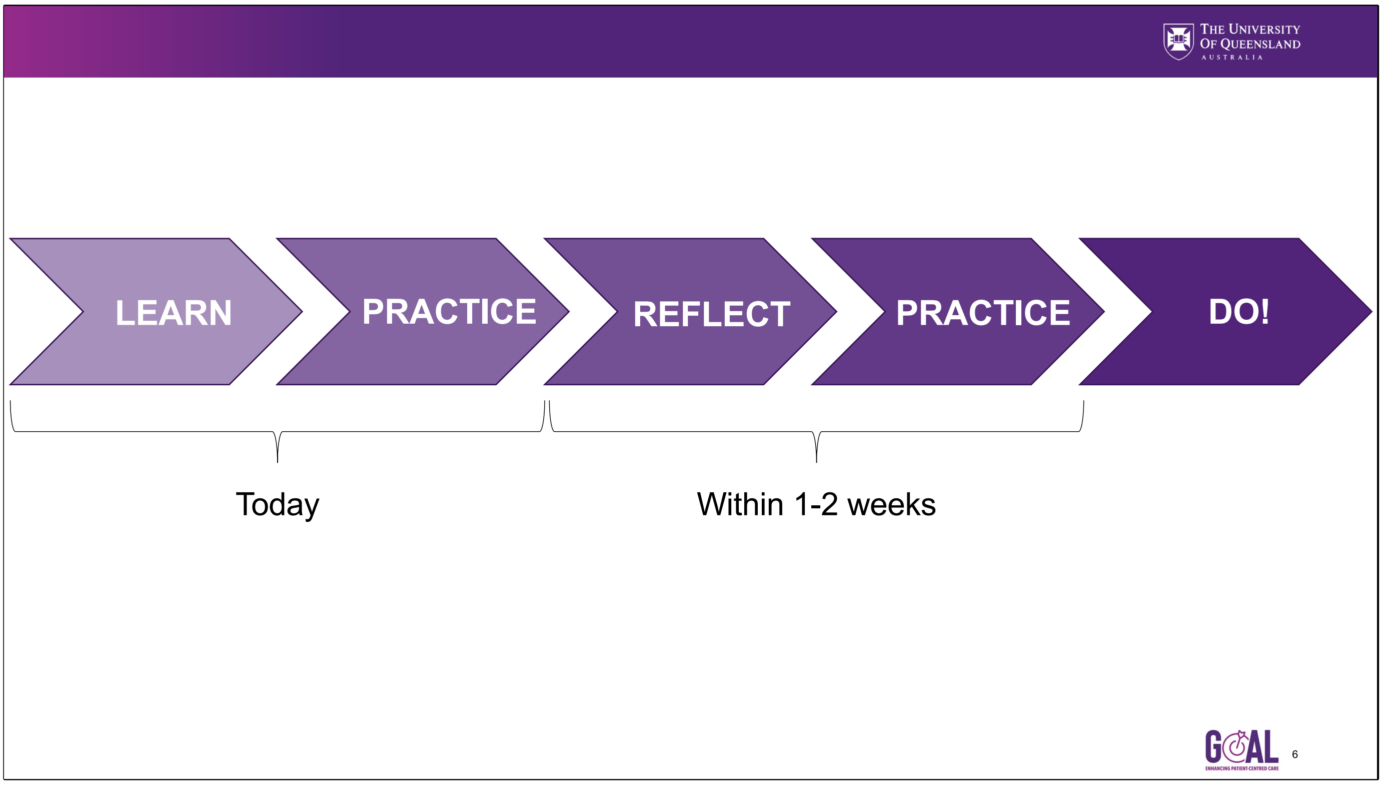

More graphically, our training is structured as follows:

- Learn (learn the theory and the processes)
- Practice (simulation exercises in breakout groups today)
  (Both of these first things will occur today)
- Reflect (period of reflection to further digest today's material, and have the opportunity to read through the training manual and note any questions)
- Practice (a further practical simulation one-on-one with myself or Bonnie; this also allows for answering any questions you have)
  (These two things will occur in the coming fortnight, pending our mutual availability)
- Do (you will be ready to go live and see patients)

There is a limit as to how much we can "teach" you today, hence a degree of self-directed reading and learning is required.

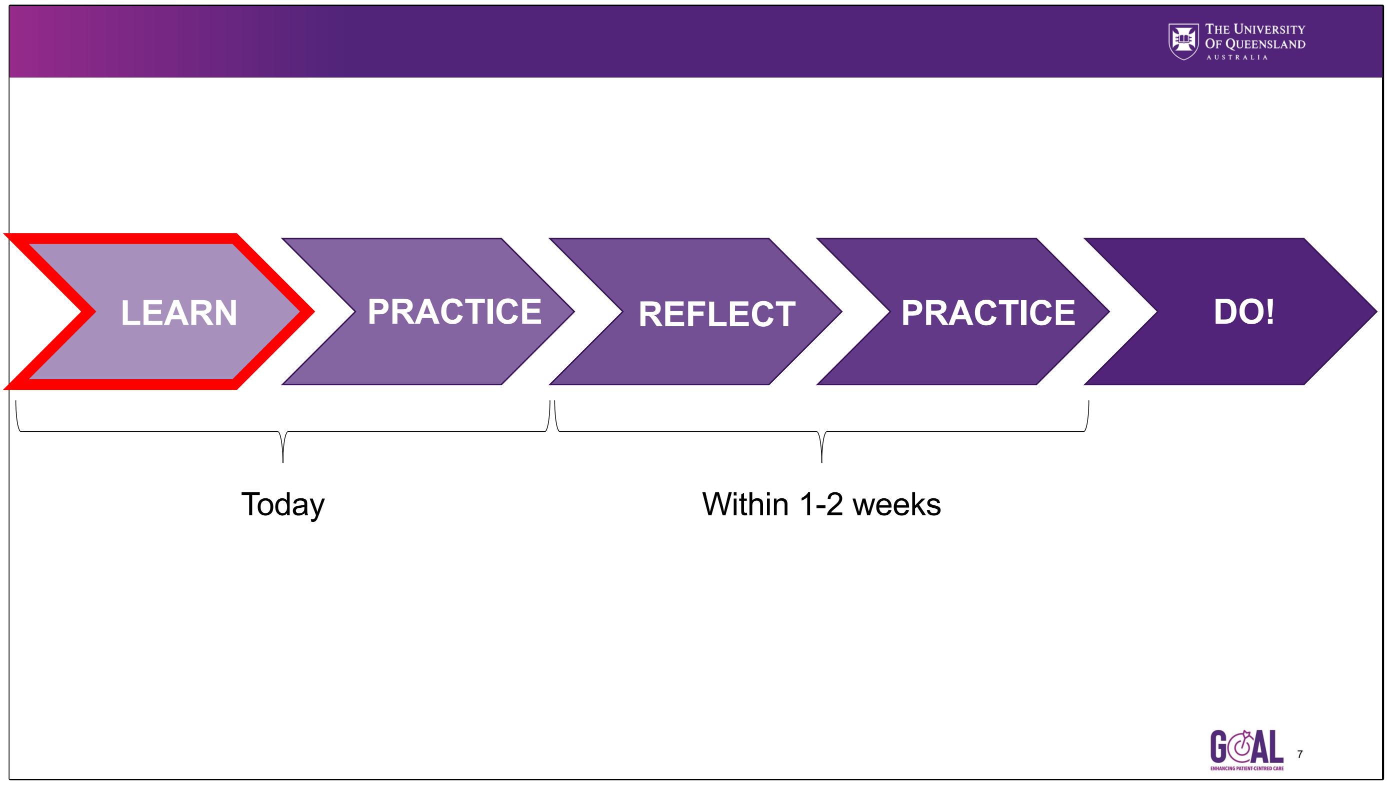

We will now begin with the theory components of our training

Objectives

- Introduce **Goal Attainment Scaling** (GAS)
- Explain the **importance and benefits of goal setting**
- Discuss **SMART goals**
- Overview the **GAS template**
- Describe how to **set and scale a goal** for GAS
- Suggest **how to conduct meetings** with patients
- Allow an opportunity for **practical simulation**
- Offer tips on **troubleshooting common challenges**
- Describe the **review and scoring process**

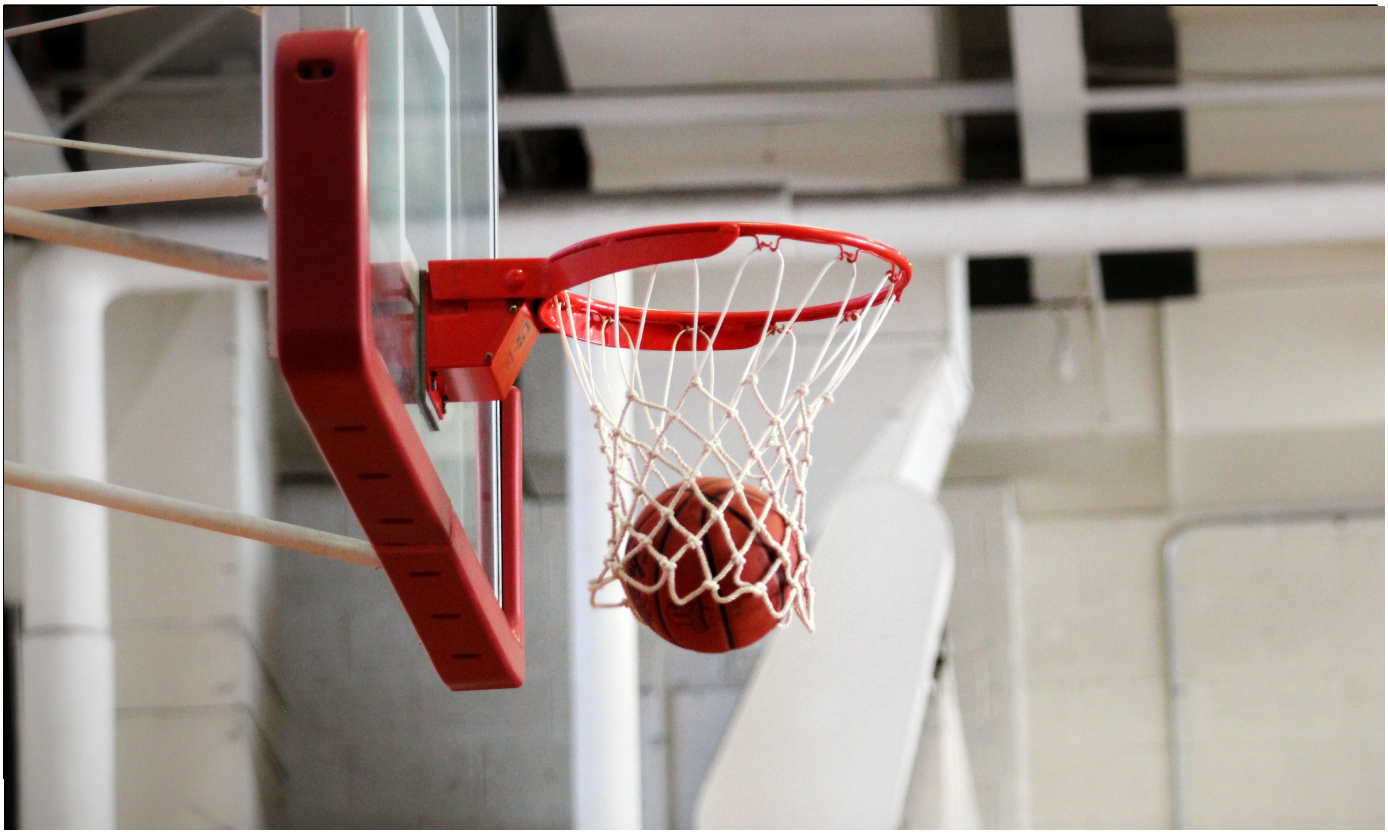

GAS is a tool which documents a patient's goal, and then scores the extent to which they are attained.

Goals are unique to the patient and their situation.

The GAS process was first developed in the 1960s, and has been deployed in a wide range of research and clinical settings. It is most frequently used in geriatric and general rehabilitation medicine, but also in paediatrics, community health, pain medicine, mental health and haematology.

THE UNIVERSITY OF QUEENSLAND AUSTRALIA

Goal Attainment Scaling

Step	Detail
1	Identify a goal
2	Define the current state
3	Identify potentially better and worse outcomes
4	Weight the goals
5	At follow-up, compare the current status with baseline status

GOAL ENHANCING PATIENT-CENTRED CARE 10

The basic steps are

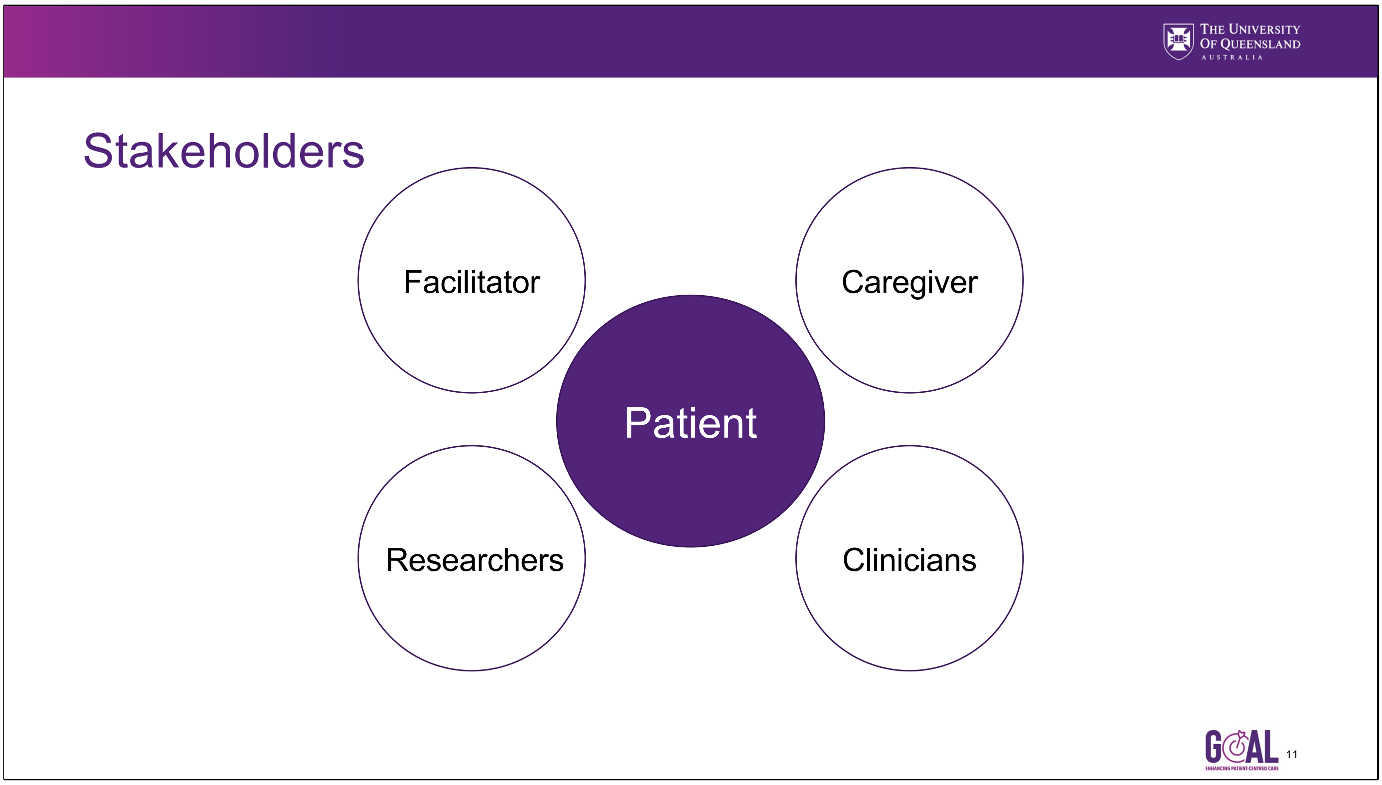

There are several stakeholders involved in the GAS process
REVEAL

- Patients – the most important stakeholder in this exercise
- Facilitator – that's you! Someone who partners with the patient through the process
- Caregivers – a possible valuable resource for collateral history and support (A support person is welcome to attend the GAS meetings at the patient's request/consent)
- Clinicians – important in clinical settings, but due to the trial's design they will not be involved in GAS for the GOAL Trial
- Researchers – that's me and the broader GOAL team; the output of the GAS process will be an outcome for the research

ANY QUESTIONS?

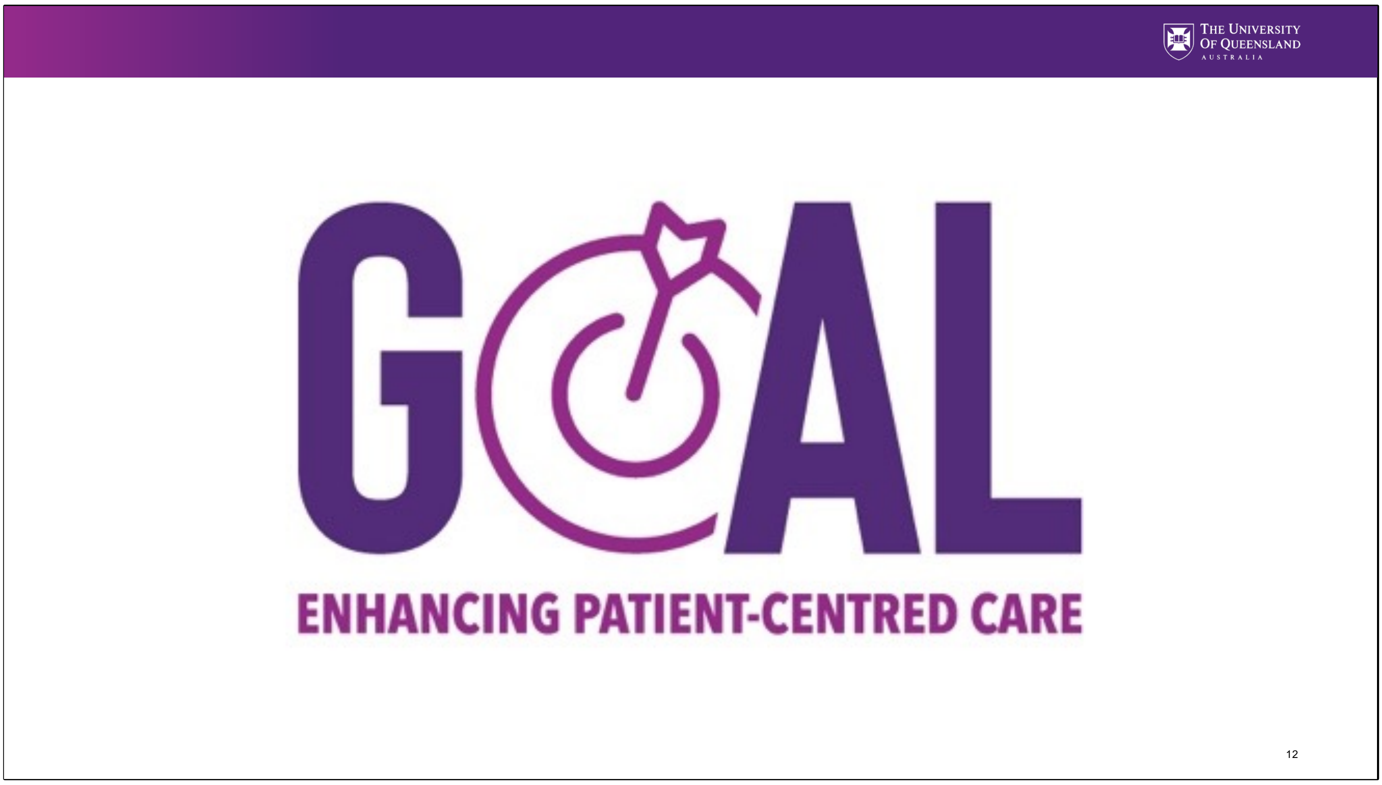

Speaking of research, I wanted to stress a particular point.

Significant time has been invested into supporting the effective implementation of the GAS process as it is the measure for the GOAL Trial's primary outcome.

Hence it is essential to be correctly done from the outset.

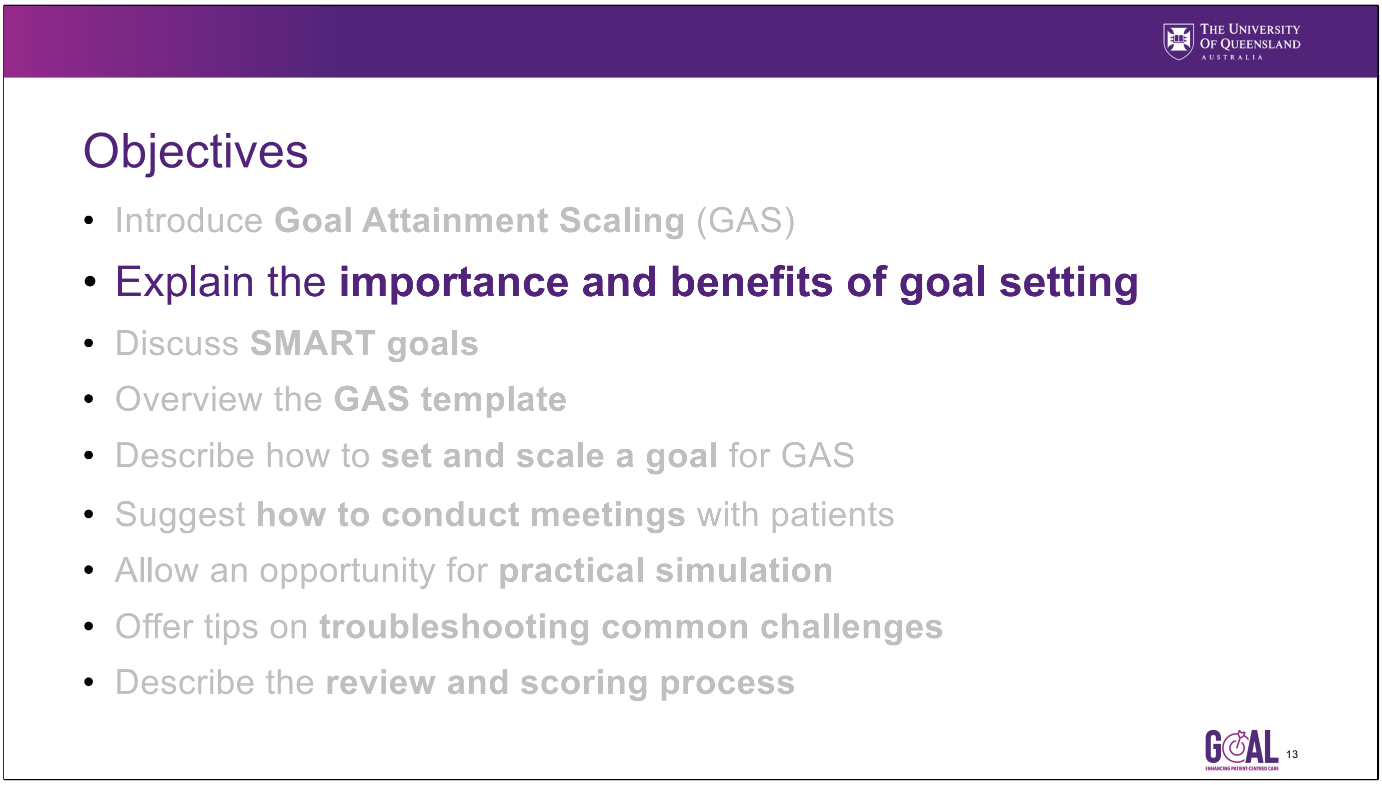
The University Of Queensland Australia
Objectives
Introduce Goal Attainment Scaling (GAS)
Explain the importance and benefits of goal setting
Discuss SMART goals
Overview the GAS template
Describe how to set and scale a goal for GAS
Suggest how to conduct meetings with patients
Allow an opportunity for practical simulation
Offer tips on troubleshooting common challenges
Describe the review and scoring process
GOAL
13

Benefits and importance of goal setting

- Focusing on individually-desired results
- Measures meaningful change
- Supports shared decision-making
- Unifies attention on individualised outcomes

14

Something you may have come across is the concept of patient-centred care.

It is something which is gaining prominence, and increasingly recognized as important for patients.

Patient-identified goals are needed to underpin patient-centred care.

The benefits of involving patient in their care, through goal setting, include:

REVEAL

- Focusing on individually-desired, rather than system-set results
- Measuring, and thus allowing praise of, meaningful changes achieved by a patient
- Providing a tool for collaboration and communication between patients and their healthcare profressional in the interests of shared decision-making
- Unifying attention on individualized outcomes rather than potentially disparate and conflicting disease-specific outcomes

We have sought to highlight the benefits of goal setting in a number of ways through the GAS process (specifically in the patient information sheet, and in the conversation starters) so that patients can be disposed to seeing GAS favourably from the outset.

ANY QUESTIONS?

Objectives

- Introduce **Goal Attainment Scaling** (GAS)
- Explain the **importance and benefits of goal setting**
- Discuss **SMART goals**
- Overview the **GAS template**
- Describe how to **set and scale a goal** for GAS
- Suggest **how to conduct meetings** with patients
- Allow an opportunity for **practical simulation**
- Offer tips on **troubleshooting common challenges**
- Describe the **review and scoring process**

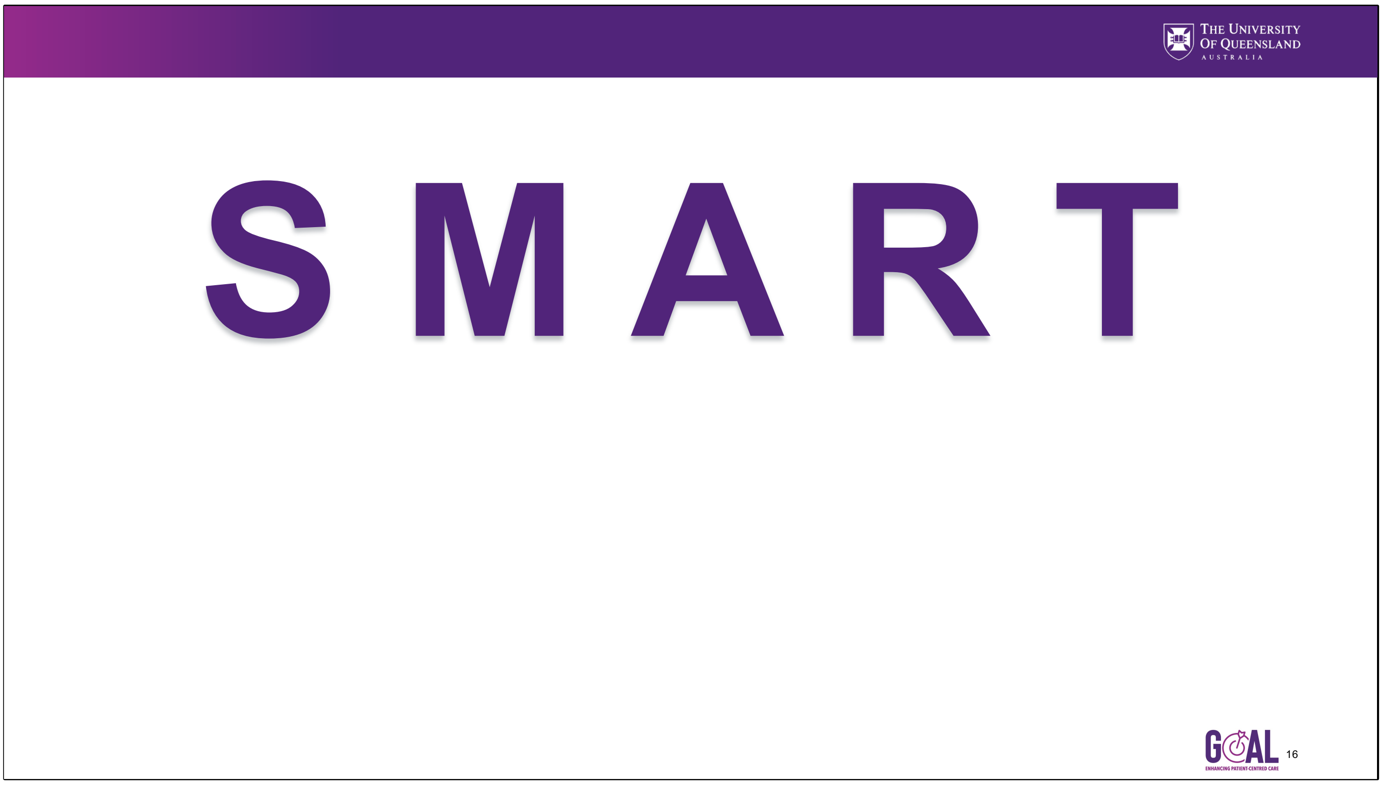

Goals should meet the SMART criteria

- Specific
- Measurable
- Achievable
- Relevant
- Timebound

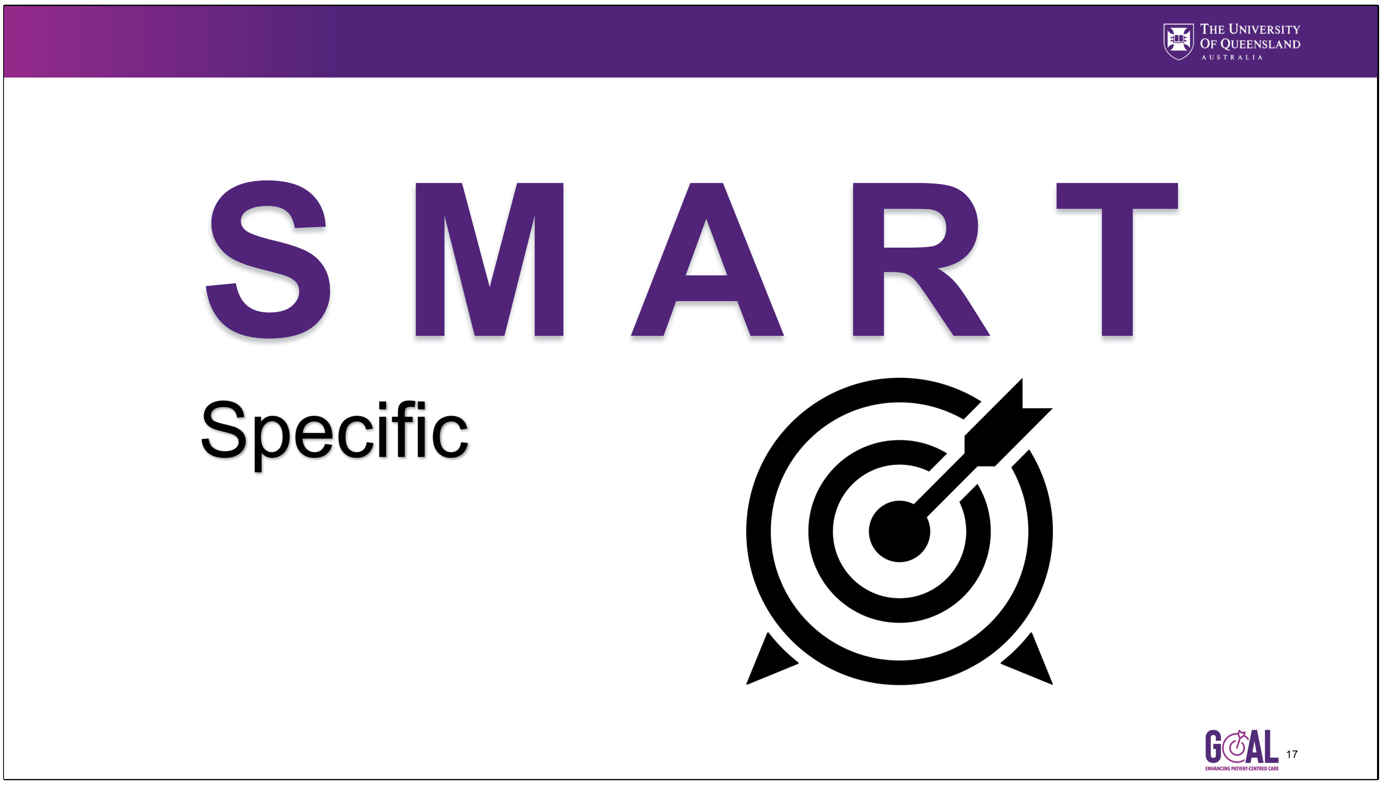

Goals should meet the SMART criteria

SPECIFIC

- Needs to be clear what the problem or desired change is being targeted
- If a goal is "to walk better" – does that mean: walk a longer distance, walk without an aid, or walk without any pain
- If all three are important, then separate them into three goals

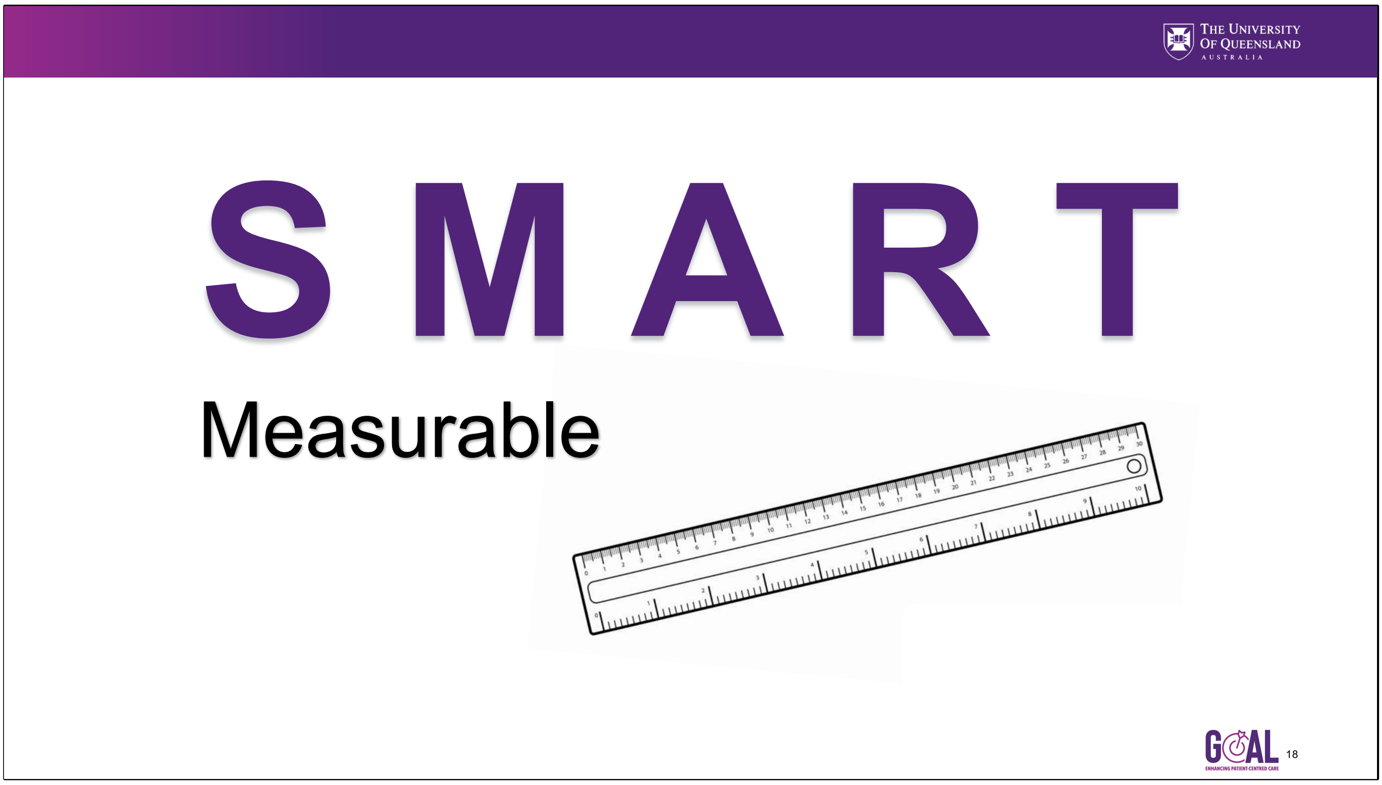

MEASURABLE

- Each goal needs an objective measure
- Objective measures are easy to determine where the goal can relate to a frequency (“cook dinner three nights a week”) or distance (“cycle ten kilometres”).
- Sometimes a degree of subjectivity has to be accommodated. For example, whilst “have less pain” may be seen as subjective, it can be made more objective by reframing it as “reduce my pain levels from 8/10 with activity, to 4/10 with activity” or “use only one 5mg oxycodone a day”.

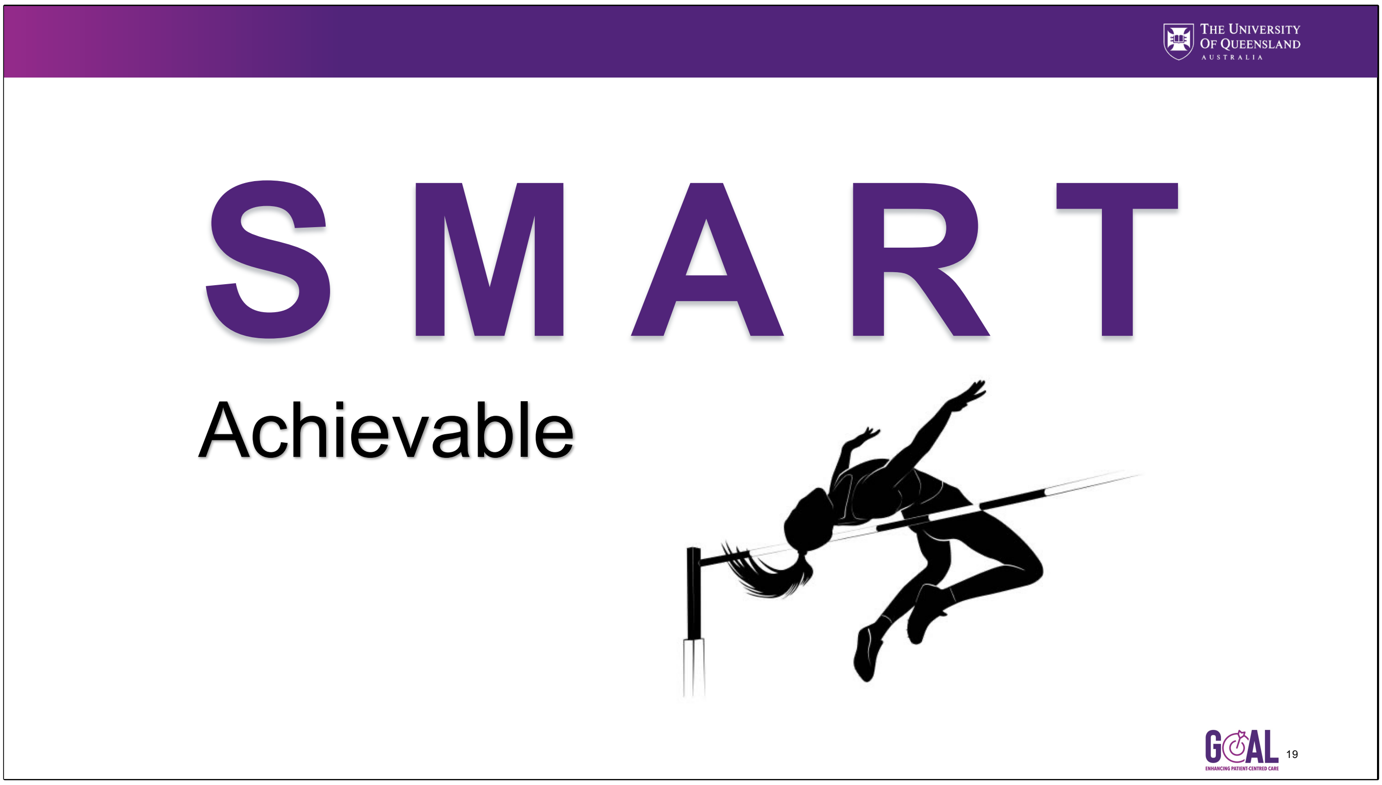

ACHIEVEABLE

- Patients, with prompting from their facilitator, will have to consider how achievable their desired goal is.
- Important considerations include reflecting on when they last were able to achieve that outcome and how far from being able to do it now.
- For example, if their goal is to walk 50 metres it would be important to probe when they last walked that far and to understand how far they comfortably walk now.

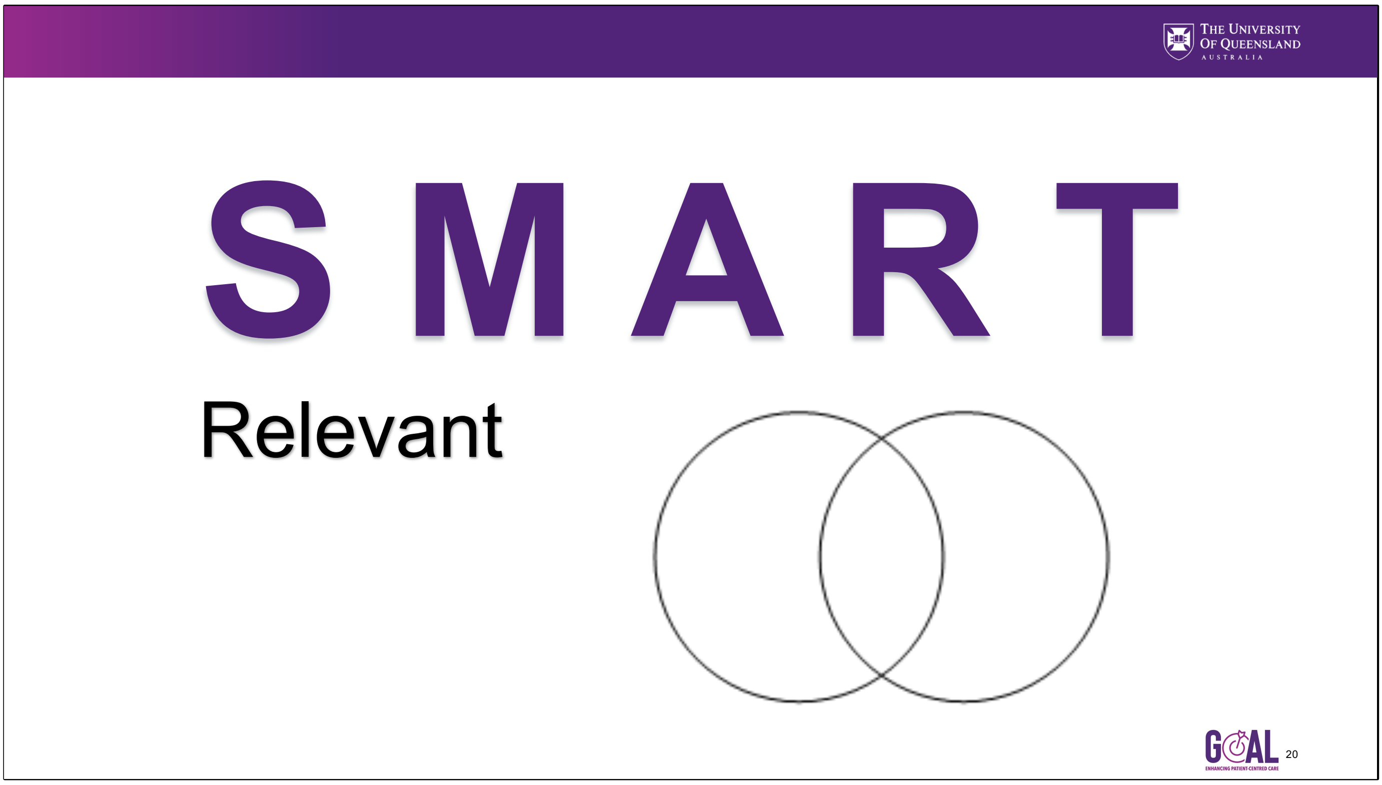

RELEVANT

- Goals should be of relevance to the patient, and given they are setting them they intuitively should be.
- It is not necessary for a carer or clinician to endorse its relevance.
- The facilitator may wish to prompt the patient to reflect on the benefit or purpose that attaining the goal would bring.

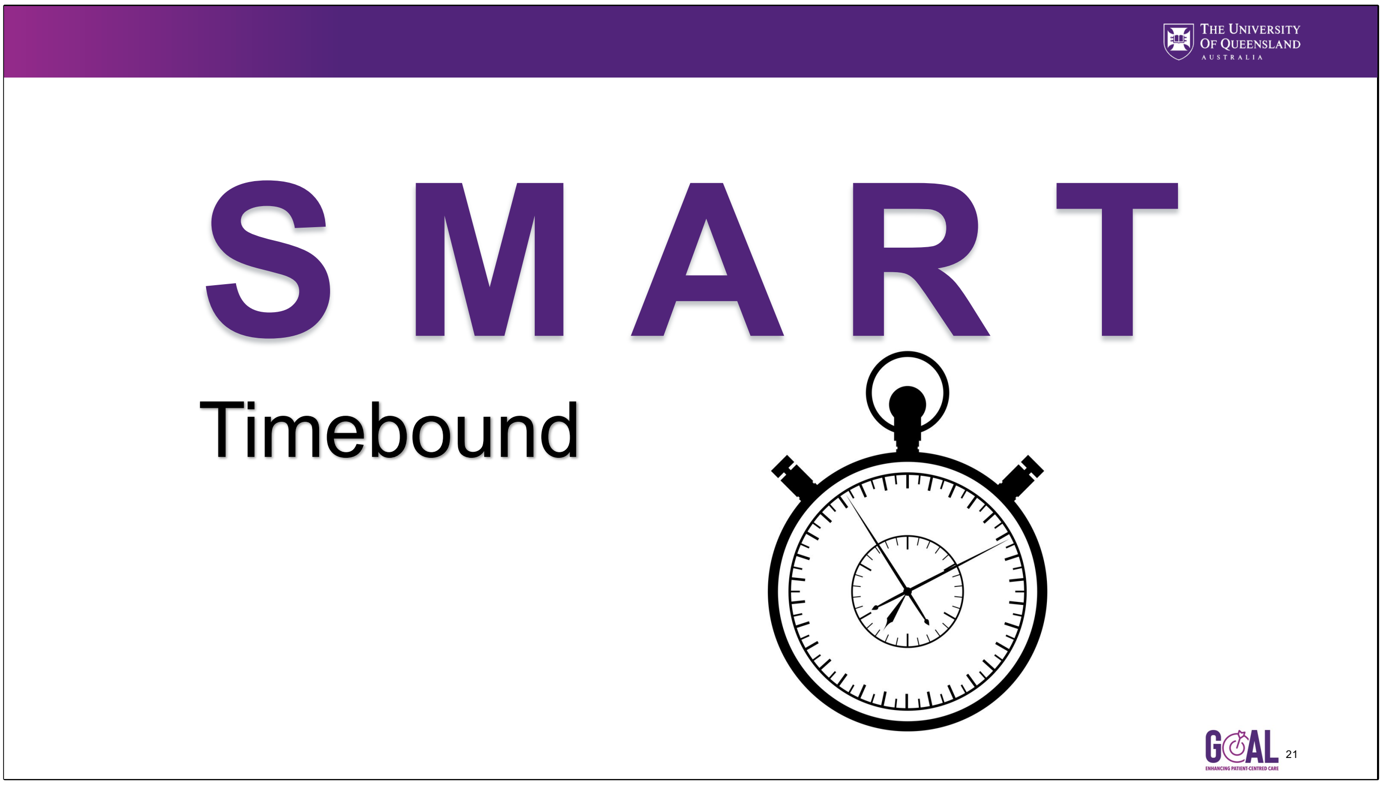

TIMEBOUND

- The date for achieving the goal needs to be stated. The patient should consider whether their goal is something which is likely to occur in the time period available.
- The date for achieving the goal is to be three months, in keeping with the trial's primary outcome measure.
- Please note you do not revise any goals at three months.

THE UNIVERSITY OF QUEENSLAND AUSTRALIA

Objectives

- Introduce **Goal Attainment Scaling** (GAS)
- Explain the **importance and benefits of goal setting**
- Discuss **SMART goals**
- Overview the **GAS template**
- Describe how to **set and scale a goal** for GAS
- Suggest **how to conduct meetings** with patients
- Allow an opportunity for **practical simulation**
- Offer tips on **troubleshooting common challenges**
- Describe the **review and scoring process**

GOAL ENHANCING PATIENT-CENTRED CARE 22

Goals for <insert name>

Trial ID: __________

Date set: __________
Review date: __________

Timepoint: ☐ Baseline ☐ 3 months ☐ 6 months ☐ 12 months

**Domain**					
**Goal**					
**Much less than expected (-2)**					
**Somewhat less than expected (-1)**					
**Expected level (0)**					
**Somewhat better than expected (+1)**					
**Much better than expected (+2)**					
**Importance (1-3)**					
**Difficulty (1-3)**					

# indicates baseline performance *(Scale for importance and difficulty: 1 = fairly; 2 = very; 3 = extremely.)*

This is the template to be used. If you open Appendix A on page 26 you can see it there too.

For the GOAL Trial, it will be ideally printed and worked through as a paper-based version. Where required though, it can be shared on a screen in a Zoom call.

It is best to explain to the patient at the outset that this template is a useful way to conceptualize the GAS tool (i.e. gives them context as to the scaling process)

Let’s now work through the various elements of it to orient you to it….

Goals for Joe Hammell

Trial ID: 3234

Date set: 2 February 2021
Review date: 2 May 2021

Timepoint: ■ Baseline ☐ 3 months ☐ 6 months ☐ 12 months

**Domain**	Mobility	Community engagement			
**Goal**	To increase my endurance so I can walk further	To leave home more frequently so as to have more social interactions			
**Much less than expected (-2)**	I can walk 9 metres or less	I leave the house once a fortnight or less			
**Somewhat less than expected (-1)**	I can walk 10-19 metres (Current: 10 metres #)	I leave the house twice or three times a fortnight (Current: twice #)			
**Expected level (0)**	I can walk 20-29 metres	I leave the house four or five times a fortnight			
**Somewhat better than expected (+1)**	I can walk 30-39 metres	I leave the house six times a fortnight			
**Much better than expected (+2)**	I can walk 40 metres or more	I leave the house seven or more times a fortnight			
**Importance (1-3)**	2	3			
**Difficulty (1-3)**	2	1			

# indicates baseline performance *(Scale for importance and difficulty: 1 = fairly; 2 = very; 3 = extremely.)* 24

We will use a populated template to help with the orientation being more easily understood.

This is Appendix B on page 28.

Goals for Joe Hammell

Trial ID: 3234

Date set: 2 February 2021
Review date: 2 May 2021

Timepoint: ■ Baseline ☐ 3 months ☐ 6 months ☐ 12 months

**Domain**	Mobility	Community engagement			
**Goal**	To increase my endurance so I can walk further	To leave home more frequently so as to have more social interactions			
**Much less than expected**<br><br>**(-2)**	I can walk 9 metres or less	I leave the house once a fortnight or less			
**Somewhat less than expected**<br><br>**(-1)**	I can walk 10-19 metres (Current: 10 metres #)	I leave the house twice or three times a fortnight (Current: twice #)			
**Expected level**<br><br>**(0)**	I can walk 20-29 metres	I leave the house four or five times a fortnight			
**Somewhat better than expected**<br><br>**(+1)**	I can walk 30-39 metres	I leave the house six times a fortnight			
**Much better than expected**<br><br>**(+2)**	I can walk 40 metres or more	I leave the house seven or more times a fortnight			
**Importance (1-3)**	2	3			
**Difficulty (1-3)**	2	1			

# indicates baseline performance *(Scale for importance and difficulty: 1 = fairly; 2 = very; 3 = extremely.)* 25

At the top, there is space to note the patient's name as well as the day's date.
The review date should be noted as three months from the date the goals are set.

Goals for Joe Hammell

Trial ID: 3234

Date set: 2 February 2021
Review date: 2 May 2021

Timepoint: ■ Baseline ☐ 3 months ☐ 6 months ☐ 12 months

**Domain**	Mobility	Community engagement			
**Goal**	To increase my endurance so I can walk further	To leave home more frequently so as to have more social interactions			
**Much less than expected (-2)**	I can walk 9 metres or less	I leave the house once a fortnight or less			
**Somewhat less than expected (-1)**	I can walk 10-19 metres (Current: 10 metres #)	I leave the house twice or three times a fortnight (Current: twice #)			
**Expected level (0)**	I can walk 20-29 metres	I leave the house four or five times a fortnight			
**Somewhat better than expected (+1)**	I can walk 30-39 metres	I leave the house six times a fortnight			
**Much better than expected (+2)**	I can walk 40 metres or more	I leave the house seven or more times a fortnight			
**Importance (1-3)**	2	3			
**Difficulty (1-3)**	2	1			

# indicates baseline performance *(Scale for importance and difficulty: 1 = fairly; 2 = very; 3 = extremely.)* 26

This is largely redundant for the GOAL Trial, but you would indicate it is the baseline meeting

Goals for Joe Hammell Trial ID: 3234

Date set: 2 February 2021
Review date: 2 May 2021

Timepoint: ■ Baseline ☐ 3 months ☐ 6 months ☐ 12 months

Domain	Mobility	Community engagement			
**Goal**	To increase my endurance so I can walk further	To leave home more frequently so as to have more social interactions			
**Much less than expected (-2)**	I can walk 9 metres or less	I leave the house once a fortnight or less			
**Somewhat less than expected (-1)**	I can walk 10-19 metres (Current: 10 metres #)	I leave the house twice or three times a fortnight (Current: twice #)			
**Expected level (0)**	I can walk 20-29 metres	I leave the house four or five times a fortnight			
**Somewhat better than expected (+1)**	I can walk 30-39 metres	I leave the house six times a fortnight			
**Much better than expected (+2)**	I can walk 40 metres or more	I leave the house seven or more times a fortnight			
**Importance (1-3)**	2	3			
**Difficulty (1-3)**	2	1			

# indicates baseline performance *(Scale for importance and difficulty: 1 = fairly; 2 = very; 3 = extremely.)* 27

The goal is written in plain language

<u>Goals for Joe Hammell</u> Trial ID: 3234

Date set: 2 February 2021
Review date: 2 May 2021

Timepoint: ■ Baseline ☐ 3 months ☐ 6 months ☐ 12 months

**Domain**	Mobility	Community engagement			
**Goal**	To increase my endurance so I can walk further	To leave home more frequently so as to have more social interactions			
**Much less than expected (-2)**	I can walk 9 metres or less	I leave the house once a fortnight or less			
**Somewhat less than expected (-1)**	I can walk 10-19 metres (Current: 10 metres #)	I leave the house twice or three times a fortnight (Current: twice #)			
**Expected level (0)**	I can walk 20-29 metres	I leave the house four or five times a fortnight			
**Somewhat better than expected (+1)**	I can walk 30-39 metres	I leave the house six times a fortnight			
**Much better than expected (+2)**	I can walk 40 metres or more	I leave the house seven or more times a fortnight			
**Importance (1-3)**	2	3			
**Difficulty (1-3)**	2	1			

# indicates baseline performance *(Scale for importance and difficulty: 1 = fairly; 2 = very; 3 = extremely.)* 28

The goal domain which best matches the goal should be listed

We will discuss more about the goal domains shortly

Goals for Joe Hammell — Trial ID: 3234

Date set: 2 February 2021
Review date: 2 May 2021

Timepoint: ■ Baseline ☐ 3 months ☐ 6 months ☐ 12 months

**Domain**	Mobility	Community engagement			
**Goal**	To increase my endurance so I can walk further	To leave home more frequently so as to have more social interactions			
**Much less than expected (-2)**	I can walk 9 metres or less	I leave the house once a fortnight or less			
**Somewhat less than expected (-1)**	I can walk 10-19 metres (Current: 10 metres #)	I leave the house twice or three times a fortnight (Current: twice #)			
**Expected level (0)**	I can walk 20-29 metres	I leave the house four or five times a fortnight			
**Somewhat better than expected (+1)**	I can walk 30-39 metres	I leave the house six times a fortnight			
**Much better than expected (+2)**	I can walk 40 metres or more	I leave the house seven or more times a fortnight			
**Importance (1-3)**	2	3			
**Difficulty (1-3)**	2	1			

# indicates baseline performance *(Scale for importance and difficulty: 1 = fairly; 2 = very; 3 = extremely.)* 29

The bulk of the table provides space, as shown below, for the baseline and attainment measures to be articulated in detail.

The measures correspond to a numerical score as follows:

- Much less than expected: -2
- Somewhat less than expected: -1
- Expected level: 0
- Somewhat better than expected: +1
- Much better than expected: +2

Goals for Joe Hammell

Trial ID: 3234

Date set: 2 February 2021
Review date: 2 May 2021

Timepoint: ■ Baseline ☐ 3 months ☐ 6 months ☐ 12 months

**Domain**	Mobility	Community engagement			
**Goal**	To increase my endurance so I can walk further	To leave home more frequently so as to have more social interactions			
**Much less than expected** **(-2)**	I can walk 9 metres or less	I leave the house once a fortnight or less			
**Somewhat less than expected** **(-1)**	I can walk 10-19 metres (Current: 10 metres #)	I leave the house twice or three times a fortnight (Current: twice #)			
**Expected level** **(0)**	I can walk 20-29 metres	I leave the house four or five times a fortnight			
**Somewhat better than expected** **(+1)**	I can walk 30-39 metres	I leave the house six times a fortnight			
**Much better than expected** **(+2)**	I can walk 40 metres or more	I leave the house seven or more times a fortnight			
**Importance (1-3)**	2	3			
**Difficulty (1-3)**	2	1			

# indicates baseline performance

(Scale for importance and difficulty: 1 = fairly; 2 = very; 3 = extremely.)

The bottom part of the table prompts for a weighting to be recorded with respects to each goal’s importance and perceived difficulty according to the patient.

The weighting ranges from 1 for a “fairly” important or difficult goal, through to 3 for an “extremely” important or difficult one, and should be determined by the patient.

Goals for Joe Hammell

Trial ID: 3234

Date set: 2 February 2021
Review date: 2 May 2021

Timepoint: ■ Baseline ☐ 3 months ☐ 6 months ☐ 12 months

Domain	Mobility	Community engagement			
**Goal**	To increase my endurance so I can walk further	To leave home more frequently so as to have more social interactions			
**Much less than expected (-2)**	I can walk 9 metres or less	I leave the house once a fortnight or less			
**Somewhat less than expected (-1)**	I can walk 10-19 metres (Current: 10 metres #)	I leave the house twice or three times a fortnight (Current: twice #)			
**Expected level (0)**	I can walk 20-29 metres	I leave the house four or five times a fortnight			
**Somewhat better than expected (+1)**	I can walk 30-39 metres	I leave the house six times a fortnight			
**Much better than expected (+2)**	I can walk 40 metres or more	I leave the house seven or more times a fortnight			
**Importance (1-3)**	2	3			
**Difficulty (1-3)**	2	1			

# indicates baseline performance

(Scale for importance and difficulty: 1 = fairly; 2 = very; 3 = extremely.)

31

Remember to place a # where the baseline performance is
Where there is a range, in the case of a continuous variable being used, then write the baseline out separately

ANY QUESTIONS?

Objectives

- Introduce **Goal Attainment Scaling** (GAS)
- Explain the **importance and benefits of goal setting**
- Discuss **SMART goals**
- Overview the **GAS template**
- Describe how to **set and scale a goal** for GAS
- Suggest **how to conduct meetings** with patients
- Allow an opportunity for **practical simulation**
- Offer tips on **troubleshooting common challenges**
- Describe the **review and scoring process**

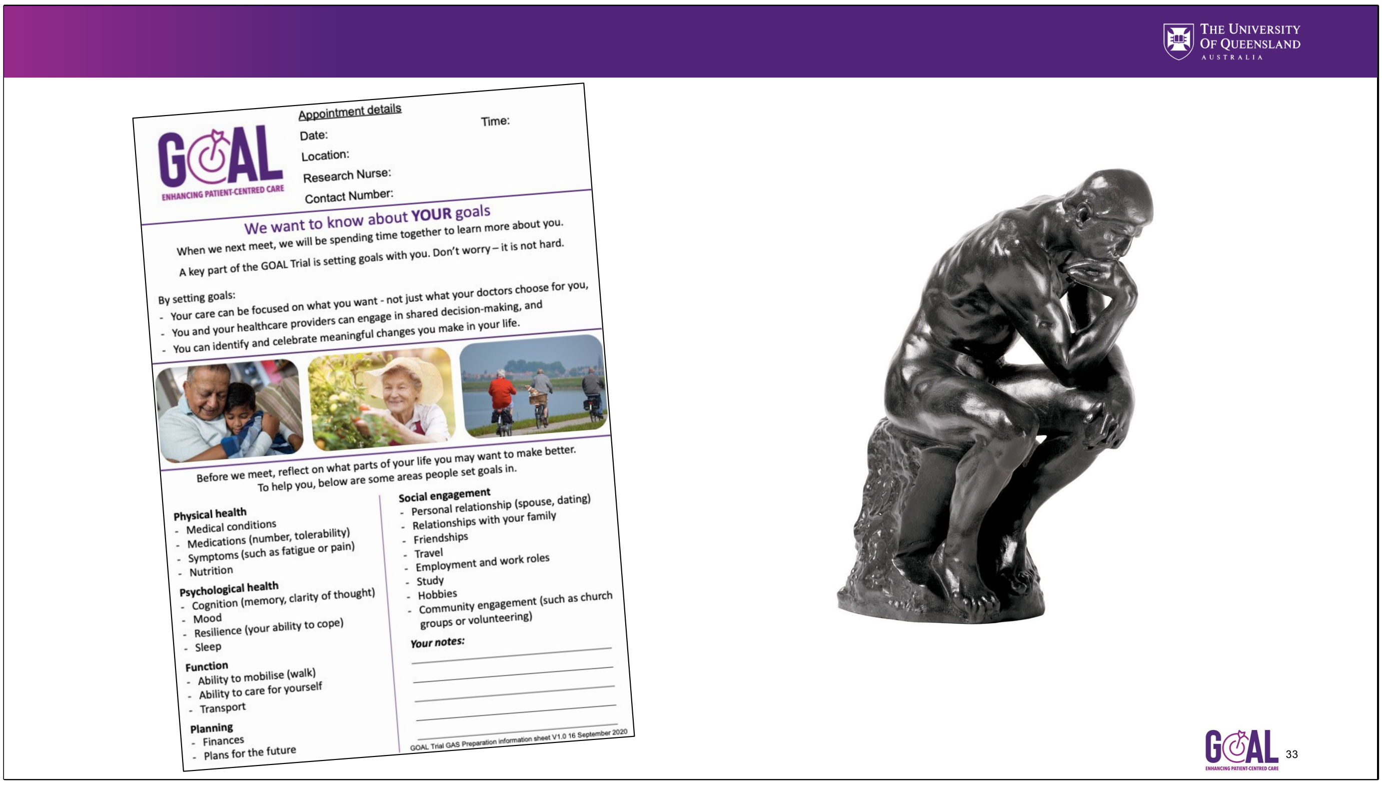

Self-reflection is important for setting goals that are relevant to the patient.

To prompt this desired self-reflection, a preparation information sheet on goal setting will be issued prior to the meeting.

The information sheet is shown on the screen, but please see Appendix C on page 30 of the training manual.

For the GOAL Trial, this preparation information sheet will be issued after a patient has been consented and confirmed as being eligible for the trial.

The intent for the information sheet, along with how it is introduced, is to leave the patient in a positive mindset as they approach the goal-setting meeting, and to give them adequate time to think about what they may like to achieve.

Goal domains

Physical health
- Medical conditions
- Medications (number, tolerability)
- Symptoms (such as fatigue or pain)
- Nutrition

Psychological health
- Cognition (memory, clarity of thought)
- Mood
- Resilience (your ability to cope)
- Sleep

Function
- Ability to mobilise (walk)
- Ability to care for yourself
- Transport

Planning
- Finances
- Plans for the future

Social engagement
- Personal relationship (spouse, dating)
- Relationships with your family
- Friendships
- Travel
- Employment and work roles
- Study
- Hobbies
- Community engagement (such as church groups or volunteering)

34

Whilst you can read over the patient information sheet in your own time, I wanted to highlight the goal domains.

They are useful to help identify areas in which they may wish to consider setting goals.

For data collection, in REDCap, you simply just need to pick one of the five broad categories: physical health, psychological health, function, planning, social engagement.

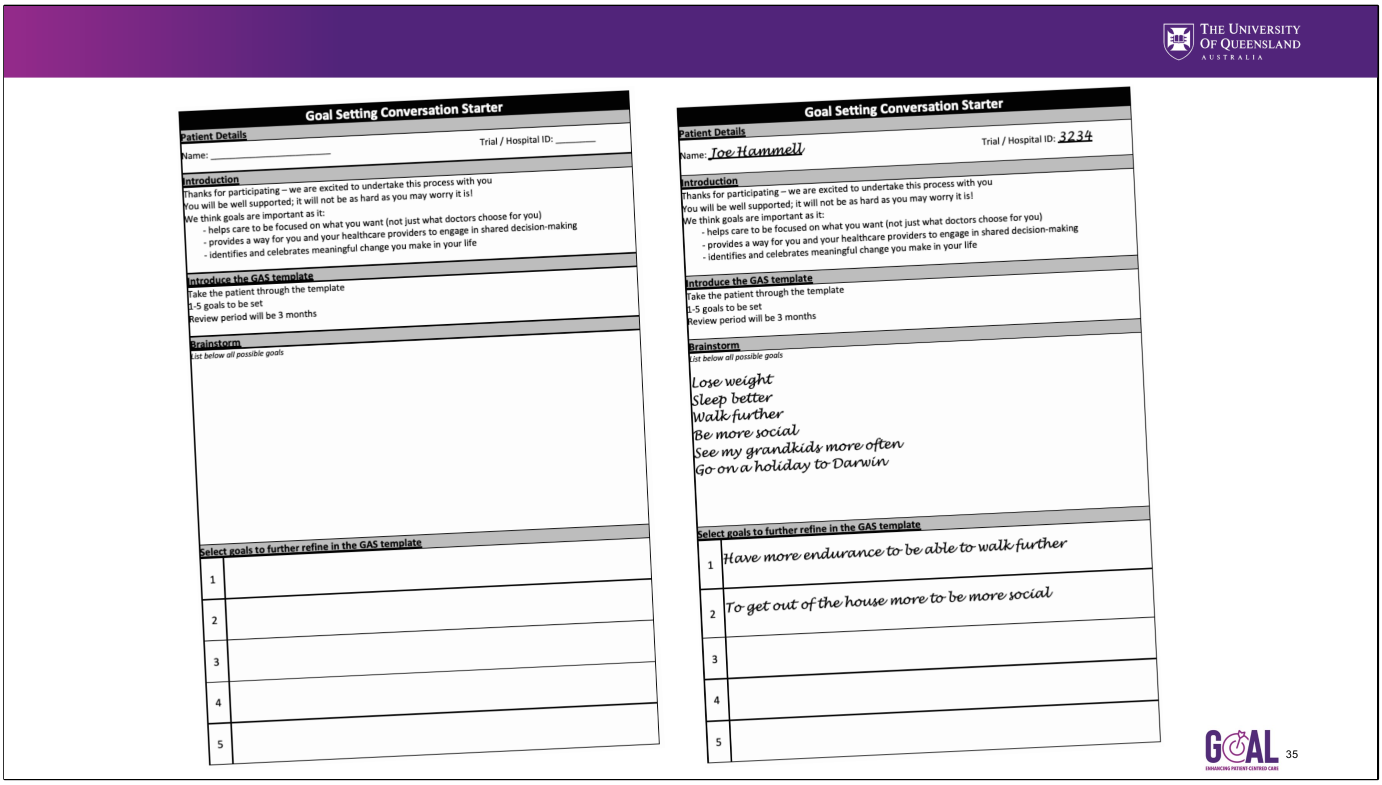

We will now turn our attention to Appendix D and E which is from page 32 in the training manual.

This is a conversation starter, and a great tool for you to get the patient talking about what they may wish to set a goal in.

Brainstorming ideas may be easy for some, but for other patients it may take more prompting and support.

There should be no judgement or moderation attempts made at this initial stage. Write down everything they say no matter how unworkable it may seem.

Refer back to the goal domains on the GAS preparation information sheet if the patient gets stuck.

The training manual offers some other prompting questions as well.

After the brainstorming is completed, move on to listing 1-5 goals to be set.

ANY QUESTIONS?

Set and scale a goal

Domain	Mobility
Goal	To increase my endurance so I can walk further
**Much less than expected**<br><br>**(-2)**	I can walk 9 metres or less
**Somewhat less than expected**<br><br>**(-1)**	I can walk 10-19 metres (Current: 10 metres #)
**Expected level**<br><br>**(0)**	I can walk 20-29 metres
**Somewhat better than expected**<br><br>**(+1)**	I can walk 30-39 metres
**Much better than expected**<br><br>**(+2)**	I can walk 40 metres or more
**Importance (1-3)**	2
**Difficulty (1-3)**	2

- Describe a measure

36

Once you have identified one to five goals, it is time to formally set and scale them.
Before you put pen to paper on the GAS template you will need to ensure the goal has a SINGLE measure. For example if a patient wishes to "walk better", the facilitator will need to agree with the patient how that is best measured.

It may be:
- walking a longer distance (metres as a measure), or
- mobilise more independently (level of assistance as a measure)

There cannot be multi-faceted measures in the one goal (i.e. 'metres' and 'level of assistance'). Should the patient have two measures that are identified as important to them, then two separate goals should be set.

Also key to describing a measure, is ensuring it can be **scaled to five different intervals**.

For continuous measures, such as distance, this is relatively straightforward.
Drawing on the above goal of "walking a longer distance", the five intervals could be scaled as: 80 metres; 60 metres; 40 metres; 20 metres; and 5 metres or less.

For continuous measures (such as distances), measures should be provided in ranges

For categorical measures this is more challenging but can often be achieved with some thought.
Drawing on the above example of "mobilise more independently", the five intervals could be scaled as: able to mobilise without any mobility aid; mobilise with a single-point stick; mobilise with a four-wheeled walker; mobilise with a wheelchair via a stand-pivot transfer; and, patient bed bound, requiring a hoist.

THE UNIVERSITY OF QUEENSLAND AUSTRALIA

Set and scale a goal

**Domain**	Mobility
**Goal**	To increase my endurance so I can walk further
**Much less than expected (-2)**	I can walk 9 metres or less
**Somewhat less than expected (-1)**	I can walk 10-19 metres (Current: 10 metres #)
**Expected level (0)**	I can walk 20-29 metres
**Somewhat better than expected (+1)**	I can walk 30-39 metres
**Much better than expected (+2)**	I can walk 40 metres or more
**Importance (1-3)**	2
**Difficulty (1-3)**	2

- Describe a measure
- Write the goal and domain

GOAL ENHANCING PATIENT CENTRED CARE 37

Once an appropriate measure is identified, the facilitator should now write the goal in the template in the “Goal” row.

The goal should be written in plain language. In this part of the form it is not necessary to write a SMART goal, as the elements of that are set out elsewhere (specifically, in the scaling outcomes and the setting of a review date).

At this time, you should also populate the “Domain” with the single most relevant option available.

Set and scale a goal

Domain	Mobility
Goal	To increase my endurance so I can walk further
Much less than expected (-2)	I can walk 9 metres or less
Somewhat less than expected (-1)	I can walk 10-19 metres (Current: 10 metres #)
Expected level (0)	I can walk 20-29 metres
Somewhat better than expected (+1)	I can walk 30-39 metres
Much better than expected (+2)	I can walk 40 metres or more
Importance (1-3)	2
Difficulty (1-3)	2

- Describe a measure
- Write the goal and domain
- Define baseline performance

Once the goal is written, have the patient articulate their current performance.

An accurate understanding of their baseline performance is needed so expected outcomes are appropriately set. It is important to appreciate what occurs most often, not what they do sometimes “on a good day”.

It is also key to understand the recency of their performance, and its trajectory. For example, is their ability to walk 20 metres something that has been stable for some months, or is it a new high or low point? It is also worth prompting the patient to share what they were able to do three months ago.

The baseline performance will be written in the “somewhat less than expected (-1)” row.

One exception to this is where there is no conceivable worse outcome than their current status. For example, if their broad goal is “to exercise more” and currently they do no exercise, then that would be written in the “much less than expected (-2)” row.

Another exception is where a patient has experienced a progressive decline in how they are performing in a particular aspect of their life. They may want focus on maintaining their current abilities and halt a deterioration. For example, a patient with kidney failure may have as their goal “to maintain a healthy body weight” in the setting of having lost one kilogram of weight for each of the past six months. For them it may be appropriate to set their baseline measure at the “expected level (0)” row.

Baseline performance is marked on the template by placing a hashtag (#) in the relevant box. Where continuous measures are used, and thus you need a range spelling out the actual current baseline as a discreet measure may be appropriate and reflected in the examples in this document.

THE UNIVERSITY OF QUEENSLAND AUSTRALIA

Set and scale a goal

**Domain**	Mobility
**Goal**	To increase my endurance so I can walk further
**Much less than expected (-2)**	I can walk 9 metres or less
**Somewhat less than expected (-1)**	I can walk 10-19 metres (Current: 10 metres #)
**Expected level (0)**	I can walk 20-29 metres
**Somewhat better than expected (+1)**	I can walk 30-39 metres
**Much better than expected (+2)**	I can walk 40 metres or more
**Importance (1-3)**	2
**Difficulty (1-3)**	2

- Describe a measure
- Write the goal and domain
- Define baseline performance
- Define worse outcomes

GOAL ENHANCING PATIENT CENTRED CARE 39

Once the baseline performance is defined, poorer performance outcomes can be articulated.

This will be populated into the “much less than expected” row, unless the baseline performance is at that level.

Set and scale a goal

Domain	Mobility
**Goal**	To increase my endurance so I can walk further
**Much less than expected (-2)**	I can walk 9 metres or less
**Somewhat less than expected (-1)**	I can walk 10-19 metres (Current: 10 metres #)
**Expected level (0)**	I can walk 20-29 metres
**Somewhat better than expected (+1)**	I can walk 30-39 metres
**Much better than expected (+2)**	I can walk 40 metres or more
**Importance (1-3)**	2
**Difficulty (1-3)**	2

- Describe a measure
- Write the goal and domain
- Define baseline performance
- Define worse outcomes
- Define the expected outcome

The most challenging part of the scaling process is working with the patient to describe expected performance. It is further complicated in situations where the goal relates to an area in which the facilitator may not be familiar. For example, setting goals to "walk further" or "walk with mobility aids" when the facilitator has no rehabilitation or physiotherapy exposure.

The complexities of this stage of the GAS can be addressed by:

- Understanding their current and prior performance levels, and the trajectory of this.
- Gently challenging the patient on what they see as being a stretch or comfortable for them.
- Seeking collateral history from any support person present at the goal setting session.

There is a fine balance in not being too lenient or too firm, particularly in research settings where the GAS is being used as an outcome measure.

For the GOAL Trial, no input is to be sought or accepted from the patient's treating medical and allied health team, as it risks contaminating the trial results.

THE UNIVERSITY OF QUEENSLAND AUSTRALIA

Set and scale a goal

**Domain**	Mobility
**Goal**	To increase my endurance so I can walk further
**Much less than expected (-2)**	I can walk 9 metres or less
**Somewhat less than expected (-1)**	I can walk 10-19 metres (Current: 10 metres #)
**Expected level (0)**	I can walk 20-29 metres
**Somewhat better than expected (+1)**	I can walk 30-39 metres
**Much better than expected (+2)**	I can walk 40 metres or more
**Importance (1-3)**	2
**Difficulty (1-3)**	2

- Describe a measure
- Write the goal and domain
- Define baseline performance
- Define worse outcomes
- Define the expected outcome
- Define better outcomes

GOAL ENHANCING PATIENT CENTRED CARE 41

Once the expected outcome is populated, attention can turn to defining what outcome would be “somewhat better” and “much better” than expected.

Appropriate intervals between the measures should be chosen to ensure the spread between “much less than expected (-2)” and “much better than expected (+2)” is relatively spaced.

THE UNIVERSITY OF QUEENSLAND AUSTRALIA

Set and scale a goal

Domain	Mobility
Goal	To increase my endurance so I can walk further
Much less than expected (-2)	I can walk 9 metres or less
Somewhat less than expected (-1)	I can walk 10-19 metres (Current: 10 metres #)
Expected level (0)	I can walk 20-29 metres
Somewhat better than expected (+1)	I can walk 30-39 metres
Much better than expected (+2)	I can walk 40 metres or more
Importance (1-3)	2
Difficulty (1-3)	2

- Describe a measure
- Write the goal and domain
- Define baseline performance
- Define worse outcomes
- Define the expected outcome
- Define better outcomes
- Set a review date

GOAL ENHANCING PATIENT CENTRED CARE 42

Whilst the exact date of review may vary slightly in the reality of arranging review appointments, the review date should be written as three months from the date goals are written down.

THE UNIVERSITY OF QUEENSLAND AUSTRALIA

Set and scale a goal

Domain	Mobility
**Goal**	To increase my endurance so I can walk further
**Much less than expected (-2)**	I can walk 9 metres or less
**Somewhat less than expected (-1)**	I can walk 10-19 metres (Current: 10 metres #)
**Expected level (0)**	I can walk 20-29 metres
**Somewhat better than expected (+1)**	I can walk 30-39 metres
**Much better than expected (+2)**	I can walk 40 metres or more
**Importance (1-3)**	2
**Difficulty (1-3)**	2

- Describe a measure
- Write the goal and domain
- Define baseline performance
- Define worse outcomes
- Define the expected outcome
- Define better outcomes
- Set a review date
- Nominate a weighting

GOAL ENHANCING PATIENT CENTRED CARE 43

The weighting for each goal should now be assigned. A score is required for difficulty and importance, as the patient perceives it. Both the difficulty and importance use the same scale:
1: Fairly important/difficult
2: Very important/difficult
3: Extremely important/difficult

ANY QUESTIONS?

THE UNIVERSITY OF QUEENSLAND AUSTRALIA

Action plan

- Smaller steps to aim for in achieving the ultimate goal
- Build potential changes into daily routines
- Consider informal and formal resources to access

GOAL ENHANCING PATIENT CENTRED CARE 44

Once a goal is formulated, setting out to achieve it is the next phase.

In order to make an action plan, a patient may want to consider (**REVEAL EACH POINT**):

- what smaller steps they can aim for as they work towards the ultimate achievement of a goal,
- how to build the potential changes needed into their daily routines, and
- the informal and formal resources they can utilise (be it family supports, free online learning, input from their General Practitioner, or accessing the services of a physiotherapist etc).

To ensure there is no contamination of measuring the intervention's effect, you will not spend time developing an action plan.

You can provide verbal guidance, but only by using the three talking points noted above.

We recognize this is challenging given your likely natural inclination to be helpful, but it is imperative for the integrity of the trial's results.

ANY QUESTIONS?

Objectives

- Introduce **Goal Attainment Scaling** (GAS)
- Explain the **importance and benefits of goal setting**
- Discuss **SMART goals**
- Overview the **GAS template**
- Describe how to **set and scale a goal** for GAS
- Suggest **how to conduct meetings** with patients
- Allow an opportunity for **practical simulation**
- Offer tips on **troubleshooting common challenges**
- Describe the **review and scoring process**

45

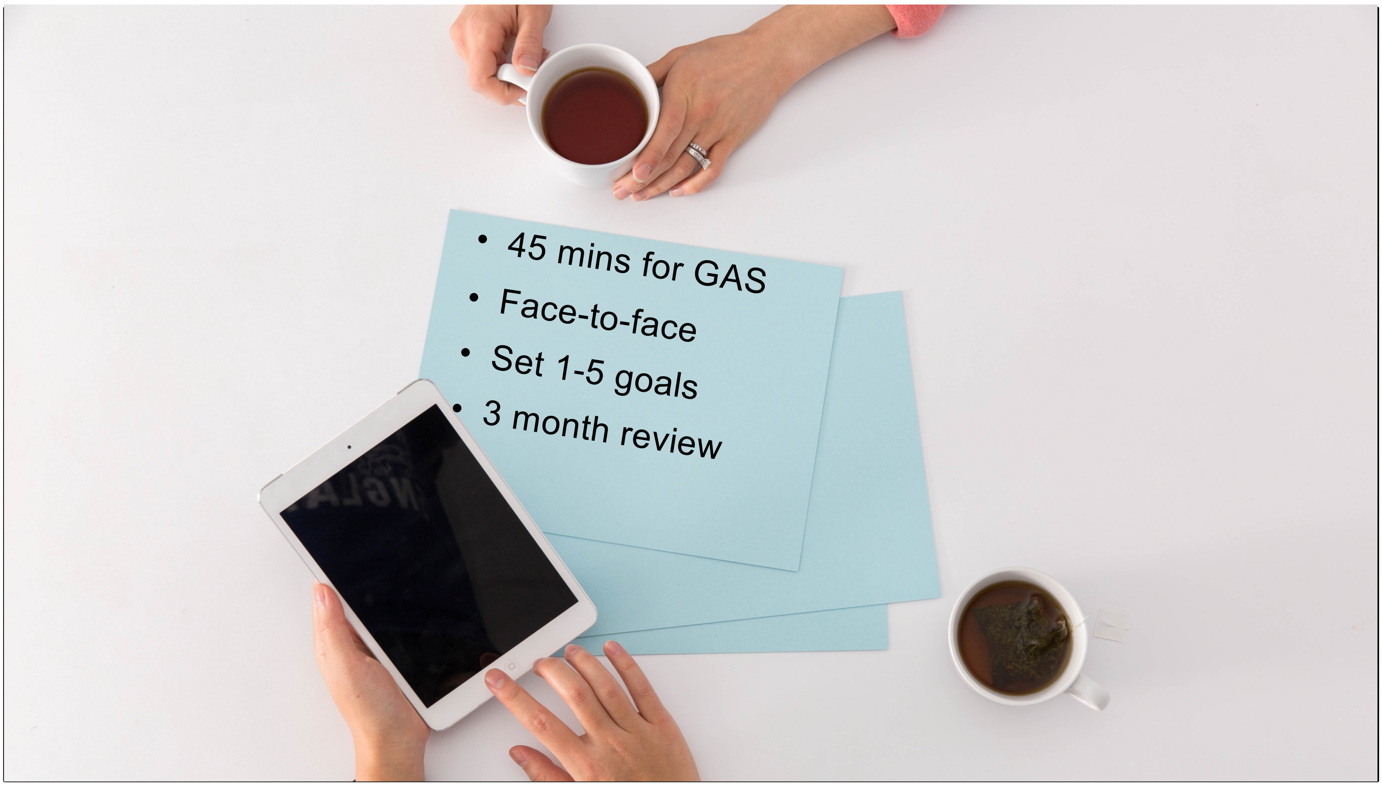

The GAS Training Manual sets out in detail the actions to take before, during and after the meeting.

Some of the key points (**REVEAL**):
- You should set aside 45 minutes for GAS – total of 75 mins
- Ideally have the meeting face-to-face
- Set 1-5 goals
- 3 month review

Conducting a goal setting meeting

- Strike the right tone
- Offer reassurance
- Reiterate the importance of goal setting
- Use the provided resources

After the meeting:

- Input into REDCap
- Redact all personal details prior to scanning
- Goals are **not** for distribution

47

A ”conversation guide” is built into the GAS Training Manual

In the meeting it is important to (**REVEAL**):

- Strike the right tone - share a sense of excitement for the session, but be yourself
- Offer reassurance they will be well supported through the process – there is a good structure being used
- Reiterate the importance of goal setting – using the points highlighted on the resources
- Use the provided resources – including the conversation starter handout, the GAS preparation sheet handout, and the talking points in the GAS Training Manual

If you are meeting virtually, Appendix F provides a combination of the brainstorming tool with the GAS template to save switching between multiple documents.

After the meeting (**REVEAL**):

- Input the data into REDCap AND scan the completed GAS Template into REDCap
- Must redact the form of all personal details before scanning it into REDCap as it risks breaching patient confidentiality otherwise
- The document will NOT be issued to either the patient, or their clinicians (including the Geriatrician, Nephrologist or GP)

Patient can write their own notes.
Patients can NOT take photos with their handheld devices.

ANY QUESTIONS?

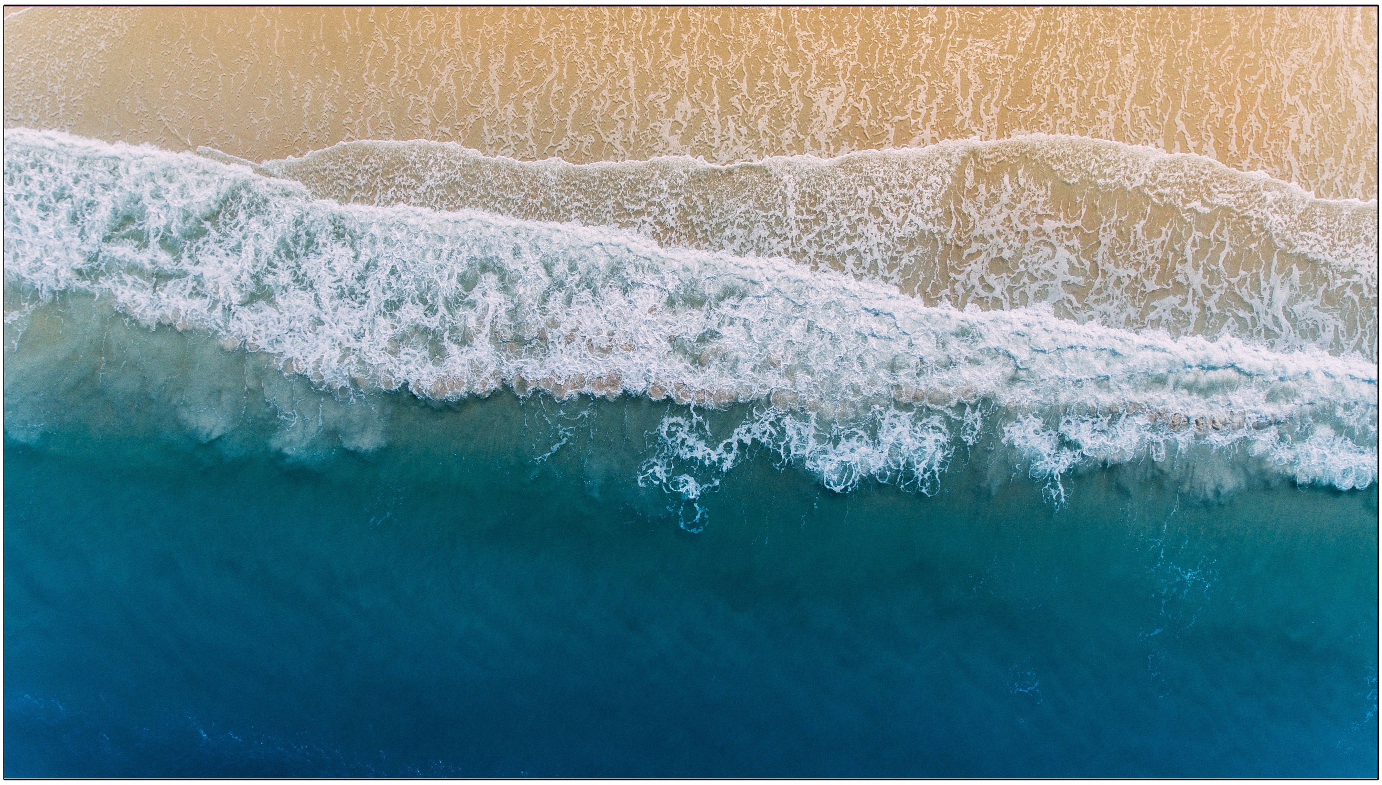

Time for a quick break!
STAND UP! SPIN AROUND!

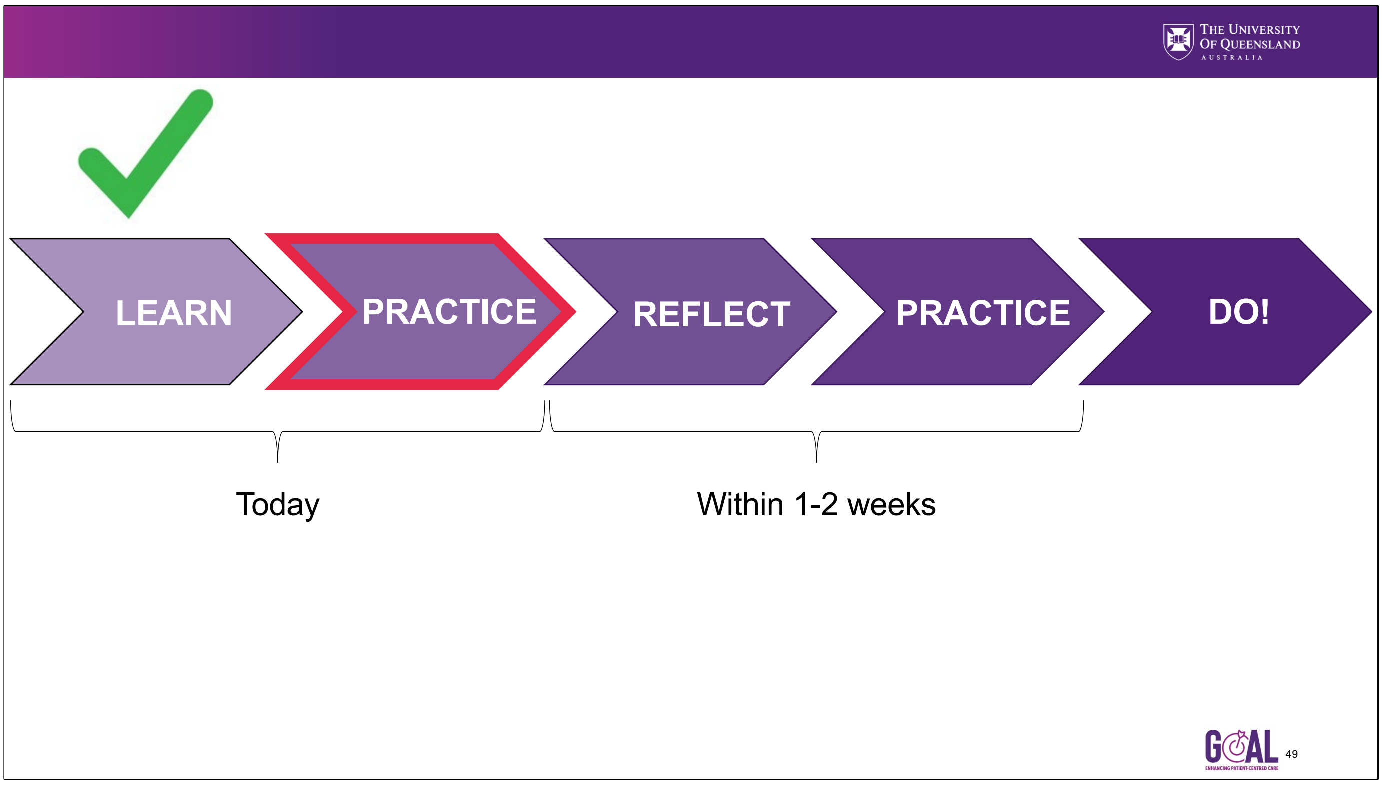

To recap, we have undertaken the theory component, so now it is time for some practice

Objectives

- Introduce **Goal Attainment Scaling** (GAS)
- Explain the **importance and benefits of goal setting**
- Discuss **SMART goals**
- Overview the **GAS template**
- Describe how to **set and scale a goal** for GAS
- Suggest **how to conduct meetings** with patients
- Allow an opportunity for **practical simulation**
- Offer tips on **troubleshooting common challenges**
- Describe the **review and scoring process**

Simulation scenarios

- Breakout groups of 2-3 people
- Rotate through the “facilitator” role
- Use the tools in the GAS Training Manual

Timing:

- Planning – 5 minutes
- ”Meeting” to set goals – 10 minutes
- Reflection and feedback – 5 minutes
  (Facilitator to be timekeeper)

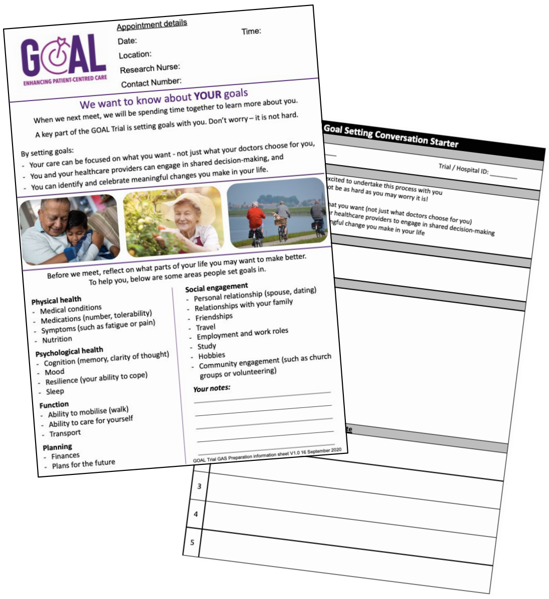

51

REVEAL
I will shortly place you in breakout groups of 2-3 people.

REVEAL
You will each have a chance to be the “facilitator” to have practice in walking a patient through the process of setting goals.

We know that simulation scenarios have their limitations, but try your best to contribute to the fidelity of the exercise.

REVEAL
Utilise the GAS Training Manual – specifically the GAS preparation information sheet and the conversation starter document.

REVEAL
Timing allowed for this exercise is:

- 5 minutes of planning
- 10 minutes of a meeting
- 5 minutes of reflection and feedback

Facilitator to keep time

ANY QUESTIONS?

Reflection and feedback

- Facilitator to share their self-reflections first
- Patient and observer roles to then share their feedback

Suggested structure:

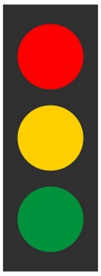

Stop doing

Start doing

Keep doing

52

At the end of the simulation, you will have the chance to self-reflect and offer feedback.

REVEAL
The person playing the facilitator role should offer their own self-reflections first. This is the followed by the ”patient” and the “observer”.

REVEAL
A way you can approach this is via the “Stop doing, Start doing, Keep doing” method. Think about:
- What worked well and should keep doing
- What didn’t work well and should not continue
- What was not done that could begin being done in the future

THE UNIVERSITY OF QUEENSLAND
AUSTRALIA

Simulation scenario – One

Group	Facilitator	Patient
A		
B		
C		
D		

TO POPULATE BASED ON ATTENDEES

GOAL
ENHANCING PATIENT-CENTRED CARE

53

SLIDE TO BE POPULATED BASED ON ATTENDEES

If there are uneven numbers, then trainer (i.e. Bonnie or Benignus) to pair up with a participant.

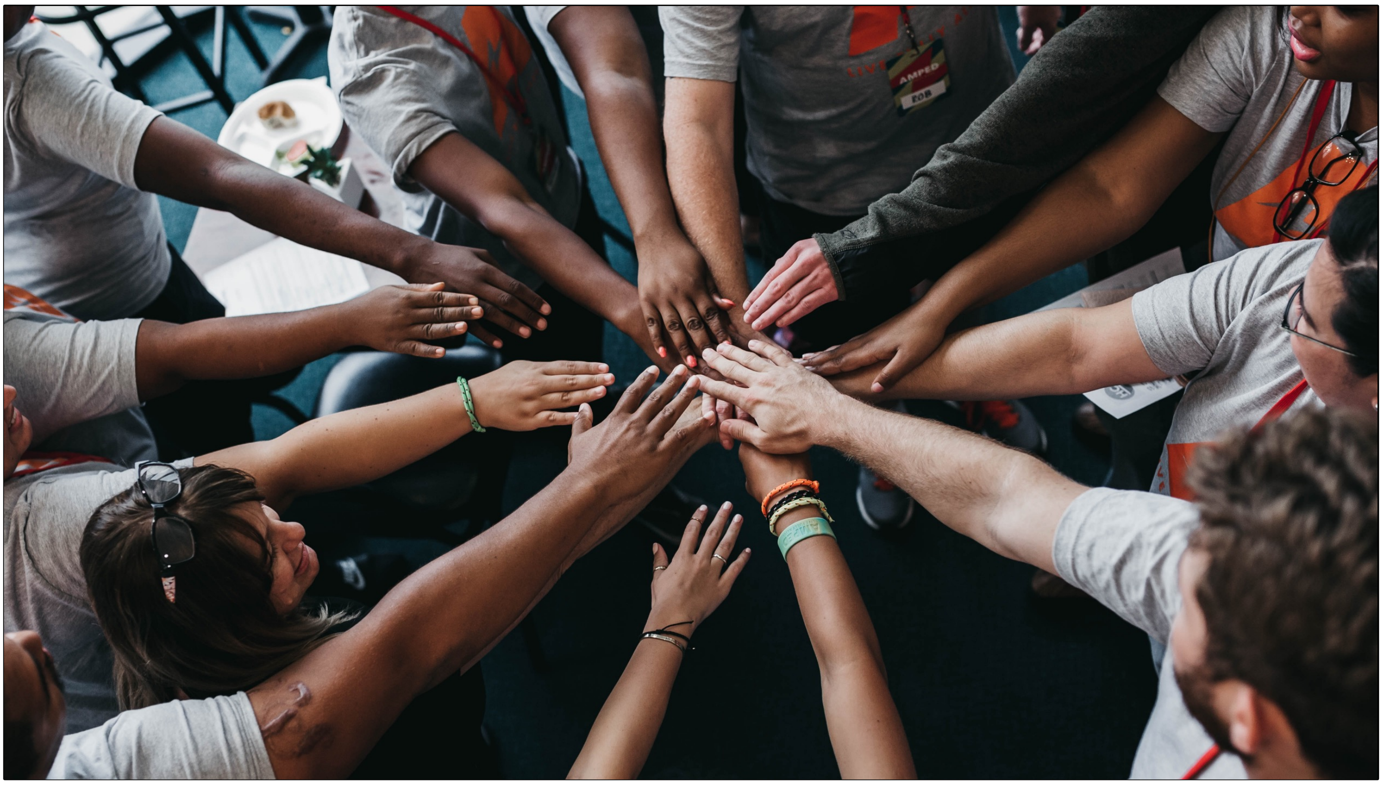

Time for huddle! Let’s share some reflections

(Ask the group for their thoughts on how they went and what questions the exercise prompted for them)

THE UNIVERSITY OF QUEENSLAND AUSTRALIA

Simulation scenario – Two

Group	Facilitator	Patient
A		
B		
C		
D		

TO POPULATE BASED ON ATTENDEES

GOAL ENHANCING PATIENT-CENTRED CARE 55

SLIDE TO BE POPULATED BASED ON ATTENDEES

If there are uneven numbers, then trainer (i.e. Bonnie or Benignus) to pair up with a participant.

!! For second scenario, keep the same pairs to avoid having to reassign the “breakout rooms” which can be time consuming

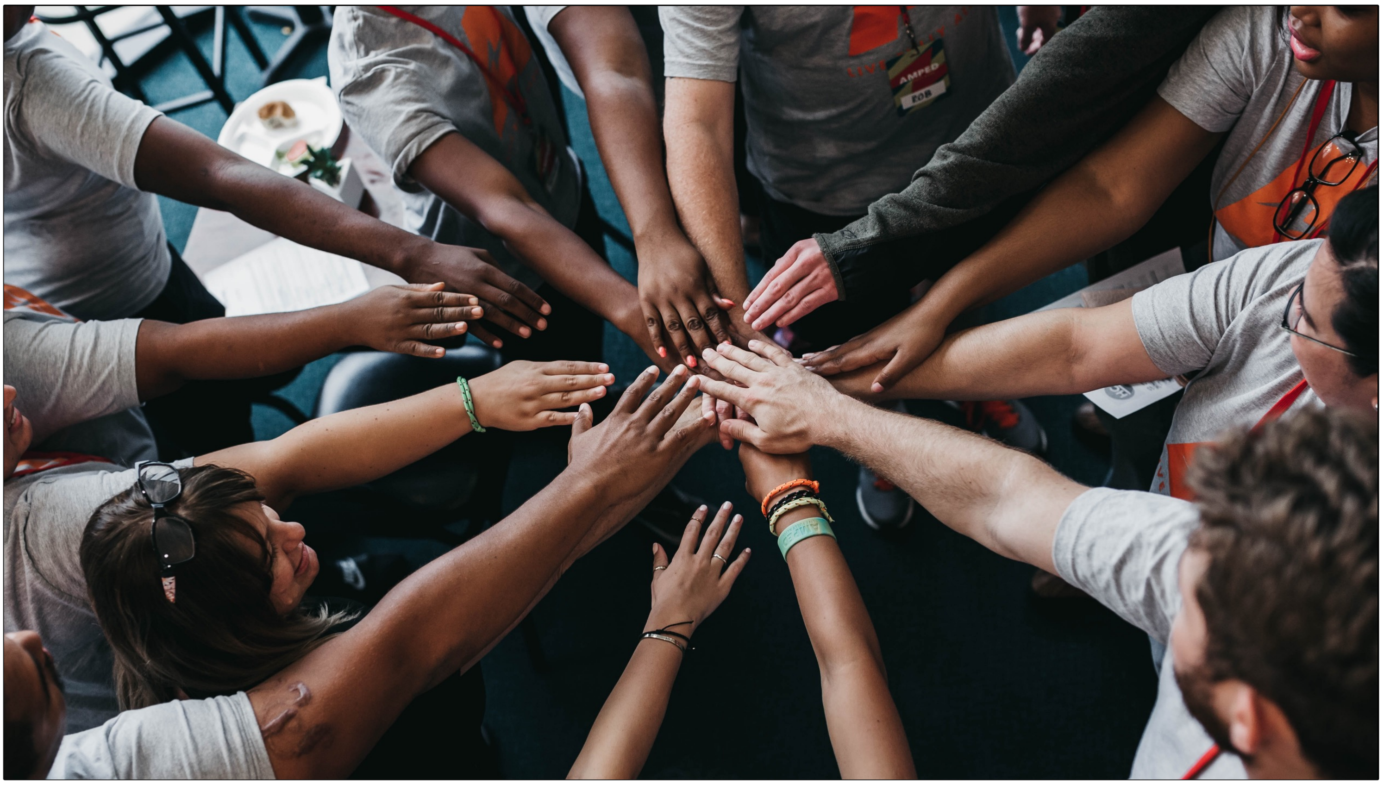

Time for huddle! Let’s share some reflections

(Ask the group for their thoughts on how they went and what questions the exercise prompted for them)

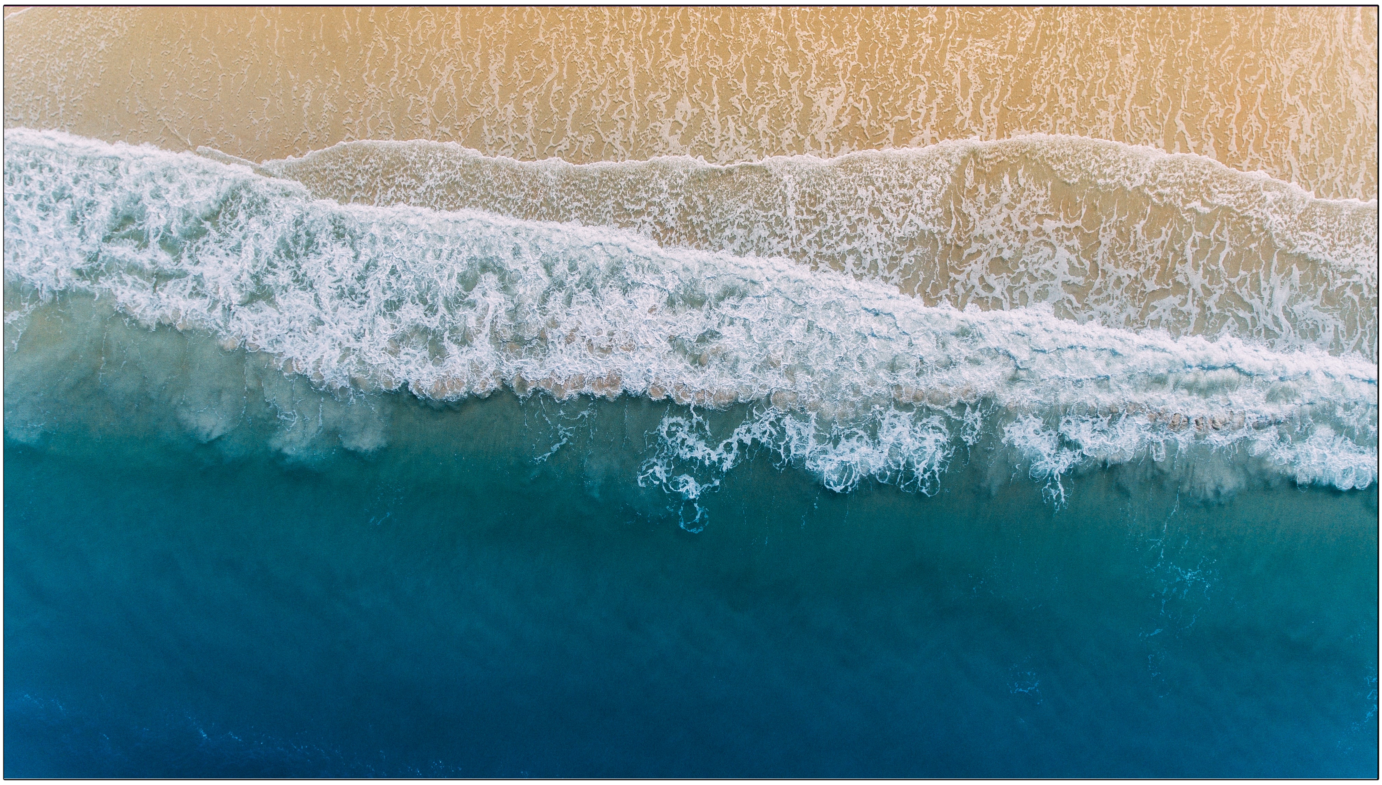

Time for a quick break!
STAND UP! SPIN AROUND!

Objectives

- Introduce **Goal Attainment Scaling** (GAS)
- Explain the **importance and benefits of goal setting**
- Discuss **SMART goals**
- Overview the **GAS template**
- Describe how to **set and scale a goal** for GAS
- Suggest **how to conduct meetings** with patients
- Allow an opportunity for **practical simulation**
- Offer tips on **troubleshooting common challenges**
- Describe the **review and scoring process**

The University of Queensland Australia

Troubleshooting common challenges

Writing goals

- Two variables
- Unrealistic expectations
- Too long-term
- Unable to articulate measures

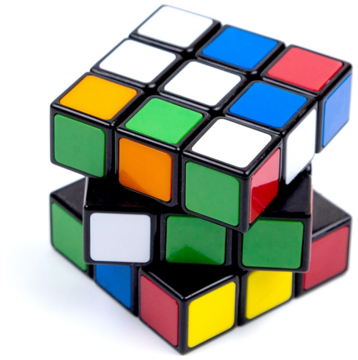

GOAL Enhancing patient centred care 59

There are two sets of challenges

1. Writing the goal
2. Engaging with the patient

REVEAL (Graduated)
With respects to writing goals, some common issues include:

- Two variables
- Unrealistic expectations
- Too long-term
- Unable to articulate measures

Detailed suggestions are included in the GAS Training Manual as to how to approach these challenges.

You also have the option of speaking with myself or Bonnie if you get stuck.

Any of these things you want to touch on now?

THE UNIVERSITY OF QUEENSLAND AUSTRALIA

Troubleshooting common challenges

Patient engagement

- Non-attendance at meetings
- Poor motivation
- Fear of failure
- Overwhelmed
- Unfamiliarity of facilitator with subject matter
- Poor literacy
- Culturally and linguistically diverse patients
- Sensory impairments (hearing or vision)
- Deterioration in health

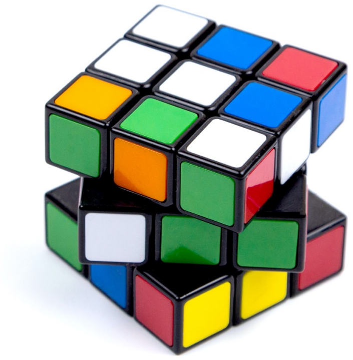

GOAL ENHANCING PATIENT CENTRED CARE 60

REVEAL (Graduated)

With respects to patient engagement, some common issues include:

- Non-attendance at meetings
- Poor motivation
- Fear of failure
- Overwhelmed
- Unfamiliarity of facilitator with subject matter
- Poor literacy
- Culturally and linguistically diverse patients
- Sensory impairments (hearing or vision)

Again, suggestions are included in the GAS Training Manual as to how to approach these challenges.

ANY QUESTIONS – OR ANY CHALLENGES YOU WANT TO SPEAK ABOUT NOW?

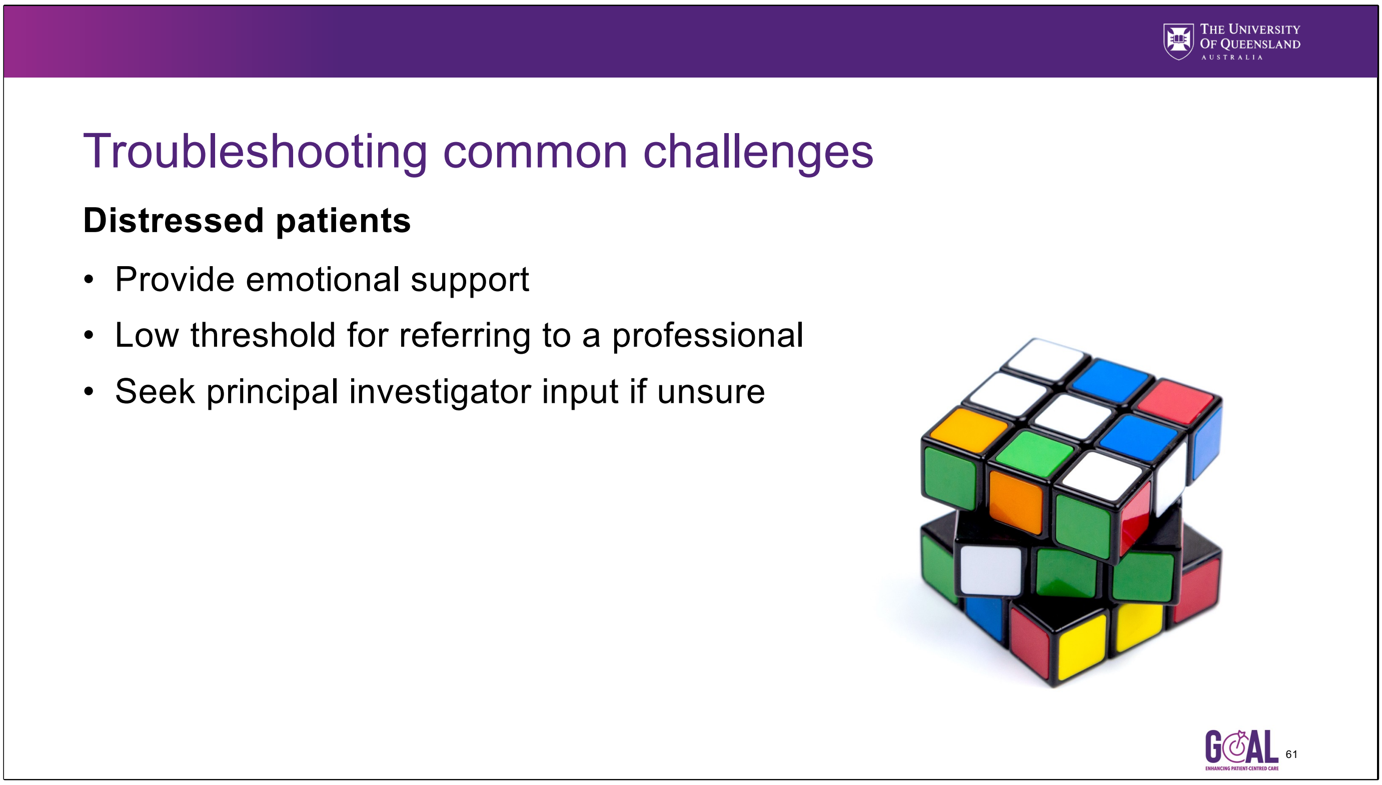

It is conceivable that participants may become upset during the GAS process.

Discussing matters such as a loss of function or social isolation may cause distress.

REVEAL
If this occurs, the facilitator should seek to provide emotional support initially.

REVEAL
Depending on the situation, and the degree of distress, there should be a low threshold for referring the patient to a qualified professional. This may be the site's local psychologist or mental health team, or referral back to the GP for access to community-based services.

REVEAL
If you are unsure as to the appropriate action they are to contact the site's principal investigator for their input.

The University of Queensland Australia

Objectives

- Introduce **Goal Attainment Scaling** (GAS)
- Explain the **importance and benefits of goal setting**
- Discuss **SMART goals**
- Overview the **GAS template**
- Describe how to **set and scale a goal** for GAS
- Suggest **how to conduct meetings** with patients
- Allow an opportunity for **practical simulation**
- Offer tips on **troubleshooting common challenges**
- Describe the **review and scoring process**

GOAL ENHANCING PATIENT-CENTRED CARE 62

Review and scoring

- Occurs at three months
- 30 minute meeting
- Meeting an expected outcome is good!
- Remain as neutral as possible
- Scoring to be based on ‘usual’ performance
- Score at the lower level if between two outcomes

- **Goals are not reset at the review meeting**

63

REVEAL
The review process is some three months away – so the value in discussing this step at length is limited.

I did want to flag some key points (**REVEAL**)
- It will be a thirty-minute meeting
- Achieving an “expected” outcome is good – they met their goals!
- Remain as neutral as possible – given the GAS is a study outcome, we do not want to risk encouragement or support being an uncontrolled variable
- Performance level should be considered based on what they have done consistently in the past fortnight (depending on the measure), as opposed to what was done on one occasion or only attained when the patient was “having a good day”. For example, if the goal is to walk 20 metres, consider asking whether they have been walking that distance most days in the last week. Further you would want to check that is not just the distance they are able to walk in a session with a physiotherapist at their side keeping them focussed and motivated
- Where the patient’s performance is between two predefined levels, they should be scored at the lower level
- Goals are not reset at the review meeting

ANY QUESTIONS?

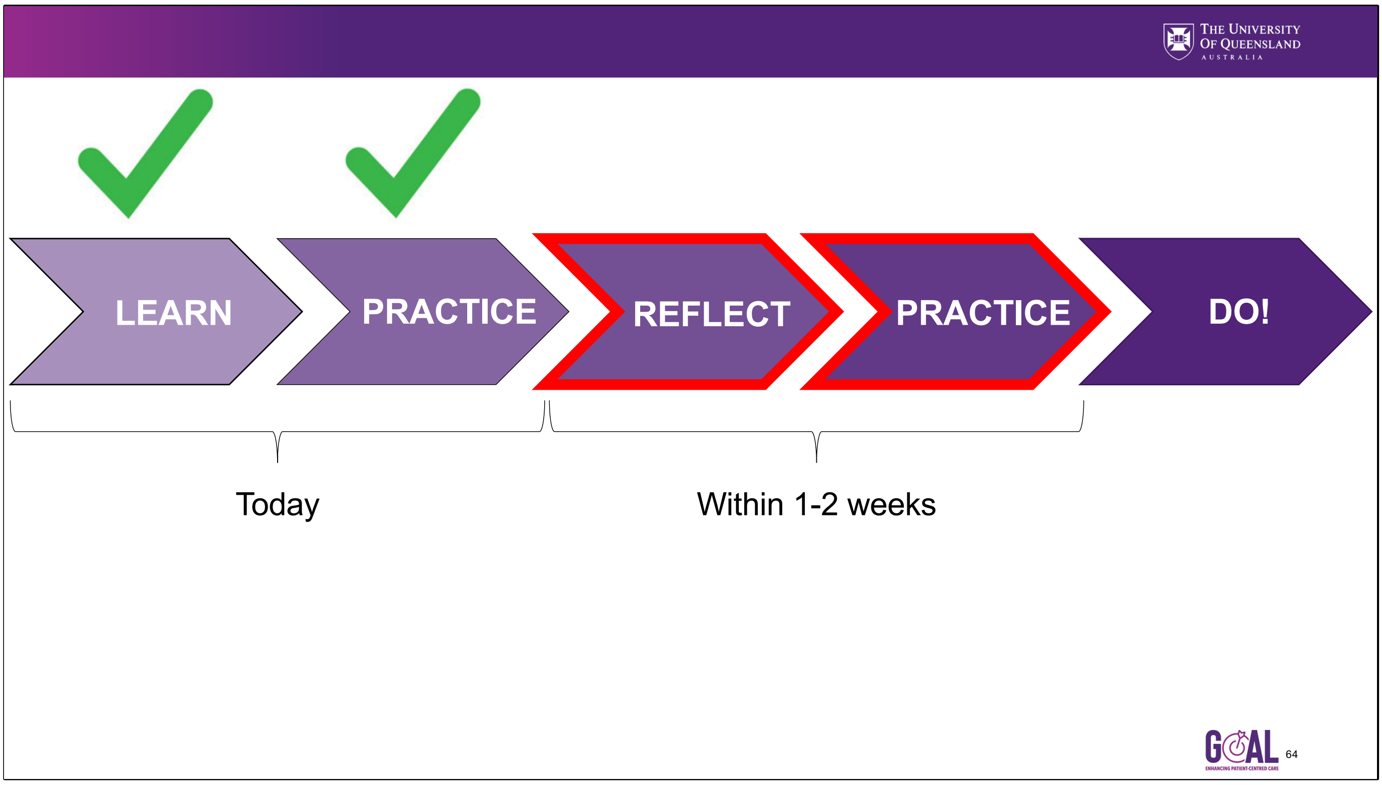

We have now concluded today’s training.

Let us take a moment to now overview where to from here.

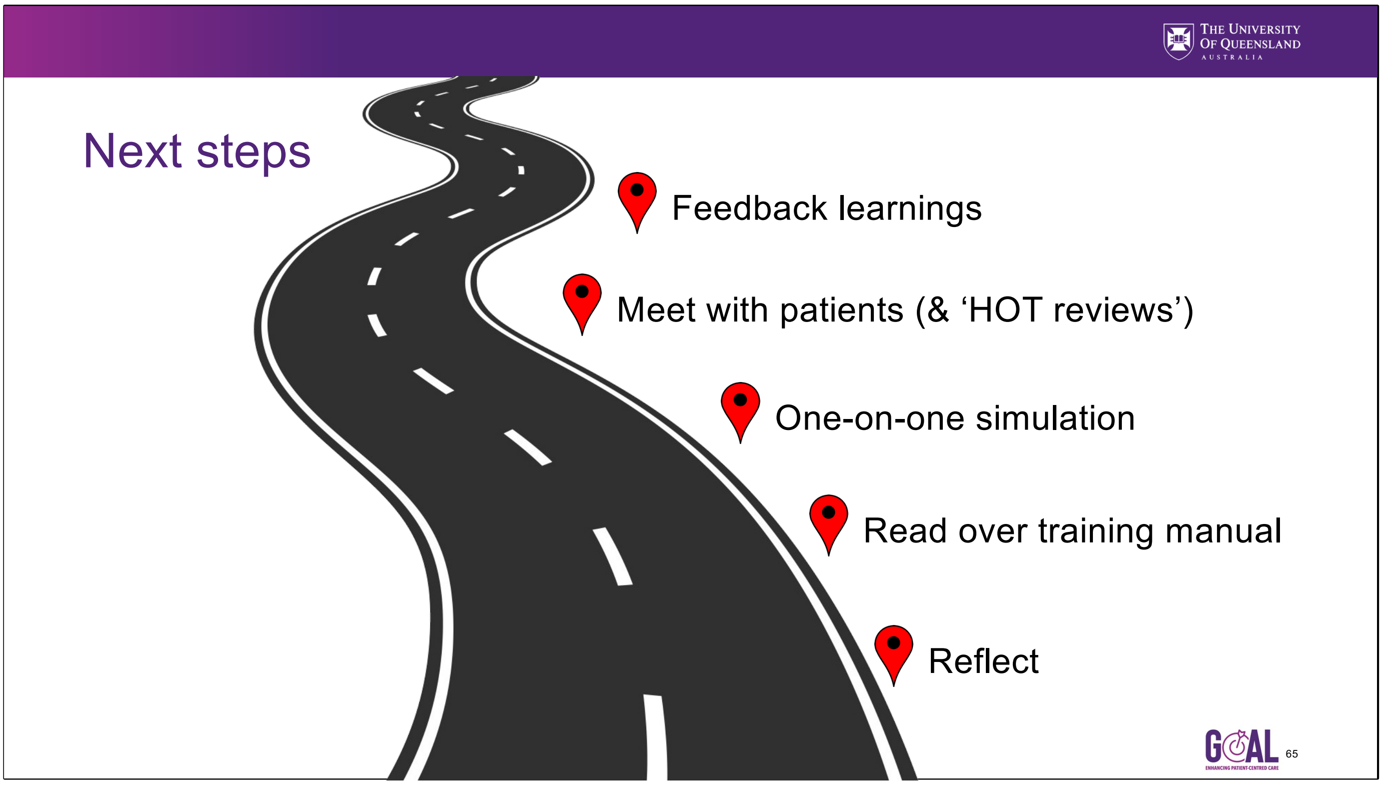

The next steps after today are **REVEAL**:

- Reflect on today’s training – capture any questions you have
- Read over the GAS Training Manual – and note any concerns you have
- I will be in touch to schedule a one-on-one simulation
  (Will take about ~30 minutes, with the opportunity to ask any questions you have gathered)
- Then it will be time to meet with your patients
  (The first five will get a HOT Review by either Bonnie or myself – will give you the plan for this at our one-on-one catchups)
- Throughout the process, please feel free to feedback any learnings

GOAL

ENHANCING PATIENT-CENTRED CARE
