# Supplementary material for: Training healthcare professionals to administer Goal Attainment Scaling as an outcome measure
Source: J Patient Rep Outcomes. 2024 Feb 26;8:22. doi: 10.1186/s41687-024-00704-0 (PMC10897066; doi:10.1186/s41687-024-00704-0)
Supplement: Supplementary file 3 — Supplementary File C: Worksheets for scenario-based learning [file 41687_2024_704_MOESM3_ESM.pdf]

Simulation scenarios for GAS classroom training

Instructions

This exercise will offer the opportunity to work through the practical process of setting a goal using the Goal Attainment Scaling approach.

Required material:

- Blank Goal Attainment Scaling template
- Goal domains – patient version
- Goal setting conversation starter

Work in groups of 2:

- Person 1: Take on the role of the **facilitator**, to help the "patient" set a goal
- Person 2: Play the role of the **patient**, using the provided briefing information as your reference

(There are two scenarios provided so each person can take a turn as the facilitator)

Timing:

- You will have 5 minutes initially, during which:
  - the facilitator should consider how they would like to structure their meeting, and
  - the patient should read through the briefing document and ready themselves to play their role
- There will be 10 minutes during which the facilitator will lead the patient in setting a goal
- To conclude, 5 minutes will be set aside for feedback and reflection:
  - use the "Keep doing", "Stop doing" and "Start doing" feedback approach so constructive feedback is provided (to be used by the participant acting as the facilitator when articulating self-reflection),
  - facilitator to share their self-reflection first, then the observer and patient to offer their thoughts

Note:

- You should set at least one goal by the end of this exercise, but in some situations setting more than one may be required if the conversation between facilitator and patient leads that way
- In setting the goal, you should also stipulate the importance and difficulty weighting

Briefing sheet for “Facilitator”

Scenario #1

You will shortly meet with a patient with the following demographics:

- 62 years of age
- Lives alone
- Able to mobilise without any walking aids
- Most significant health conditions are asthma, lower back pain, kidney disease and diabetes

Please work with them to set a goal, and weight it, using the Goal Attainment Scaling approach and template.

Briefing sheet for “Patient”

TO ONLY BE ISSUED TO THE INDIVIDUAL PLAYING THE “PATIENT” ROLE

Scenario #1

Demographics:

- 62 years of age
- Lives alone
- Able to mobilise without any walking aids
- Most significant health conditions are asthma, lower back pain, kidney disease and diabetes

Patient demeanour and mindset:

- Poor health literacy
- Hesitant to talk too freely as worried about embarrassing themselves given their poor knowledge
- Never has set a goal before

Broad list of areas they thought they might set a goal in:

- Walk more
- Use less tablets – don’t like using the painkillers
- Want to look into doing some courses or study to keep your mind engaged

Ultimate goal you decide you want to set:

- Use less endone!

Relevant contextual information:

- Currently use endone for lower back pain
- Been using endone for three years, via a GP prescription
- Over past three months been using more
- In the last three months your friend you used to walk with daily has moved away so you are doing less exercise
- Using typically four 5mg tablets now, but used to use only two 5mg tablets every two days
- You think it will be really quite difficult to achieve this goal, but think it is important as you don’t like painkillers and have read they can be bad for you

Take the liberty to make appropriate assumptions for any other details required.

Briefing sheet for “Facilitator”

Scenario #2

You will shortly meet with a patient with the following demographics:

- 75 years of age
- Recently lost their spouse to cancer
- Retired nurse
- Has a devoted daughter and grandkids
- Most significant health conditions are high blood pressure and depression

Please work with them to set a goal, and weight it, using the Goal Attainment Scaling approach and template.

Briefing sheet for "Patient"

TO ONLY BE ISSUED TO THE INDIVIDUAL PLAYING THE "PATIENT" ROLE

Scenario #2

Demographics:

- 75 years of age
- Recently lost their spouse to cancer (three months ago)
- Retired nurse
- Has a devoted daughter and grandkids who are keen for you to get out more
- Most significant health conditions are high blood pressure and depression

Patient demeanour and mindset:

- Sad after loss of spouse
- Somewhat lacking in motivation in general – in a bit of a 'funk' at present
- Easily engaged in conversation

Broad list of areas they thought they might set a goal in:

- Reconnect with social circle
- Sleep better
- Learn a language – always dreamt of traveling to France
- Talk with a financial adviser about planning for the future now your spouse is gone

Ultimate goal you decide you want to set:

- Get out of the house more – need to try and keep yourself more socially engaged

Relevant contextual information:

- Used to be a self-described "social butterfly" a few years ago – with interests including art classes, playing bridge, and going to yoga.
- Usually you were out every day to an activity. Now only leave once a week to go to Coles.
- The last two years have been increasingly dedicated to caring for your spouse as they battled cancer
- You can drive, and have your own car
- You don't think it will be too difficult for you, and think it is worth prioritising this goal

Take the liberty to make appropriate assumptions for any other details required.
