# Supplementary material for: Training healthcare professionals to administer Goal Attainment Scaling as an outcome measure
Source: J Patient Rep Outcomes. 2024 Feb 26;8:22. doi: 10.1186/s41687-024-00704-0 (PMC10897066; doi:10.1186/s41687-024-00704-0)
Supplement: Supplementary file 4 — Supplementary File D: A practical guide to administering Goal Attainment Scaling for the GOAL Trial [file 41687_2024_704_MOESM4_ESM.pdf]

A practical guide to administering Goal Attainment Scaling (GAS) for the GOAL Trial

Version control

Version: 01.00

Date: 14 December 2020

Key contacts

For further information and support, please contact:

Dr Benignus Logan
benignus.logan@uq.edu.au

Ms Bonnie Pimm
b.pimm@uq.edu.au

Contents

Purpose of this guide **4**
An introduction to Goal Attainment Scaling (GAS) **4**
The importance and benefits of goal setting **5**
Stakeholders **5**
Patient 5
Caregiver 5
Facilitator 5
Researchers 5
Clinicians 6
Orientation to the GAS template **6**
Goal domains **8**
Understanding SMART goals **8**
Specific 9
Measurable 9
Achievable 9
Relevant 9
Timebound 9
Meeting with a patient to set goals **9**
Before the meeting 9
Location and timing 9
Preparatory work 10
During the meeting 10
Required material 10
Opening 10
Orientation to template and session 11
Brainstorm possible goals 11
Identify goals 12
Scaling goal outcomes and making them SMART 12
Set a review date 14
Nominate a weighting 14
Action plans to achieve set goals 14
Closing 14
A note on meetings held over Zoom 15
After the meeting 15
Review 15
Write-up and distribution 15
Meeting with a patient to review their goals **15**
Before the meeting 15
Location and timing 15
Preparation 16
During the meeting 16
Required material 16
Opening 16
Scoring goal attainment 16

Resetting goals 17
Review of action plans 17
Closing the meeting 17
After the meeting 17
Calculating the overall GAS score 17
Worked example of setting a goal 18
Approaches to troubleshooting common challenges 18
The challenges of writing a goal 18
Two variables 18
Unrealistic 19
Too long-term 20
Unable to articulate measures 20
Goals related to emotional distress 20
The challenges of patient engagement 20
Non-attendance at meetings 20
Poor motivation 20
Fear of failure 21
Overwhelmed 21
Unfamiliarity of facilitator with subject matter 21
Poor literacy 21
Culturally and linguistically diverse patients 21
Sensory impairments (hearing or vision) 21
Deterioration in health 21
Supporting participants in distress 22
Actions to aid effective implementation of GAS 22
Training of facilitators 22
Simulation exercises 22
Hot review 22
References 24
Appendices 26
Appendix A: Goal Attainment Scaling – blank template 27
Appendix B: Goal Attainment Scaling – populated example 29
Appendix C: GAS preparation information sheet 31
Appendix D: Goal setting conversation starter 33
Appendix E: Goal setting conversation starter – populated example 35
Appendix F: Zoom-based conversation starter & GAS template 37

Purpose of this guide

This guide has been developed to enable research nurses, and their delegates, to confidently and consistently administer the Goal Attainment Scaling (GAS) instrument irrespective of their knowledge base and skillset.

Significant time has been invested into supporting the effective implementation of the GAS process as it is the measure for the GOAL Trial's primary outcome. Hence it is essential to be correctly done from the outset.

An introduction to Goal Attainment Scaling (GAS)

Goal Attainment Scaling (GAS) is a tool which documents a patient's goals, and then scores the extent to which they are attained. The goals are unique to the patient and their situation. The scoring of their attainment is standardised on a scale from "much less than expected" through to "much better than expected".

GAS was first developed in the 1960s[1]. Since that time it has been deployed in research and clinical practice, most frequently in geriatric and general rehabilitation medicine[2-4], but also in paediatrics[5,6], community health[7,8], pain medicine[9], mental health[10] and haematology[11]. It can be undertaken in either inpatient[12] or outpatient settings[13].

The basic steps to GAS are outlined in the below table.

Step	Detail
1	Identify a goal
2	Define the current state
3	Identify potentially better and worse outcomes
4	Weight the goals
5	At follow-up, compare their current status with their baseline status

***Table 1**: Steps of Goal Attainment Scaling – adapted from: Rockwood. Appendix 2 - Attainment of treatment goals by people with Alzheimer's disease receiving galantamine: a randomized controlled trial[14]*

Patients are able to set one to five goals which meet the "SMART" criteria. Specifically, the goals will be specific, measurable, achievable, relevant and timebound.

For each goal, possible outcomes are scored as a number value between -2 and +2. If the patient attains the expected level, this is scored at 0. If they attain a better outcome this is scored at +1 (somewhat better than expected) or +2 (much better than expected). If they attain a worse outcome this is scored at -1 (somewhat less than expected) or - 2 (much less than).

The patient's baseline performance will typically be scored at -1, unless there is no worse outcome conceivable from their current status in which case it will be scored at -2.

Each goal will be weighted based on the difficulty and the importance it holds to the patient on a scale of 1 to 3 (with 1 being the bottom end of the scale and 3 the top).

The importance and benefits of goal setting

Patient-identified goals are needed to underpin patient-centred care. The benefits of involving patients in their care, through goal setting, include:

- Focusing on individually-desired, rather than system-set, results[15].
- Measuring, and thus allowing praise of, meaningful changes achieved by a patient.
- Providing a tool for collaboration and communication between patients and their healthcare professionals in the interests of shared decision-making[15].
- Unifying attention on individualised outcomes rather than potentially disparate and conflicting disease-specific outcomes[16].

The advantage of GAS as the tool to set goals in research settings is its ability to be deployed as an outcome measure in heterogenous patient groups[17,18].

Stakeholders

Patient

The most important stakeholder in this process is the patient. It is key that all efforts are made to engage them meaningfully to best elicit their thoughts and understand what is valued by them. To be able to engage in the GAS process, the patient should have no significant cognitive impairments. They need to think about what they would like to achieve, and how they could achieve it, in the setting of their physical, functional, social and psychological status. Patients are supported through the GAS process by a trained facilitator.

Caregiver

Depending on the clinical or research setting, the involvement of a patient's caregiver or support person may be appropriate. A patient's loved one can sometimes be well positioned to help prompt reflection and provide important collateral history to inform what is realistic and achievable for a patient. A support person can be present for the GAS meeting.

Facilitator

Any number of healthcare professionals or researchers can assume the facilitator role. This includes doctors, nurses, allied health professionals and research assistants. To undertake the facilitator role, the individual requires familiarisation with the GAS process, through the use of a resource such as this one and have some basic understanding of the patient group that is being engaged. For the GOAL Trial, there will be formal training provided with a theoretical component followed by practical simulations.

Researchers

In a research setting, GAS can be used as an outcome measure[18]. The design of a particular research project will determine the role researchers play in the GAS process. For the GOAL Trial, the researchers will provide training and support to the research nurses at each site, to best enable them to be effective facilitators. They will use the data collected as a key outcome measure.

Clinicians

Whilst General Practitioners and other treating specialists are important stakeholders, given the trial's design they will not be involved in the GAS process. Given GAS will be an outcome measure, the involvement of clinicians could be an uncontrolled variable that contaminates results.

Orientation to the GAS template

The GAS template is included as Appendix A, with a smaller-scaled version below. The key components are discussed in more detail below.

Where meetings occur in person, print out this template and work through the goal setting process as a paper-based tool. Where meetings need to occur virtually, display the document via a "shared screen" function.

Goal Attainment Scaling for <insert name> Trial ID: __________

Date set: __________
Review date: __________ Timepoint: ☐ Baseline ☐ 3 months ☐ 6 months ☐ 12 months

**Domain**					
**Goal**					
**Much less than expected (-2)**					
**Somewhat less than expected (-1)**					
**Expected level (0)**					
**Somewhat better than expected (+1)**					
**Much better than expected (+2)**					
**Importance (1-3)**					
**Difficulty (1-3)**					

# indicates baseline performance *(Scale for importance and difficulty: 1 = fairly; 2 = very; 3 = extremely.)*

Image 1*: Goal Attainment Scaling – blank template*

The top portion of the template is shown below. The date the GAS process was initiated with goals being set should be noted, as well as the planned review date. This ensures the goals are timebound. The review date should reflect when the first review is planned. This template has been designed with an intention of reviews at three, six and twelve months, with checkboxes able to be marked to reflect this.

Goal Attainment Scaling for <insert name> Trial ID: __________

Date set: __________
Review date: __________ Timepoint: ☐ Baseline ☐ 3 months ☐ 6 months ☐ 12 months

Image 2*: Top portion of the Goal Attainment Scaling template.*

At the top of the table, there is space provided for the goal to be written (as shown by the below image). The domain (further explained below) is to be populated with the most appropriate item from the available menu.

**Domain**		
**Goal**		

Image 3*: Top of the Goal Attainment Scaling table.*

The bulk of the table provides space, as shown below, for the baseline and attainment measures to be articulated in detail. The measures correspond to a numerical score as follows:

- Much less than expected: -2
- Somewhat less than expected: -1
- Expected level: 0
- Somewhat better than expected: +1
- Much better than expected: +2

**Much less than expected (-2)**		
**Somewhat less than expected (-1)**		
**Expected level (0)**		
**Somewhat better than expected (+1)**		
**Much better than expected (+2)**		

Image 4*: Middle of the Goal Attainment Scaling table.*

The bottom part of the table prompts for a weighting to be recorded with respects to each goal's importance and perceived difficulty according to the patient. The weighting ranges from 1 for a "fairly" important or difficult goal, through to 3 for an "extremely" important or difficult one, and should be determined by the patient.

**Importance (1-3)**		
**Difficulty (1-3)**		

Image 5*: Bottom of the Goal Attainment Scaling table.*

Goal domains

A list of domains is available for patients to help identify areas in which they may wish to consider setting goals. This also assists with data collection when GAS is used in a research setting.

The goal domains presented here not only draws on work published about GAS by researchers lead by Professor Rockwood[17,19], but also takes into account priorities identified via nominal group technique by patients with chronic kidney disease[20].

For each goal that is set, the facilitator working with the patient is required to map it to the single most appropriate domain from the list below. There is no requirement for a patient to set goals in different domains. If a patient desires, all of their goals may relate to the one domain. If there is no domain which matches a patient's goal, then that does not preclude them setting that goal.

Class	Goal Domains
Physical wellbeing	Medical conditions (including risk factor modification)<br>Medications (administration, tolerability, number taken)<br>Symptoms (fatigue, pain etc)<br>Nutrition
Psychological wellbeing	Cognition (memory, clarity of thought)<br>Mood<br>Resilience (ability to cope)<br>Sleep
Functioning	Ability to mobilise (walk)<br>Ability to care for yourself<br>Transport
Social engagement	Personal relationship (i.e. spouse, dating)<br>Relationships with your family<br>Friendships<br>Travel<br>Employment and work roles<br>Study<br>Hobbies<br>Community engagement (church, volunteering etc)
Planning	Finances<br>Plans for the future

***Table 2**: Domains for goal setting in Goal Attainment Scaling – with some adaption from: Urquhart-Secord R, Craig JC, Hemmelgarn B, Tam-Tham H, Manns B, Howell M, et al. Patient and Caregiver Priorities for Outcomes in Hemodialysis: An International Nominal Group Technique Study[20]; Stolee P, Rockwood K, Fox RA, Streiner DL. The Use of Goal Attainment Scaling in a Geriatric Care Setting[17]; and Morrow-Howell N, Yip AM, Gorman MC, Stadnyk K, Mills WGM, Macpherson M, et al. A Standardized Menu for Goal Attainment Scaling in the Care of Frail Elders[19].*

Understanding SMART goals

A goal should meet the SMART principle of being Specific, Measurable, Achievable, Relevant and Timebound[21]. The GAS template is structured in such a way as to help enable this.

Specific

To be specific, it needs to be clear what problem or desired change is being targeted. If a patient wishes to “walk better”, the facilitator should help them articulate whether that means to walk a longer distance, to walk without an aid or to walk without any pain. If all three are important to them then three separate goals should be listed.

Measurable

As much as possible, an objective measure needs to be stated for a goal. Objective measures are easy to determine where the goal can relate to a frequency (“cook dinner three nights a week”) or distance (“cycle ten kilometres”). Sometimes a degree of subjectivity has to be accommodated. For example, whilst “have less pain” may be seen as subjective, it can be made more objective by reframing it as “reduce my pain levels from 8/10 with activity, to 4/10 with activity” or “use only one 5mg oxycodone a day”.

Achievable

Patients, with prompting from their facilitator, will have to consider how achievable their desired goal is. Important considerations include reflecting on when they last were able to achieve that outcome and how far from being able to do it now. For example, if their goal is to walk 50 metres it would be important to probe when they last walked that far and to understand how far they comfortably walk now.

Relevant

Goals should be of relevance to the patient, and given they are setting them they intuitively should be. It is not necessary for a carer or clinician to endorse its relevance. The facilitator may wish to prompt the patient to reflect on the benefit or purpose that attaining the goal would bring.

Timebound

The date for achieving the goal needs to be stated. The patient should consider whether their goal is something which is likely to occur in the time period available. The date for achieving the goal is to be **three months**, in keeping with the trial’s primary outcome measure. The subsequent interval assessment at six and twelve months is a secondary outcome. **Please note you do not revise any goals at three months**.

Meeting with a patient to set goals

Before the meeting

Location and timing

Patients should be met face-to-face where possible. This is most conducive to building rapport. If circumstances prevent it, a video call would be the next best alternative via the Zoom platform or similar.

Set aside 45 minutes for the initial meeting about GAS. Ideally this will coincide with the completion of the other baseline assessments (total meeting time of 75 minutes).

Preparatory work

Self-reflection is important for setting goals that are relevant to the patient. To prompt this desired self-reflection, a preparation information sheet on goal setting will be issued prior to the meeting. Appendix C demonstrates an example.

For the GOAL Trial, this preparation information sheet will be issued after a patient has been consented and confirmed as being eligible for the trial.

When a patient is provided with the information sheet on goal setting, the healthcare professional in attendance may wish to consider reinforcing key messages including:

- They will be well supported in the goal setting process.
- It is intended to be about them and what they value.
- There is no "wrong" or "right" idea.

The healthcare professional may also wish to provide some practical information at the time of issuing the information sheet, including:

- Let the patient know there is room to write notes in the bottom right corner for any ideas they have.
- Suggest the patient circle or underline any of the goal domains which resonate with them.
- Notify the patient that they can bring a support person (a spouse, a carer or other loved one) to the meeting.

This information sheet also sets out the importance of goal-setting so patients can begin to understand "the why" behind this process, which may help somewhat in overcoming reticence of patients to engage in this process.

The intent for the information sheet, along with how it is introduced, is to leave the patient in a positive mindset as they approach the goal-setting meeting, and to give them adequate time to think about what they may like to achieve.

During the meeting

Outlined below is a suggested approach a facilitator can take in meeting with a patient. A conversation guide is included (see Appendix E) as a tool which can be utilised to help structure the meeting.

Required material

- Pens and blank paper for notetaking
- Blank GAS template (see Appendix A)
- GAS preparation information sheet (see Appendix C)
- Goal setting conversation starter (see Appendix D)

Opening

In opening the meeting, the facilitator may wish to:

- Thank the patient for attending
  "*Thanks for coming along, I really appreciate your time*"

- Share a sense of excitement for the session
  "*I'm really excited to have the chance to spend this time with you to set some goals*"

- Offer reassurance that they will be well supported
  "*The process we will go through has a good structure around it to support you*"

- Reiterate the importance of goal setting
  "*We think that goal setting is important as it:*
  - helps care to be focused on what you want (not just what doctors choose for you)
  - provides a way for you and your healthcare providers to engage in shared decision-making
  - identifies and celebrates meaningful change you make in your life"

- Enquire if they have any questions before progressing
  "*Is there anything you wish to ask before we make a start?*"

Orientation to template and session

The patient should be oriented to the template that forms the basis of the GAS, and in doing so learn broadly about the required outcomes for the session.

The key messages to relay to the patient are:

- *"We will work together to set <u>one to five goals</u> that are important to you"*
- *"The initial <u>review timeframe is three months</u>"*
- *"We will use this form to document the goals"*
- *"I'll point out a few things on the form to explain it to you* (point accordingly):
  - *This sets out today's date and when we will review the goals next*
  - *The "domain" is the category the goal relates to – I'll fill this out, so don't worry about that*
  - *This is where we can write the goal*
  - *This is where we write down the various outcomes that might happen*
  - *This is where we write down how important and difficult the goal is"*

Brainstorm possible goals

One of the most challenging steps in the GAS process is identifying what broad goals a patient potentially has. At this point the facilitator should work with the patient to brainstorm all possible goals. There should be no judgement or moderation attempts made at this initial stage. Write down everything they say no matter how unworkable it may seem. The conversation guide (Appendix D) provides space for the options to be listed, which can be referred back to later. The list of goal domains (Appendix C) can be used to prompt conversation, and reflects the list which was issued in the patient information sheet prior to the meeting.

Prompting questions are outlined below as an example, but each facilitator will have their own tone and style:

- *"Did you have any goals you had thought about before coming in today?"*
- *"From this list of goal setting areas, does anything catch your eye?"* (see Appendix C)
- *"What things currently frustrate you in your life?"*
- *"If you could change one thing in your life what would that be?"*

- *"If your friends and family were here, what would they say you should focus on?"*
- *"If you think back five years, is there anything you no longer do which you wish you still did?"*
- *"If you had a 'magic wand', what would you change about your life?"*

If stuck, it may be best to refer to the list of goal domains and work through the options line-by-line to tease out anything the facilitator can work with.

Identify goals

From the list of potential goals identified through the above exercise, the facilitator now works with the patient to identify one to five of them to formalise. The conversation guide used for the above (Appendix D) has room to list the selected goals. At this point the goals can be very plainly worded, with further refinement to occur in the next stage of the process.

Scaling goal outcomes and making them SMART

From the list of identified goals, the facilitator needs to work with the patient on each goal one-by-one to scale it and ensure it meets the SMART philosophy. A suggested approach to achieve this is outlined in detail in the following, but is summarised here:

- Describe a measure
- Write the goal and domain
- Define baseline performance
- Define worse outcomes
- Define the expected outcome
- Define better outcomes
- Set a review date
- Nominate a weighting

Describe a measure

For GAS, **a single measure is needed for each goal**. For example if a patient wishes to "walk better", the facilitator will need to agree with the patient how that is best measured. It may be walking a longer distance (metres as a measure) or mobilise more independently (level of assistance as a measure). There cannot be multi-faceted measures in the one goal (i.e. 'metres' and 'level of assistance'). Should the patient have two measures that are identified as important to them, then two separate goals should be set.

For continuous measures (such as distances), measures should be provided in ranges.

Also key to describing a measure, is ensuring it can be **scaled to five different intervals**. For categorical measures, such as distance, this is relatively straightforward. Drawing on the above goal of "walking a longer distance", the five intervals could be scaled as: 80 metres; 60 metres; 40 metres; 20 metres; and 5 metres or less. For discrete measures this is more challenging but can often be achieved with some thought. Drawing on the above example of "mobilise more independently", the five intervals could be scaled as: able to mobilise without any mobility aid; mobilise with a single-point stick; mobilise with a four-wheeled walker; mobilise with a wheelchair via a stand-pivot transfer; and, patient bed bound, requiring a hoist.

Write the goal and domain

Once an appropriate measure is identified, the facilitator should now write the goal in the template in the "Goal" row. The goal should be written in plain language. In this part of the form it is not necessary to write a SMART goal, as the elements of that are set out elsewhere (specifically, in the scaling outcomes and the setting of a review date).

At this time, the facilitator should also populate the "Domain" with the single most relevant option available.

Define baseline performance

Once the goal is written, the facilitator will want to have the patient articulate their current performance. An accurate understanding of their baseline performance is needed so expected outcomes are appropriately set. It is important to appreciate what occurs most often, not what they do sometimes "on a good day".

It is also key to understand the **recency of their performance**, and its **trajectory**. For example, is their ability to walk 20 metres something that has been stable for some months, or is it a new high or low point? It is also worth prompting the patient to share what they were able to do three months ago.

The baseline performance will be written in the "somewhat less than expected (-1)" row. One exception to this is where there is no conceivable worse outcome than their current status. For example, if their broad goal is "to exercise more" and currently they do no exercise, then that would be written in the "much less than expected (-2)" row.

Another exception is where a patient has experienced a progressive decline in how they are performing in a particular aspect of their life. They may want focus on maintaining their current abilities and halt a deterioration. For example, a patient with kidney failure may have as their goal "to maintain a healthy body weight" in the setting of having lost one kilogram of weight for each of the past six months. For them it may be appropriate to set their baseline measure at the "expected level (0)" row.

Baseline performance is marked on the template by placing a hashtag (#) in the relevant box. Where continuous measures are used, and thus you need a range spelling out the actual current baseline as a discreet measure may be appropriate and reflected in the examples in this document.

Define worse outcomes

Once the baseline performance is defined, poorer performance outcomes can be articulated. This will be populated into the "much less than expected" row, unless the baseline performance is at that level.

Define the expected outcome

The most challenging part of the scaling process is working with the patient to describe expected performance. It is further complicated in situations where the goal relates to an area in which the facilitator may not be familiar. For example, setting goals to "walk further" or "walk with mobility aids" when the facilitator has no rehabilitation or physiotherapy exposure.

The complexities of this stage of the GAS can be addressed by:

- Understanding their current and prior performance levels, and the trajectory of this.
- Gently challenging the patient on what they see as being a stretch or comfortable for them.

- Seeking collateral history from any support person present at the goal setting session.

There is a fine balance in not being too lenient or too firm, particularly in research settings where the GAS is being used as an outcome measure.

For the GOAL Trial, no input is to be sought or accepted from the patient's treating medical and allied health team, as it risks contaminating the trial results.

Define better outcomes

Once the expected outcome is populated, attention can turn to defining what outcome would be "somewhat better" and "much better" than expected. Appropriate intervals between the measures should be chosen to ensure the spread between "much less than expected (-2)" and "much better than expected (+2)" is relatively spaced.

Set a review date

Whilst the exact date of review may vary slightly in the reality of arranging review appointments, the review date should be written as three months from the date goals are written down.

Nominate a weighting

The weighting for each goal should now be assigned. A score is required for difficulty and importance, as the patient perceives it. Both the difficulty and importance use the same scale:

- 1: Fairly important/difficult
- 2: Very important/difficult
- 3: Extremely important/difficult

All the goals can be weighted the same if the patient wishes. There is no requirement for the goals to be prioritised (i.e. "ranked") relative to each other.

Action plans to achieve set goals

Once a goal is formulated, setting out to achieve it is the next phase. In order to make an action plan, a patient will want to consider:

- what smaller steps they can aim for as they work towards the ultimate achievement of a goal,
- how to build the potential changes needed into their daily routines, and
- the informal and formal resources they can utilise (be it family supports, free online learning, input from their General Practitioner, or accessing the services of a physiotherapist etc).

To ensure there is no contamination of measuring the intervention's effect, the research nurse (or their delegate as the GAS facilitator) will **not** spend time developing an action plan. They can provide verbal guidance, but **only** by using the three talking points noted above.

Closing

In closing the meeting, the facilitator may wish to:

- Thank the patient for their participation
  "*Thanks for getting involved!*"

- Explain the next steps
  "*So what I will do now is:*
  - your goals will be entered into our database
  - we will review them together in three months
  - due to how the research is designed, we don't issue you a copy of the goals and it is ***not*** *sent to your GP or kidney specialist"*

- Provide their contact details
  "My contact details are ___________"

- Agree on a time for the next review meeting
  "*We need to set up a meeting to review your progress in three months. Would you like to make a date and time now, or should I contact you a few weeks prior?*"

A note on meetings held over Zoom

Appendix F, and the corresponding word version of it, provides a document that facilitators can use for meetings held over Zoom where they need to "share screen" to note down the brainstorming and goals.

After the meeting

Review

In some clinical or research settings there may be a requirement for a review of the goals to be undertaken before they are considered finalised. For the GOAL Trial a 'Hot Review' will occur for the first five participants completed by each facilitator. This is further explained below.

Write-up and distribution

The goals set during the meeting are to be populated into the template. If the facilitator's handwriting is legible then that may be sufficient enough without being typed up. The populated template is to be scanned into REDCap.

The data also needs to be inputted into a dedicated REDCap form.

As noted above, the goals will **not** be issued to the patient or to any clinicians given the GAS is being used as an outcome measure for the trial. The patient is welcome to write their own notes from the work undertaken. They are **not** permitted to take photos with their handheld devices.

Meeting with a patient to review their goals

Before the meeting

Location and timing

A 30 minute meeting should be scheduled to coincide with the review date set at the initial meeting (total meeting time of 60 minutes to cover the 30 minutes for GAS and 30 minutes for the other interval assessments). The review meeting is required to be as close as possible to the three-month review date from the initial meeting. It will occur in tandem with the other interval assessments including the Frailty Index and EQ-5D-5L.

Preparation

There is no set preparation required for the review meeting. When the facilitator contacts the patient to confirm their attendance at the meeting, it is important to have a relaxed and non-judgemental tone.

Practical tips to overcome patient's reticence of attending this meeting are discussed below in the "Approaches to troubleshooting common challenges" section.

During the meeting

Outlined below is a suggested approach a facilitator can take in meeting with a patient to review their goals.

Required material

- Pens and blank paper for notetaking
- Completed GAS template from initial meeting when baseline goals were set

Opening

In opening the meeting, the facilitator may wish to:

- Thank the patient for attending
  "*Thanks for coming along, I really appreciate your time*"
- Ask how they have found the time since last meeting
  "*How have you been since we last met?*"
- Outline the purpose and structure of the meeting
  "*Today what we are going to do is reflect on how you have progressed against your goals"*

Scoring goal attainment

With the facilitator using a printed copy of the completed GAS form from the initial meeting, they should work goal-by-goal to determine the outcome attained.

The facilitator should be mindful during this process to be as neutral as possible in their feedback. Any encouragement or support risks being an uncontrolled variable that may contaminate the results that follow at the six and twelve month mark.

With respects to the logistics of scoring, it is important to note that:

- Achieving "Expected level (0)" **is** a good outcome – they achieved their goal
- Performance level should be considered based on what they have done consistently in the past fortnight (depending on the measure), as opposed to what was done on one occasion or only attained when the patient was "having a good day". For example, if the goal is to walk 20 metres, consider asking whether they have been walking that distance most days in the last week. Further you would want to check that is not just the distance they are able to walk in a session with a physiotherapist at their side keeping them focussed and motivated.
- Where the patient's performance is between two predefined levels, they should be scored at the lower level[21].

To ensure no transcribing errors, the scores will be inputted directly into REDCap.

Resetting goals

Goals will **not** be reset at the review meeting. They remain unchanged for the subsequent six and twelve month review meetings, with no alteration of the scaling.

Review of action plans

At the review, patients may be particularly motivated to discuss with the facilitator how they could have better achieved their goals, or best achieve them from this point forward.

For consistency, there is no scope for facilitator input on a review of action plans. So as to not have patients feel unsupported, or risk fracturing the therapeutic relationship, the facilitator can verbally reiterate the talking points noted in the above "Action plans to achieve set goals" section.

Closing the meeting

In closing the meeting, the facilitator may wish to:

- Thank the patient for their participation
  "*Thanks for coming along today and participating in this process*"
- Offer some words of encouragement and praise, based on how the patient feels they went such as:
  "*Well done! I am so happy for you and the change you have made!* "
  (When a patient has met or exceeded their goals)
  or
  "*I think you made some great progress, and made some favourable changes even though it was not quite where you hoped to be.* "
  (When a patient did not meet their goals)
- Explain the next steps
  "*We will meet again at the six and twelve month anniversary of your goals being set.* "

After the meeting

The scores will have been inputted directly into REDCap.

The outcomes are not to be shared with any of the patient's care providers. Certainly the patient is able to share their progress with those they choose.

Calculating the overall GAS score

The extent to which goals are achieved is standardised by the formula:

$$GAS\ score = \frac{[50 + 10 \sum (w_i \times x_i)]}{\sqrt{[0.7 \sum w_i^2 + 0.3 (\sum w_i)^2]}}$$

(w_i = weight assigned to the goal area; x_i = the attained score for the goal area)

Using this formula, a score of 50 means that all goals have been met, whereas scores lower than 50 show a deficit in goal attainment and those above 50 show better than expected attainment[22]. The overall score is normalised to a mean of 50 and a standard deviation of 10.

A spreadsheet designed by Professor Turner-Stokes[22] to calculate scores is available online at:
https://www.kcl.ac.uk/cicelysaunders/attachments/tools-gas-calculation-sheet.xls

Note: The GAS score will be calculated by the trial's statistician and does not need to be generated by the facilitator.

Worked example of setting a goal

Worked examples of brainstorming for a goal, and populating them to the GAS template are provided in:

- Appendix D: Goal setting conversation starter – populated example
- Appendix B: Goal Attainment Scaling – populated example

Approaches to troubleshooting common challenges

The challenges of writing a goal

Two variables

A patient may state they "want to stop smoking so I can go running and not have to stop early". The goal as it is stated does not work for the GAS template as there are two variables (smoking cessation and running).

Further, complete cessation of smoking in three months, whilst possible, would warrant consideration of:

- How long has the patient managed not to smoke for in the past?
- When did the patient last try to quit smoking, and what was the outcome?
- Does the patient think it is more realistic for them to cut down the number of cigarettes or to focus on how many days they can go without a cigarette?

Suggested alternative

The goal can be broken down into two separate goals as outlined below. (Note the ranges used given there are continuous variables).

Domain	Health conditions	Hobbies
**Goal**	Reduce the number of cigarettes I am smoking	Run further without stopping
**Much less than expected (-2)**	Smoke >30 cigarettes a day	Run <3 minutes without stopping
**Somewhat less than expected (-1)**	Smoke 11-30 cigarettes a day (Current: 20 #)	Run 3-10 minutes without stopping (Current: 5 #)
**Expected level (0)**	Smoke 6-10 cigarettes a day	Run 10-12 minutes without stopping
**Somewhat better than expected (+1)**	Smoke 1-5 cigarettes a day	Run 13-15 minutes without stopping
**Much better than expected (+2)**	No longer smoke cigarettes	Run >15 minutes without stopping
**Importance (1-3)**	2	2
**Difficulty (1-3)**	3	2

Image 5*: Example goals.*

Unrealistic

Sometimes a patient may have a goal which appears unrealistic. For example, they may state they want to stop dialysis or to cure their cancer. Such goals are understandable and should be acknowledged as important. However, in the constraints of the GAS process they do not lend themselves to measures that can be assessed in a three-month period.

Suggested alternative

The facilitator can seek to unpack a goal such as “stop dialysis” with the patient by asking:

- *What do you dislike about dialysis?*
- *What does dialysis stop you from doing?*
- *What would you be doing if you were not on dialysis?*

This may identify an area for goal setting that is more suited to the GAS process which still is of importance to the patient.

It is also appropriate and important that, with the patient’s consent, the relevant doctor is notified about a patient’s pressing concerns if it falls outside the scope of the GAS process. For example, if they genuinely want to stop dialysis then they should be encouraged to discuss this with their nephrologist.

Too long-term

Some goals may require a longer timeframe to achieve than the three month review period. For example, someone may wish to ‘buy their own home”.

Suggested alternative

This ultimate goal can be framed alternatively as “save $<<amount>> for a deposit”, or even more simply as “save $<<amount>> to put towards my new home”.

Unable to articulate measures

Sometimes patients may have seemingly vague goals such as “to be a better person” or to “be a better Christian”. These can certainly be admirable, but for the purposes of GAS they are not specific or measurable enough.

Suggested alternative

The facilitator should explore with the patient what such statements means to them in practice. The following questions can be used when a goal seems too vague:

- *How do you observe others displaying the behaviour you aspire to?*
- *What does success look like to you?*
- *How will you know when you have achieved the outcome?*
- *At what point would you have a sense of pride with what you have achieved – what steps can we aim for and celebrate?*

Goals related to emotional distress

A patient may identify emotional distress as an area to address with their goal setting. If there is a situation in which the facilitator needs to intervene to actively assist the patient make an action plan to address this matter, then it is not appropriate for this to be a goal in this study. The reason is the active involvement in any action planning risks contaminating the intervention and causing bias.

See ‘Supporting participants in distress’ below.

The challenges of patient engagement

Non-attendance at meetings

If a patient avoids a meeting or declines to attend, the facilitator can consider seeking to understand why that is the case. It may be for practical reasons such as not having transport, or finding it too early in the day, or perhaps their support person is working and not free to accompany them. Where possible the facilitator should seek to problem solve the practicalities with them.

Alternatively, it may be due to poor motivation or from embarrassment that they have not achieved their goals. Discussed below are some suggestions on approaching these issues.

Poor motivation

A patient who lacks motivation is particularly challenging. The facilitator may seek to spend some time asking what is important to the patient or what they enjoy, and look to link goal setting to that. Keeping a focus on the benefits of goal setting may work well.

In some situations, the poor motivation may be a product of the patient being tired or stressed or feeling run down. Any underlying issue identified may then be conducive to having a goal written (such as “sleep better”), and thus help to engage the patient with something that is important to them.

Fear of failure

The act of setting a goal may be confronting for some patients as they fear failing or worry they will embarrass themselves by not achieving it. In those circumstances the focus of the facilitator could be on reassurance and providing a degree of emotional support. Praising the act of setting the goal or recognising any small progress, rather than focussing on whether or not an outcome was met, may work well too.

Overwhelmed

Goal setting may lead a patient to feel overwhelmed. If this occurs, the facilitator can seek to slow down the meeting, focus on one rather than five goals, and give space for the patient to gather themselves and be reassured.

Unfamiliarity of facilitator with subject matter

Some facilitators may feel ill prepared to set goals in an area which they have not had experience with before. The key in that situation is to:

- Harness the knowledge of the patient who may be able to provide valuable insights.
- Understand their past performance and its trajectory, to understand what is realistic.

For consistency and to prevent contamination of results, subject matter experts cannot be approached.

Poor literacy

Poor literacy, including health literacy, may be a barrier to discussing or writing goals, or even having a patient be able to read their goals at a later date. Having a support person may be useful, as may focusing on just one rather than five goals.

Culturally and linguistically diverse patients

An interpreter and/or a support person should be engaged if the patient demonstrates any difficulties in engaging with the facilitator, or in being able to articulate their thoughts in English. Setting aside a longer appointment may also be necessary.

Sensory impairments (hearing or vision)

Where a patient has visual or hearing impairments the meetings may need to be adapted to accommodate the patient’s individual needs.

Deterioration in health

During the follow-up period, patients may experience a decline in their health and functional status. This is not always unexpected given the trial’s population is frail older people. If a patient does experience a decline, goals remain unchanged and scoring is unmoderated. Other data collected will help account for this.

Supporting participants in distress

It is conceivable that participants may become upset during the GAS process. Discussing matters such as a loss of function or social isolation may cause distress. If this occurs, the facilitator should seek to provide emotional support initially. Depending on the situation, and the degree of distress, there should be a low threshold for referring the patient to a qualified professional. This may be the site's local psychologist or mental health team, or referral back to the GP for access to community-based services. If the research nurse, as the GAS facilitator, is unsure as to the appropriate action they are to contact the site's principal investigator for their input.

Actions to aid effective implementation of GAS

Training of facilitators

Ensuring the competence and confidence of facilitators is key to the success of GAS. This guide makes for a ready-to-use training tool to achieve that.

For the GOAL Trial, a training session will occur over Zoom with small groups attending.

Training records will be retained in the Trial Master File.

Simulation exercises

To allow facilitators to best feel prepared for administering GAS, it is strongly suggested they undertake a number of simulated cases. This allows them to more fully understand the tool and process, and how to adapt a patient's ideas into useable goal measures. Equally importantly, simulations allow them to experience dealing with scenarios where the (simulated) "patient" displays challenging behaviours such as being disengaged, frustrated or burdened with poor literacy.

For the GOAL Trial, simulations will be undertaken within the planned Zoom-based training sessions. The training participants will join small breakout groups and have the chance to rotate through the facilitator, patient and observer role.

At a short interval post the training, there will be one-on-one simulation exercises undertaken for the prospective facilitators to further embed their skills and have the opportunity to ask questions that may have arisen after a period of reflection and further reading.

Hot review

Whilst training-based simulations allow facilitators to attain a degree of proficiency with GAS, undertaking the process with real patients will potentially raise questions and challenges. To support facilitators, and ensure fidelity of the GAS process, a "Hot review" will be undertaken for the first few patient encounters undertaken by each facilitator.

A "Hot review" is a process whereby the facilitator sends their patient's goals to a colleague with more experience in GAS who can review the appropriateness of the measures and outcome scaling. It also offers an avenue for the facilitator to ask any questions that come up from their real-world experiences.

Key to the effectiveness of this review mechanism is a quick turnaround (ideally within days), so the patient can be reengaged by the facilitator in a timely manner whilst the discussions are still fresh in the mind of both facilitator and patient. It is a mechanism well suited to situations where GAS facilitators are geographically dispersed.

For the GOAL Trial, the first five GAS templates completed by each facilitator will be sent to the trial's PhD candidate or their delegate, with feedback being returned within five days.

It is also an optional support tool available throughout the trial at the facilitator's request.

References

1 Kiresuk, T. J. & Sherman, R. E. Goal attainment scaling: A general method for evaluating comprehensive community mental health programs. *Community Ment. Health J.* **4**, 443-453, doi:10.1007/bf01530764 (1968).

2 Moorhouse, P. *et al.* Treatment in a Geriatric Day Hospital improve individualized outcome measures using Goal Attainment Scaling. *BMC Geriatr.* **17**, doi:10.1186/s12877-016-0397-9 (2017).

3 Rockwood, K., Joyce, B. & Stolee, P. Use of goal attainment scaling in measuring clinically important change in cognitive rehabilitation patients. *J. Clin. Epidemiol.* **50**, 581-588, doi:10.1016/s0895-4356(97)00014-0 (1997).

4 van Seben, R., Reichardt, L., Smorenburg, S. & Buurman, B. Goal-setting instruments in geriatric rehabilitation: a systematic review. *Journal of Frailty & Aging* **6**, 37-45, doi:10.14283/jfa.2016.103 (2017).

5 Steenbeek, D., Ketelaar, M., Galama, K. & Gorter, J. W. Goal Attainment Scaling in paediatric rehabilitation: a report on the clinical training of an interdisciplinary team. *Child: Care, Health and Development* **34**, 521-529, doi:10.1111/j.1365-2214.2008.00841.x (2008).

6 McPherson, A. C. *et al.* A feasibility study using solution-focused coaching for health promotion in children and young people with Duchenne muscular dystrophy. *Dev. Neurorehabil.* **21**, 121-130, doi:10.1080/17518423.2017.1289271 (2018).

7 Kloseck, M. The use of Goal Attainment Scaling in a community health promotion initiative with seniors. *BMC Geriatr.* **7**, 16, doi:10.1186/1471-2318-7-16 (2007).

8 Verdoorn, S. *et al.* The use of goal attainment scaling during clinical medication review in older persons with polypharmacy. *Research in Social and Administrative Pharmacy* **15**, 1259-1265, doi:10.1016/j.sapharm.2018.11.002 (2019).

9 Zaza, C., Stolee, P. & Prkachin, K. The application of goal attainment scaling in chronic pain settings. *J. Pain Symptom Manage.* **17**, 55-64, doi:10.1016/s0885-3924(98)00106-7 (1999).

10 Berry, J. *et al.* A stepped wedge cluster randomised trial of a cognitive remediation intervention in alcohol and other drug (AOD) residential treatment services. *BMC Psychiatry* **19**, doi:10.1186/s12888-019-2044-4 (2019).

11 Roberts, J. C. *et al.* Goal Attainment Scaling for haemophilia (GAS-Hēm): testing the feasibility of a new patient-centric outcome measure in people with haemophilia. *Haemophilia* **24**, e199-e206, doi:10.1111/hae.13454 (2018).

12 Stolee, P., Stadnyk, K., Myers, A. M. & Rockwood, K. An Individualized Approach to Outcome Measurement in Geriatric Rehabilitation. **54**, M641-M647, doi:10.1093/gerona/54.12.m641 (1999).

13 Rockwood, K. *et al.* A Clinimetric Evaluation of Specialized Geriatric Care for Rural Dwelling, Frail Older People. **48**, 1080-1085, doi:10.1111/j.1532-5415.2000.tb04783.x (2000).

14 Rockwood, K. Appendix 2 - Attainment of treatment goals by people with Alzheimer's disease receiving galantamine: a randomized controlled trial. *Can. Med. Assoc. J.* **174**, 1099-1105, doi:10.1503/cmaj.051432 (2006).

15 Reuben, D. B. & Tinetti, M. E. Goal-Oriented Patient Care — An Alternative Health Outcomes Paradigm. *The New England Journal of Medicine* **366**, 777-779, doi:10.1056/NEJMp1113631 (2012).

16 Tinetti, M. E., Naik, A. D. & Dodson, J. A. Moving From Disease-Centered to Patient Goals–Directed Care for Patients With Multiple Chronic Conditions. *JAMA Cardiology* **1**, 9, doi:10.1001/jamacardio.2015.0248 (2016).

17 Stolee, P., Rockwood, K., Fox, R. A. & Streiner, D. L. The Use of Goal Attainment Scaling in a Geriatric Care Setting. *J. Am. Geriatr. Soc.* **40**, 574-578, doi:10.1111/j.1532-5415.1992.tb02105.x (1992).

18 Urach, S. *et al.* Statistical analysis of Goal Attainment Scaling endpoints in randomised trials. *Stat. Methods Med. Res.* **28**, 1893-1910, doi:10.1177/0962280218777896 (2019).

19 Morrow-Howell, N. *et al.* A Standardized Menu for Goal Attainment Scaling in the Care of Frail Elders. **38**, 735-742, doi:10.1093/geront/38.6.735 (1998).

20 Urquhart-Secord, R. *et al.* Patient and Caregiver Priorities for Outcomes in Hemodialysis: An International Nominal Group Technique Study. *Am. J. Kidney Dis.* **68**, 444-454, doi:10.1053/j.ajkd.2016.02.037 (2016).

21 Bovend'Eerdt, T. J., Botell, R. E. & Wade, D. T. Writing SMART rehabilitation goals and achieving goal attainment scaling: a practical guide. *Clin. Rehabil.* **23**, 352-361, doi:10.1177/0269215508101741 (2009).

22 Turner-Stokes, L. Goal attainment scaling (GAS) in rehabilitation: a practical guide. *Clin. Rehabil.* **23**, 362-370, doi:10.1177/0269215508101742 (2009).

Appendices

#	Document Name	Version	Date
A	Goal Attainment Scaling – blank template	01.01	05/10/20
B	Goal Attainment Scaling – populated example	01.01	05/10/20
C	GAS preparation information sheet	01.00	16/09/20
D	Goal setting conversation starter	01.00	14/12/20
E	Goal setting conversation starter – populated example	01.00	14/12/20
F	Zoom-based conversation starter & GAS template	01.00	14/12/20

Appendix A: Goal Attainment Scaling – blank template

Follows on next page.

Goals for <insert name>

Trial ID: __________

Date set: __________
Review date: __________

Timepoint: ☐ Baseline ☐ 3 months ☐ 6 months ☐ 12 months

**Domain**					
**Goal**					
**Much less than expected**<br><br>**(-2)**					
**Somewhat less than expected**<br><br>**(-1)**					
**Expected level**<br><br>**(0)**					
**Somewhat better than expected**<br><br>**(+1)**					
**Much better than expected**<br><br>**(+2)**					
**Importance (1-3)**					
**Difficulty (1-3)**					

# indicates baseline performance

(Scale for importance and difficulty: 1 = fairly; 2 = very; 3 = extremely.)

Appendix B: Goal Attainment Scaling – populated example

Follows on next page.

Goals for Joe Hammell Trial ID: 3234

Date set: 2 February 2021
Review date: 2 May 2021

Timepoint: ■ Baseline ☐ 3 months ☐ 6 months ☐ 12 months

**Domain**	Mobility	Community engagement			
**Goal**	To increase my endurance so I can walk further	To leave home more frequently so as to have more social interactions			
**Much less than expected (-2)**	I can walk 9 metres or less	I leave the house once a fortnight or less			
**Somewhat less than expected (-1)**	I can walk 10-19 metres (Current: 10 metres #)	I leave the house twice or three times a fortnight (Current: twice #)			
**Expected level (0)**	I can walk 20-29 metres	I leave the house four or five times a fortnight			
**Somewhat better than expected (+1)**	I can walk 30-39 metres	I leave the house six times a fortnight			
**Much better than expected (+2)**	I can walk 40 metres or more	I leave the house seven or more times a fortnight			
**Importance (1-3)**	2	3			
**Difficulty (1-3)**	2	1			

# indicates baseline performance *(Scale for importance and difficulty: 1 = fairly; 2 = very; 3 = extremely.)*

Appendix C: GAS preparation information sheet

Follows on next page.

GOAL
ENHANCING PATIENT-CENTRED CARE

<u>Appointment details</u>

Date: Time:

Location:

Research Nurse:

Contact Number:

We want to know about **YOUR** goals

When we next meet, we will be spending time together to learn more about you.

A key part of the GOAL Trial is setting goals with you. Don't worry – it is not hard.

By setting goals:

- Your care can be focused on what you want - not just what your doctors choose for you,
- You and your healthcare providers can engage in shared decision-making, and
- You can identify and celebrate meaningful changes you make in your life.

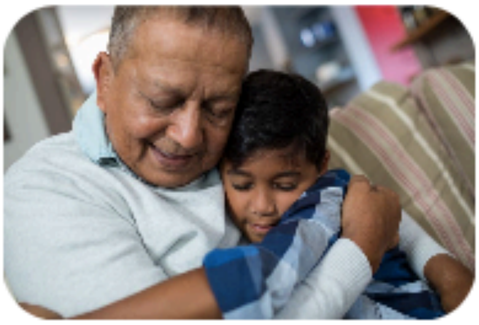

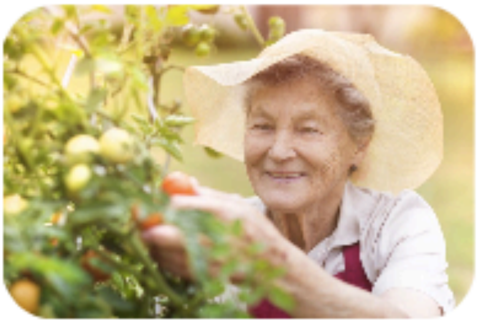

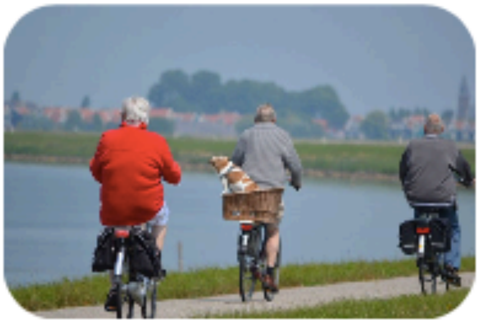

Before we meet, reflect on what parts of your life you may want to make better.
To help you, below are some areas people set goals in.

Physical health
- Medical conditions
- Medications (number, tolerability)
- Symptoms (such as fatigue or pain)
- Nutrition

Psychological health
- Cognition (memory, clarity of thought)
- Mood
- Resilience (your ability to cope)
- Sleep

Function
- Ability to mobilise (walk)
- Ability to care for yourself
- Transport

Planning
- Finances
- Plans for the future

Social engagement
- Personal relationship (spouse, dating)
- Relationships with your family
- Friendships
- Travel
- Employment and work roles
- Study
- Hobbies
- Community engagement (such as church groups or volunteering)

Your notes:

____

____

____

____

____

Appendix D: Goal setting conversation starter

Follows on next page.

Goal Setting Conversation Starter

Patient Details

Name: ________________________ Trial / Hospital ID: ________

Introduction

Thanks for participating – we are excited to undertake this process with you
You will be well supported; it will not be as hard as you may worry it is!
We think goals are important as it:
- helps to focus your care on what you value
- enables shared decision-making with your doctors
- identifies and celebrates meaningful change you make in your life

Introduce the GAS template

Take the patient through the template
1-5 goals to be set
Review period will be 3 months

Brainstorm

List below all possible goals

Select goals to further refine in the GAS template

1	
2	
3	
4	
5	

Appendix E: Goal setting conversation starter – populated example

Follows on next page.

Goal Setting Conversation Starter

Patient Details

Name: Joe Hammell Trial / Hospital ID: 3234

Introduction

Thanks for participating – we are excited to undertake this process with you
You will be well supported; it will not be as hard as you may worry it is!
We think goals are important as it:

- helps to focus your care on what you value
- enables shared decision-making with your doctors
- identifies and celebrates meaningful change you make in your life

Introduce the GAS template

Take the patient through the template
1-5 goals to be set
Review period will be 3 months

Brainstorm

List below all possible goals

Lose weight
Sleep better
Walk further
Be more social
See my grandkids more often
Go on a holiday to Darwin

Select goals to further refine in the GAS template

1	Have more endurance to be able to walk further
2	To get out of the house more to be more social
3	
4	
5	

Appendix F: Zoom-based conversation starter & GAS template

Follows on next page.

Brainstorm

List below all possible goals

Select goals to further refine in the GAS template

1	
2	
3	
4	
5	

Goals for <insert name>

Trial ID: __________

Date set: __________

Review date: __________

Timepoint: ☐ Baseline ☐ 3 months ☐ 6 months ☐ 12 months

**Domain**					
**Goal**					
**Much less than expected (-2)**					
**Somewhat less than expected (-1)**					
**Expected level (0)**					
**Somewhat better than expected (+1)**					
**Much better than expected (+2)**					
**Importance (1-3)**					
**Difficulty (1-3)**					

# indicates baseline performance

(Scale for importance and difficulty: 1 = fairly; 2 = very; 3 = extremely.)

ENHANCING PATIENT-CENTRED CARE
