# Supplementary material for: Training healthcare professionals to administer Goal Attainment Scaling as an outcome measure
Source: J Patient Rep Outcomes. 2024 Feb 26;8:22. doi: 10.1186/s41687-024-00704-0 (PMC10897066; doi:10.1186/s41687-024-00704-0)
Supplement: Supplementary file 5 — Supplementary File E: Links to video recordings [file 41687_2024_704_MOESM5_ESM.pdf]

GAS Training for the GOAL Trial – Links to video recordings

Comprehensive Geriatric Assessment for Frail Older People with Chronic Kidney Disease to Increase Attainment of Patient-Identified Goals - A Cluster Randomised Controlled Trial

Version control

Version: 01.00

Date: 14 December 2020

Recording of presentation from GAS classroom teaching

https://youtu.be/OHsAlrpW2Zk

Introduction and objectives: 0-3:45mins

Introduction to Goal Attainment Scaling: 3:45-6:11

Importance and benefits of goal setting: 6.11-7:29

Discuss SMART goals: 7:29-11:24

Overview of GAS template: 11:24-15:42

Describe how to set and scale a goal for GAS: 15:42-31:23

Action plan (after setting goal): 31:23-34:05

Suggest how to conduct meeting with patient: 34:15-37:52

Offer tips on troubleshooting common challenges (e.g. writing goals and engaging patient): 38:10-40:47

Describe the review and scoring process: 40:47-43:21

Next steps (after (watching the video) training):43:21-44:47

Demonstration video of an initial GAS goal setting meeting

https://youtu.be/BbcS30nvd5w
