# Supplementary material for: Training healthcare professionals to administer Goal Attainment Scaling as an outcome measure
Source: J Patient Rep Outcomes. 2024 Feb 26;8:22. doi: 10.1186/s41687-024-00704-0 (PMC10897066; doi:10.1186/s41687-024-00704-0)
Supplement: Supplementary file 6 — Supplementary File F: Worksheet for one-on-one feedback session [file 41687_2024_704_MOESM6_ESM.pdf]

Simulation scenario for one-on-one feedback session

Instructions

This exercise will offer the opportunity to work through the practical process of setting a goal using the Goal Attainment Scaling approach.

Required material:

- Blank Goal Attainment Scaling template
- Goal domains – patient version
- Goal setting conversation starter

The research nurse or their delegate will be the "facilitator" for the simulation. The GOAL Trial lead trainer, PhD candidate or delegate will assume the role of the "patient".

Timing:

- You will have 5 minutes initially, during which:
  - the facilitator should consider how they would like to structure their meeting, and
  - the patient should read through the briefing document and ready themselves to play their role
- There will be 10 minutes during which the facilitator will lead the patient in setting a goal
- To conclude, 5 minutes will be set aside for feedback and reflection:
  - use the "Keep doing", "Stop doing" and "Start doing" feedback approach so constructive feedback is provided (to be used by the participant acting as the facilitator when articulating self-reflection),
  - facilitator to share their self-reflection first, then the observer and patient to offer their thoughts

Note:

- You should set at least one goal by the end of this exercise, but in some situations setting more than one may be required if the conversation between facilitator and patient leads that way
- In setting the goal, you should also stipulate the importance and difficulty weighting

Briefing sheet for "Facilitator"

Scenario for one-on-one feedback session

You will shortly meet with a patient with the following demographics:

- 69 years of age
- Migrant from Italy in the 1960s as a child
- Independent with mobility and all activities of daily living
- Most significant health conditions are thalassaemia, emphysema and chronic kidney disease

Please work with them to set a goal, and weight it, using the Goal Attainment Scaling approach and template.

Briefing sheet for “Patient”

TO ONLY BE ISSUED TO THE INDIVIDUAL PLAYING THE “PATIENT” ROLE

Scenario for one-on-one feedback session

Demographics:

- 69 years of age
- Migrant from Italy in the 1960s as a child
- Independent with mobility and all activities of daily living
- Most significant health conditions are thalassaemia, emphysema and chronic kidney disease

Patient demeanour and mindset:

- Don’t really understand why you need to set goals
- Rather overwhelmed about having to have a meeting – never worked in an office environment and only sees the specialist once a year
- Despite this, is pleasant and well intentioned in interactions

Broad list of areas they thought they might set a goal in:

- Have more energy
- Be able to look after the grand kids more
- Help out at church activities
- Lose some weight

Ultimate goal you decide you want to set:

- See more of the grandkids

Relevant contextual information:

- Family is the priority to you, over everything else
- Have two children who live within 5 minutes drive, but they work fulltime and are busy
- Each of your children have their own two kids – aged from 8 to 10
- Your grandkids play soccer and go to ballet classes
- Currently just see your family for Sunday lunch at your home
- You don’t think it will be too difficult to achieve this goal, and think it is very important

(Note: Thalassaemia is a disorder that leads to anaemia (low blood count))

Take the liberty to make appropriate assumptions for any other details required.
