# Supplementary material for: Training healthcare professionals to administer Goal Attainment Scaling as an outcome measure
Source: J Patient Rep Outcomes. 2024 Feb 26;8:22. doi: 10.1186/s41687-024-00704-0 (PMC10897066; doi:10.1186/s41687-024-00704-0)
Supplement: Supplementary file 7 — Supplementary File G: Survey questions [file 41687_2024_704_MOESM7_ESM.pdf]

Surveys

#	Document Name	Version Number	Date
A	Pre-training survey	01.00	10/11/20
B	Post training survey	01.00	10/11/20

A: Pre-training survey

I had heard of Goal Attainment Scaling before being engaged in the GOAL Trial:

☐ Yes ☐ No

I have used Goal Attainment Scaling before (either as a facilitator or participant):

☐ Yes ☐ No

I have heard of SMART (Specific Measurable Achievable Relevant Timebound) goals before:

☐ Yes ☐ No

I have set my own SMART goals before (either personal or professional life):

☐ Yes ☐ No

I have facilitated the setting of goals before (i.e. helped someone else set a formal goal):

☐ Yes ☐ No

Please indicate your response to each of the following statements

	Strongly disagree	Disagree	Neutral	Agree	Strongly agree
Goal setting is important for patient-centred care	☐	☐	☐	☐	☐
Goal setting benefits a patient’s health	☐	☐	☐	☐	☐
Goal setting benefits a patient’s quality of life	☐	☐	☐	☐	☐
At this moment, I feel able to…					
write a SMART goal	☐	☐	☐	☐	☐
define an outcome measure for a goal	☐	☐	☐	☐	☐
scale a goal to meet the requirements of the GAS template	☐	☐	☐	☐	☐
structure a conversation with a patient to set their goals	☐	☐	☐	☐	☐
troubleshoot a patient’s desired goal which does not meet the GAS requirements	☐	☐	☐	☐	☐
work with a patient who has poor engagement with the process	☐	☐	☐	☐	☐

Please include any comments relevant to your responses above.

B: Post training survey

You have now completed the training program, which comprised of:

- Formal 'virtual' classroom teaching
  (including theory components and an initial practical simulation)
- Self-directed reflection and reading
  (primarily supported by the training manual "A practical guide to administering Goal Attainment Scaling for the GOAL Trial", as well as recorded conversations)
- One-on-one simulation and feedback session
- Hot review
  (of goals completed with first five patients)

Please indicate your response to each of the following statements

	Strongly disagree	Disagree	Neutral	Agree	Strongly agree
Goal setting is important for patient-centred care	☐	☐	☐	☐	☐
Goal setting benefits a patient's health	☐	☐	☐	☐	☐
Goal setting benefits a patient's quality of life	☐	☐	☐	☐	☐
At this moment, I feel able to…					
write a SMART goal	☐	☐	☐	☐	☐
define an outcome measure for a goal	☐	☐	☐	☐	☐
scale a goal to meet the requirements of the GAS template	☐	☐	☐	☐	☐
structure a conversation with a patient to set their goals	☐	☐	☐	☐	☐
troubleshoot a patient's desired goal which does not meet the GAS requirements	☐	☐	☐	☐	☐
work with a patient who has poor engagement with the process	☐	☐	☐	☐	☐

Please include any comments relevant to your responses above.

Please indicate how satisfied you were with the components of the training program.

	Very dissatisfied	Dissatisfied	Neither satisfied or dissatisfied	Satisfied	Very satisfied
Formal 'virtual' classroom teaching	☐	☐	☐	☐	☐
Training manual	☐	☐	☐	☐	☐
Recordings of example goal-setting conversations	☐	☐	☐	☐	☐
One-on-one simulation and feedback session	☐	☐	☐	☐	☐
Hot review	☐	☐	☐	☐	☐

Formal 'virtual' classroom teaching — Comments (incl: what worked well, what needs revision)

____

____

Training manual — Comments (incl: what worked well, what needs revision)

____

____

Recordings of example goal-setting conversations — Comments (incl: what worked well, what needs revision)

____

____

One-on-one simulation and feedback session — Comments (incl: what worked well, what needs revision)

____

____

Hot review — Comments (incl: what worked well, what needs revision)

____

____

Please note any other comments or thoughts you wish to share:

____

____
