# Supplementary material for: Training healthcare professionals to administer Goal Attainment Scaling as an outcome measure
Source: J Patient Rep Outcomes. 2024 Feb 26;8:22. doi: 10.1186/s41687-024-00704-0 (PMC10897066; doi:10.1186/s41687-024-00704-0)
Supplement: Supplementary file 8 — Supplementary File H: Consent Statement [file 41687_2024_704_MOESM8_ESM.pdf]

Consent statement

(Will appear at the start of each of the online surveys)

Training of research staff in Goal Attainment Scaling administration: An evaluation

Principal investigators:
Dr Benignus Logan, Dr Andrea Viecelli, Ms Bonnie Pimm, Ms Laura Robison, Prof David Johnson, Ms Elaine Pascoe, Prof Ruth Hubbard

Context

Prior to the GOAL Trial, there was no comprehensive training resource to enable health professionals to confidently and consistently administer the Goal Attainment Scaling tool in a standardised manner. To meet this need, a training program has been written.

The purpose of this study is to evaluate the training program's success in readying attendees to administer the Goal Attainment Scaling tool with GOAL Trial research participants.

It is anticipated that the training program, and the learnings from this study evaluating it, will enable clinicians and researchers to successfully deploy the Goal Attainment Scaling tool in situations where patient-identified goals can better facilitate patient-centred care and personalised medicine.

What we are seeking from you

We are inviting you to participate in this survey as you are attending the training for Goal Attainment Scaling. All attendees of the training are eligible for recruitment.

There will be two short surveys. They are being administered on the secure Qualtrics platform. One is to be completed at the start of the classroom teaching (pre-training), and one is to be completed after the conclusion of your Hot reviews (post training).

We are inviting you to comment on the training program's effectiveness and how you perceive it to have prepared you for administering the Goal Attainment Scaling tool in the GOAL Trial.

We are inviting you to comment on the training program's effectiveness and how you perceive it to have prepared you for administering the Goal Attainment Scaling tool in the GOAL Trial.

Further details on confidentiality and ethical principles

Participation in this study is voluntary. Completion of the survey will be interpreted as consent to participate. Data is deidentified. There is no way of withdrawing consent once the survey responses are submitted.

The study involves two surveys, anticipated to equate to a total of 10 minutes of your time.

All responses are anonymous. Any identifiable information provided in qualitative responses will be deidentified before analysis and reporting. Results from the research are intended for publication in medical and scientific journals. In any publication, information will be provided in such a way that you cannot be identified.

This study has been approved by the Metro South Human Research Ethics Committee (Reference: HREC/2020/QMS/70496).

Are you willing to participate in this survey?

YES *NO*

Linkage of surveys

We ask that you enter your name, in order to link the responses of the pre and post training surveys. It will be deidentified, and replaced by a code, before the data is released to the investigators. This task will be undertaken by an independent Clinical Research Associate from AKTN not associated with this study.

Are you willing to enter your name to allow linkage of surveys (completion indicates consent)?
Note: You can still complete the survey without providing your name if you prefer.

Name: ________________

Key contacts

Clinical contact person

Name:	Dr Benignus Logan
Position:	PhD Candidate
Telephone:	0407 125 182
Email:	benignus.logan@uq.edu.au

Complaints contact person

Name:	Governance Officer
Position:	Governance Officer
Telephone:	07 3443 8050
Email:	MSH-RGO@health.qld.gov.au

Reviewing HREC approving this research and the HREC Executive Officer details

Reviewing HREC name:	Metro South HREC (00167)
HREC Executive Officer:	HREC Coorindator
Telephone:	07 3443 8047
Email:	MSH-Ethics@health.qld.gov.au
